# Whole genome profiling of short-term hypoxia induced genes and identification of HIF-1 binding sites provide insights into HIF-1 function in *Caenorhabditis elegans*

**DOI:** 10.1371/journal.pone.0295094

**Published:** 2024-05-14

**Authors:** Dingxia Feng, Long Qu, Jo Anne Powell-Coffman

**Affiliations:** 1 Department of Genetics, Development and Cell Biology, Iowa State University, Ames, Iowa, United States of America; 2 Department of Statistics, Iowa State University, Ames, Iowa, United States of America; INSERM U869, FRANCE

## Abstract

Oxygen is essential to all the aerobic organisms. However, during normal development, disease and homeostasis, organisms are often challenged by hypoxia (oxygen deprivation). Hypoxia-inducible transcription factors (HIFs) are master regulators of hypoxia response and are evolutionarily conserved in metazoans. The homolog of HIF in the genetic model organism *C*. *elegans* is HIF-1. In this study, we aimed to understand short-term hypoxia response to identify HIF-1 downstream genes and identify HIF-1 direct targets in *C*. *elegans*. The central research questions were: (1) which genes are differentially expressed in response to short-term hypoxia? (2) Which of these changes in gene expression are dependent upon HIF-1 function? (3) Are any of these *hif-1*-dependent genes essential to survival in hypoxia? (4) Which genes are the direct targets of HIF-1? We combine whole genome gene expression analyses and chromatin immunoprecipitation sequencing (ChIP-seq) experiments to address these questions. In agreement with other published studies, we report that HIF-1-dependent hypoxia-responsive genes are involved in metabolism and stress response. Some HIF-1-dependent hypoxia-responsive genes like *efk-1* and *phy-2* dramatically impact survival in hypoxic conditions. Genes regulated by HIF-1 and hypoxia overlap with genes responsive to hydrogen sulfide, also overlap with genes regulated by DAF-16. The genomic regions that co-immunoprecipitate with HIF-1 are strongly enriched for genes involved in stress response. Further, some of these potential HIF-1 direct targets are differentially expressed under short-term hypoxia or are differentially regulated by mutations that enhance HIF-1 activity.

## Introduction

Oxygen is essential to aerobic organisms for energy production and cellular redox environment maintenance [1]. During development, disease and homeostasis, animals are often challenged by oxygen deprivation (hypoxia). In mammals, the majority of transcriptional responses to hypoxia are mediated by the hypoxia-inducible transcription factors (HIFs), and deeper understanding of these regulatory networks may inform therapeutic interventions for common hypoxia-related diseases, such as cancer, arthritis and ischemia. The heterodimeric HIF complexes consist of α and β subunits, and both subunits are bHLH (basic-helix-loop-helix)-PAS (PER/ARNT/SIM) domain proteins [1,2]. There are three HIFα and three HIFβ homologs in the human genome [3]. HIFβ has multiple bHLH-PAS dimerization partners and is relatively stable and abundant. In contrast, HIFα is short-lived under well-oxygenated conditions and is dedicated to hypoxia response [4–6]. The stability of mammalian HIFα is regulated by the PHD-VHL pathway. When oxygen levels are high enough, HIFα is hydroxylated by prolyl hydroxylase proteins (PHDs), in reactions that require oxygen as substrate. The hydroxylated HIFα is then targeted by the E3 ligase VHL (von Hippel-Lindau tumor suppressor) for proteasomal degradation.

The nematode *C*. *elegans* is a powerful genetic model organism for studying the HIF regulatory networks [7]. The *C*. *elegans* genome encodes single homologs for HIFα and HIFβ, named *hif-1* and *aha-1*, respectively. While *hif-1*α -/- mice die by E9.0 with severe vascular defects [8,9], *C*. *elegans hif-1*(*ia04*) loss-of-function mutants survive and develop normally in normoxia but are defective in hypoxia adaption [10–12]. As in mammals, the stability of HIF-1 protein is regulated by oxygen levels [11,13,14]. The PHD protein that encodes the HIF hydroxylase and the VHL E3 ligase are named EGL-9 and VHL-1, respectively [13]. Interestingly, prior studies have shown that some HIF-1 target genes are expressed at higher levels in *egl-9*-deficient animals, compared to animals lacking *vhl-1* function [15,16]. This suggests that EGL-9 down-regulates HIF-1 through multiple mechanisms.

Prior studies [17,18] have described oxygen-dependent changes in *C*. *elegans* gene expression, but the direct targets of HIF-1 are not fully described. Here, we identify DNA sequences that are bound by and can be co-immunoprecipitated with HIF-1. To better understand which of the genes bound by HIF-1 are involved in short-term hypoxia response, we also identify genes that are differentially regulated by short-term (2 hours) hypoxia treatments and ask whether some of the HIF-1 targets are essential to survival in hypoxic conditions.

## Results

### Identification of genes responsive to short-term hypoxia treatment

To define the immediate gene expression changes caused by hypoxia, we treated L4-stage wild-type N2 worms with 0.5% oxygen for 2 hours and compared the whole genome gene expression profile under hypoxia with that in normoxia. The complete analysis results are provided in S1 Table. Among the 18,011 unique genes assayed, 681 genes exhibited significant changes in mRNA expression in response to hypoxia: 437 genes were up-regulated (S2 Table), and 244 genes were down-regulated (S3 Table). We employed the WormCat tool [19] to describe the physiological functions that were enriched among these co-regulated gene sets. This analysis identified three broad categories of functions enriched among genes that were up-regulated in response to short-term hypoxia: stress response, metabolism and transcription factors. The genes that were expressed at lower levels in response to hypoxia were enriched for the functional categories of metabolism, transmembrane transport and stress response. Subcategories and the genes assigned to them are listed in S4 and S5 Tables.

We anticipated that our dataset would include genes that had been shown to be hypoxia-responsive in other published studies. RNase protection, RNA blot and real-time qRT-PCR assays had established that F22B5.4, *nhr-57*, *fmo-12*/*fmo-2*, *egl-9*, *phy-2*, *cah-4*, K10H10.2/*cysl-2*, F26A3.4, C12C8.1/*hsp-70*, *acs-2* and *pck-1* were induced by hypoxia in N2 wild-type animals [17,18,20,21]. As expected, the microarray analyses described herein identified these genes as induced by 2 hours of hypoxia in N2 (S2 Table). We also compared this dataset to a prior microarray study that identified 490 genes as hypoxia responsive when L3-stage N2 worms were treated with 0.1% oxygen for 4 hours [17]. While the larval stage and duration of hypoxia treatment were different, 50 genes exhibited hypoxia-dependent changes in gene expression in these two experiments. The overlap is significant (*p*-value = 2.46E-10, by Fisher’s exact test).

#### HIF-1-dependent short-term hypoxia responses

**Fig 1:**
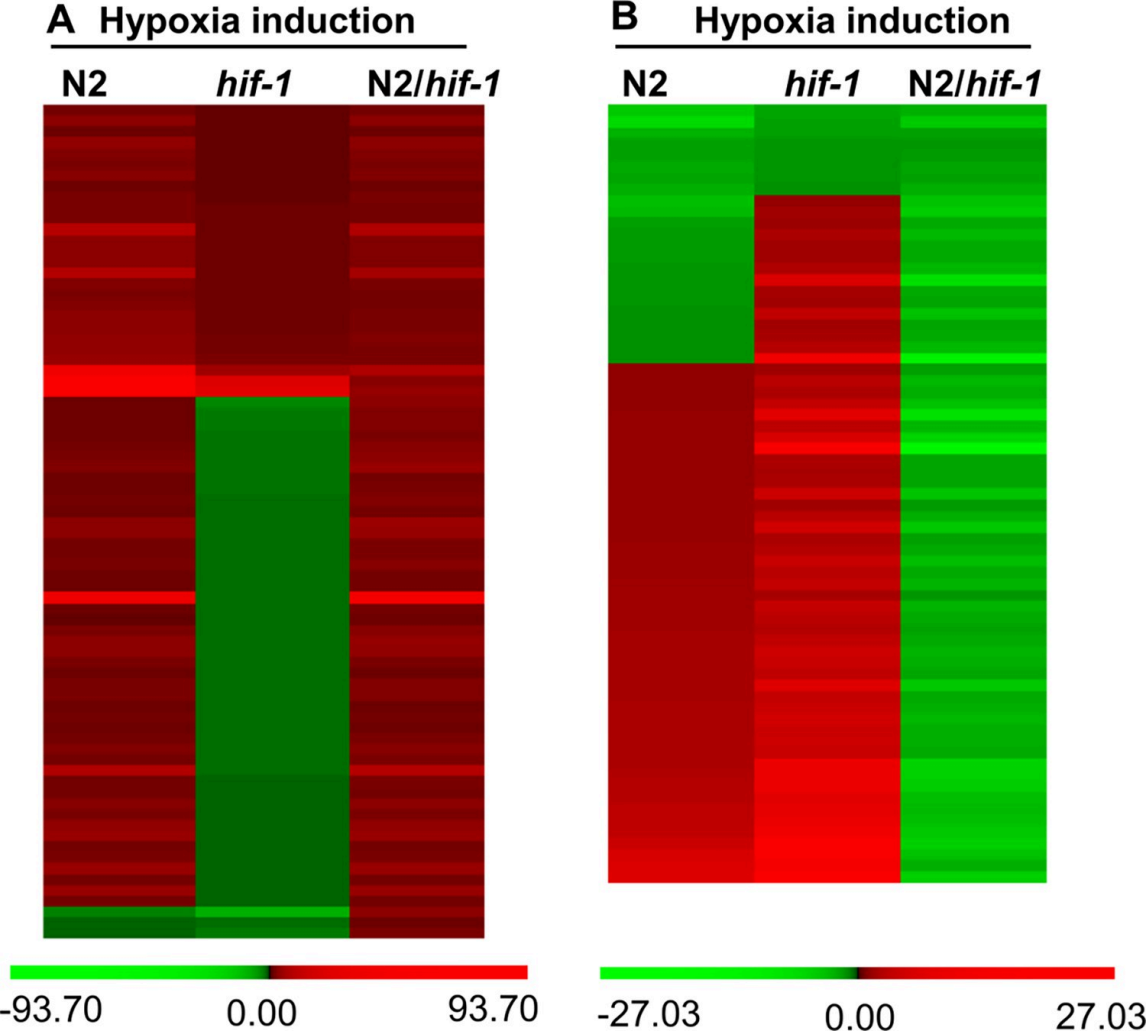
Hypoxia inductions of genes regulated by HIF-1 under hypoxia. (A, B) The heat map illustrations of hypoxia-dependent changes in gene expression for which HIF-1 was a positive regulator (A) or a negative regulator (B). Values < 0 are green, values > 0 are red. The color intensities correspond to the induction levels in S6 (for Fig 1A) and S7 (for Fig 1B) Tables.

We next asked which short-term hypoxia-responsive gene expression changes were dependent upon HIF-1. To do this, we compared the hypoxia responses in N2 and *hif-1(ia04)* loss-of-function mutants. If a gene was positively regulated by HIF-1 under hypoxia, then its hypoxia induced fold change in N2 (N2 hypoxia/N2 normoxia) would be at least 1.6-fold higher than that in *hif-1(ia04)* animals [*hif-1(ia04)* hypoxia/*hif-1(ia04)* normoxia] (*q*-value ≤ 5%). If a gene was negatively regulated by HIF-1 under hypoxia, then its hypoxia induction in N2 (N2 hypoxia/N2 normoxia) would be at least 1.6-fold lower than that in *hif-1* mutants [*hif-1(ia04)* hypoxia/*hif-1(ia04)* normoxia] (*q*-value ≤ 5%). Totally, we identified 124 genes whose hypoxia responses were different in *hif-1(ia04)* relative to N2 (S6 and S7 Tables). Among these, 64 genes were positively regulated by HIF-1 under hypoxia (upregulation was higher in N2 than in *hif-1(ia04)* in hypoxic conditions) (S6 Table), and 60 genes were negatively regulated by HIF-1 under hypoxia (downregulation was stronger in N2 than in *hif-1(ia04)* in hypoxic conditions) (S7 Table). The heat maps in Fig 1 illustrate their hypoxia inductions in N2 and *hif-1(ia04)*, and the relative inductions (N2/*hif-1(ia04)*).

The genes positively regulated by HIF-1 are enriched for two broad categories, metabolism and stress response. WormCat analyses also identified subcategories of enriched functions (Cat 2 and Cat 3), and these are listed in Tables 1 and S8. Several genes with important functions drew our attention. For example, *cysl-2*, *ethe-1* and *sqrd-*1 had been shown to have roles in detoxifying hydrogen sulfide (H_2_S) and hydrogen cyanide (HCN) [22], and *sqrd-*1 also maintained translation in H_2_S [23]. *mce-1*, *mmcm-1*, ZK550.6, *acox-1*.*6*, *gbh-2* and *fat-5* had been shown to be involved in lipid metabolism, and *asns-2*, *cysl-*2 and *ddo-1* had central roles in amino acid metabolism. *pck-*1 was integral to gluconeogenesis [21,24], and *fmo-1* and *fmo-2* were involved in phase I detoxification. The *phy-2* (prolyl 4-hydroxylase) had been shown to have roles in cuticle collagen synthesis [25]. These findings were well aligned with previously published real-time qRT-PCR experiments that had shown that *pck-1*, *cysl-2* and *phy-2* were positively-regulated by HIF-1 under hypoxia [21].

**Table 1:** Enriched biological terms for genes positively regulated by HIF-1 under short-term hypoxia.

Biological term	Count	Bonferroni FDR	Genes
Cat1: Metabolism	27	2.54E-13	*pah-1*, *fat-5*, *fmo-1*, *fmo-2*, *gbh-2*, *hgo-1*, *mai-1*, *stdh-1*, *ethe-1*, *ddo-1*, *mce-1*, *acox-1*.*6*, *mpst-3*, *cysl-2*, Y105C5B.9, ZK550.6, *mmcm-1*, *acl-12*, C32E8.9, F41E6.5, F57B9.1, F58A6.1, K07B1.4, *asns-2*, *eppl-1*, *pck-1*, Y53G8B.2
Cat1: Stress response	8	7.88E-03	*gst-19*, *hsp-12*.*3*, *lec-8*, C32H11.4, *ugt-33*, F01D5.5, *sqrd-1*, H20E11.3
Cat2: Metabolism: lipid	10	1.28E-05	*fat-5*, *stdh-1*, *mce-1*, *acox-1*.*6*, *acl-12*, C32E8.9, F58A6.1, K07B1.4, *eppl-1*, Y53G8B.2
Cat2: Metabolism: amino acid	5	4.03E-05	*pah-1*, *hgo-1*, *ddo-1*, *cysl-2*, *asns-2*
Cat2: Metabolism: FMO	2	2.62E-03	*fmo-1*, *fmo-2*
Cat3: Metabolism: lipid: beta oxidation	4	1.19E-03	*mce-1*, *acox-1*.*6*, C32E8.9, F58A6.1
Cat3: Metabolism: FMO	2	3.53E-03	*fmo-1*, *fmo-2*
Cat3: Metabolism: amino acid: synthesis	3	3.88E-03	*pah-1*, *cysl-2*, *asns-2*

The genes that were negatively regulated by HIF-1 were also enriched for the broad categories of stress response and metabolism (listed as Cat1 functions in Tables 2 and S9). Most of the genes are identified as having metabolic functions, especially lipid and amino acid metabolism.

**Table 2:** Enriched biological terms for genes negatively regulated by HIF-1 under short-term hypoxia.

Biological term	Count	Bonferroni FDR	Genes
Cat1: Stress response	14	6.95E-08	*gst-9*, *parg-2*, *tir-1*, C10C5.2, *ugt-44*, F13A7.11, F17C11.11, F18H3.4, *ttc-36*, *ugt-25*, *ugt-28*, *irg-2*, F41B4.3, T24C4.4
Cat1: Metabolism	13	4.75E-04	*ckb-2*, *elo-2*, *sur-5*, *plin-1*, C06B3.6, *oac-14*, *acs-2*, *acdh-2*, *pgph-3*, *ddo-2*, *hacd-1*, Y77E11A.2, ZK1290.5
Cat2: Metabolism: lipid	10	5.18E-06	*ckb-2*, *elo-2*, *sur-5*, *plin-1*, C06B3.6, *oac-14*, *acs-2*, *acdh-2*, *pgph-3*, *hacd-1*
Cat2: Stress response: pathogen	6	7.39E-05	*tir-1*, C10C5.2, F13A7.11, F18H3.4, *irg-2*, T24C4.4
Cat2: Stress response: unassigned	3	3.14E-03	*parg-2*, F17C11.11, *ttc-36*
Cat3: Stress response: pathogen: unassigned	5	6.90E-05	C10C5.2, F13A7.11, F18H3.4, *irg-2*, T24C4.4
Cat3: Stress response: unassigned	3	5.03E-03	*parg-2*, F17C11.11, *ttc-36*

### Genes responsive to both short-term and persistent HIF-1 activities

We anticipated that some of the genes that were responsive to short-term hypoxia would also be differentially expressed in mutants that over-expressed HIF-1 targets. To explore this question further, we compared the findings summarized in S6 and S7 Tables (genes positively or negatively regulated by HIF-1 in 2-hour hypoxia treatments) with genes that were mis-regulated in the four HIF-1 negative regulator mutants [*vhl-1(ok161)*, *rhy-1(ok1402)*, *egl-9(sa307)*, and *swan-1(ok267);vhl-1(ok161)* double mutants; See the related study [26] and S1 Table]. We identified 23 genes were positively regulated by HIF-1 under short-term hypoxia and up-regulated in all the four HIF-1 negative regulator mutants (Table 3). We identified 3 genes that were negatively regulated by HIF-1 under short-term hypoxia and down-regulated in all the four mutants (Table 4). The molecular functions of the 23 genes positively regulated by both short-term and persistent HIF-1 activities were diverse, including genes for lipid metabolism (*mce-1*, *mmcm-1*, ZK550.6 and *gbh-2*), H_2_S and HCN detoxification (*cysl-2*, *ethe-1* and *sqrd-*1), gluconeogenesis (*pck-1*), and protein synthesis regulation (*efk-1*), as well as collagen synthesis (*phy-2*), and others. The 3 genes negatively regulated by both short-term and persistent HIF-1 activities were T28A11.2 (hypothetical protein), *acdh-2* (Acyl CoA dehydrogenase), and *acs-2* (fatty acid CoA synthetase family).

**Table 3:** Hypoxia responses of genes positively regulated by both short-term and persistent HIF-1 activities.

Probeset ID	Gene	Description	Hypoxia induction in N2	Hypoxia induction in *hif-1*	Relative induction (N2/*hif-1*)	*q*-value
175837_s_at	*efk-1*	Elongation factor-2 kinase	3.16	1.31	2.40	2.31E-02
187973_s_at	*egl-9*	Dioxygenase	3.41	1.04	3.27	7.05E-04
191695_at	*mce-1*	Methylmalonyl-CoA epimerase	2.32	1.05	2.22	7.31E-05
191736_s_at	*mmcm-1*	Methylmalonyl-CoA mutase	1.69	-1.03	1.74	1.45E-02
177532_at	F22B5.4	Integral membrane protein	3.46	-1.04	3.61	3.53E-03
188978_s_at	K10H10.2/*cysl-2*	Cysteine synthase	3.28	-1.43	4.69	1.47E-03
180842_s_at	*ethe-1*	Sulfur dioxygenase	3.51	1.54	2.27	5.46E-05
178043_at	*sqrd-1*	Sulfide quinone oxidoreductase	8.55	1.15	7.43	3.36E-06
173335_s_at	*dod-3*	Downstream Of DAF-16 (regulated by DAF-16)	57.42	20.34	2.82	1.65E-02
174073_at	*tts-1*	Transcribed telomere-like sequence	93.70	23.24	4.03	2.60E-02
193464_s_at	*phy-2*	Collagen prolyl 4-hydroxylase	7.44	-1.10	8.22	7.38E-05
193914_at	*gbh-2*	Gamma butyrobetaine hydroxylase	3.23	-1.21	3.91	1.36E-04
190962_s_at	*gst-19*	Glutathione S-transferase	1.85	-1.18	2.19	9.13E-03
192338_at	*hsp-12*.*3*	Heat shock protein	2.85	-1.06	3.01	8.99E-03
180909_at	C32H11.4	Hypothetical protein	2.55	-1.59	4.06	1.40E-03
182532_at	*mpst-3*	Mercaptopyruvate sulfurtransferase homolog	1.24	-1.44	1.79	1.66E-03
182849_at	Y37A1B.5	Selenium binding protein	3.04	1.03	2.95	1.97E-04
186811_s_at	ZK550.6	Peroxisomal phytanoyl-CoA hydroxylase	4.26	-1.02	4.35	2.01E-05
173475_s_at	*glb-1*	Globin	3.07	-1.19	3.66	2.24E-05
186801_s_at	F45D11.14	Hypothetical protein	35.75	4.87	7.34	1.33E-03
182747_at	R08E5.3	Methyltransferase	3.03	1.43	2.12	2.96E-02
192581_s_at	W05G11.6/*pck-1*	Phosphoenolpyruvate carboxykinase (PEPCK)	3.10	-1.41	4.36	1.03E-02
184714_s_at	Y53G8B.2	2-acylglycerol O-acyltransferase	3.16	1.29	2.46	4.75E-04

**Table 4:** Hypoxia responses of genes negatively regulated by both short-term and persistent HIF-1 activities.

Probeset ID	Gene	Description	Hypoxia-induction in N2	Hypoxia induction in *hif-1*	Relative induction (N2/*hif-1*)	*q*-value
184849_at	T28A11.2	Hypothetical protein	-1.28	1.47	-1.88	1.28E-02
173996_at	*acdh-2*	Acyl CoA dehydrogenase	1.05	3.22	-3.08	1.17E-02
174675_at	*acs-2*	Fatty acid CoA synthetase family	5.29	16.74	-3.17	1.07E-02

### Genes regulated by HIF-1 and hypoxia overlap with genes regulated by H_2_S

Prior studies have shown that H_2_S treatment increased HIF-1 protein levels, and *hif-1*-deficient animals were less able to survive H_2_S treatment [22,27]. This prompted us to ask whether gene expression changes caused by hypoxia were similar to those caused by H_2_S. We compared our dataset with the microarray studies measuring gene expression changes after 1 hour or 12 hours of H_2_S treatment [28].

**Fig 2:**
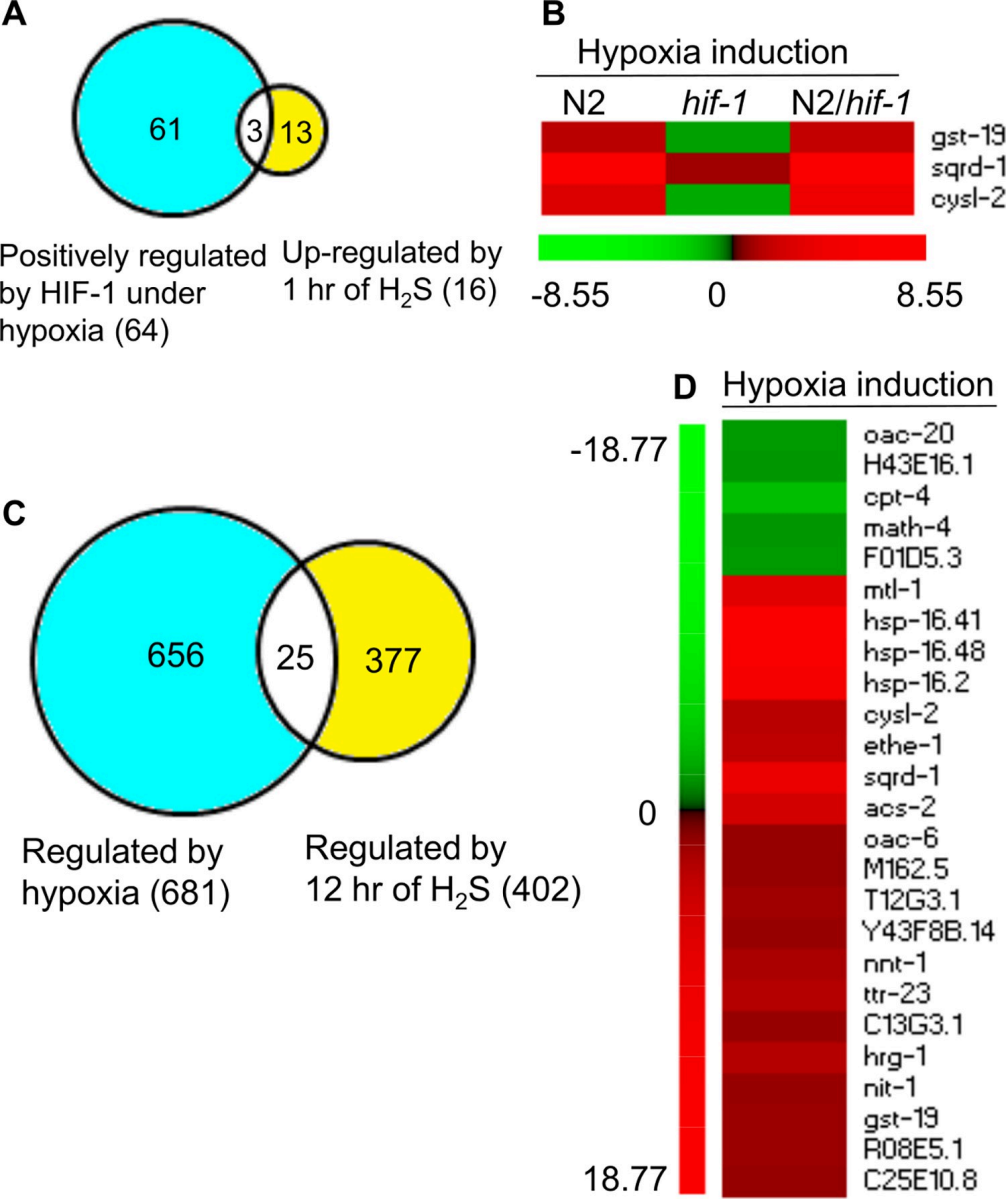
Genes responsive to HIF-1 and hypoxia overlap with genes responsive to H_2_S. (A) Three genes were previously shown to be up-regulated by 1 hour of H_2_S treatment and are positively regulated by HIF-1 under hypoxia (*p*-value = 4.04E-05, by Fisher’s exact test). (B) The heat map shows the hypoxia-induced changes in mRNA levels for these 3 genes in N2 and *hif-1(ia04)* mutants, and the relative inductions (N2/*hif-1(ia04)*). The numeric values are in S6 Table. (C) The mRNA levels of 25 genes were changed by both short-term hypoxia treatment and 12 hours of H_2_S treatment (*p*-value = 1.92E-04, by Fisher’s exact test). (D) The heat map shows the hypoxia-induced changes in gene expression for these 25 genes in N2 animals. The numeric values are in S2 (for genes up-regulated by hypoxia) and S3 (for genes down-regulated by hypoxia) Tables. In (B) and (D), values < 0 are green, values > 0 are red. The color intensities correspond to the induction levels.

Of the 16 genes that were found to be up-regulated in wild-type animals following 1-hour exposure to H_2_S [28], three of the genes (*gst-19*, F02H6.5/*sqrd-1* and K10H10.2/*cysl-2)* are also identified herein as positively regulated by HIF-1 in hypoxic conditions (Fig 2A).The overlap is significant (*p*-value = 4.04E-05, by Fisher’s exact test). The *gst-19* gene functions in phase II detoxification, and *sqrd-1* and *cysl-2* have been shown to have important roles in H_2_S and HCN detoxification and proteostasis [22,23]. These findings are consistent with previous qRT-PCR assays showing that H_2_S exposure increased the mRNA levels of *sqrd-1* and *cysl-2* in a HIF-1-dependent manner [22,27,28]. The heat map in Fig 2B illustrates how hypoxia changes expression of these 3 genes in N2 and *hif-1(ia04)*. Twelve hours of H_2_S treatment had been shown to cause 402 changes in gene expression in N2 adults [28]. Here, we report that 25 of these genes also exhibited expression changes in response to short-term hypoxia (Fig 2C). The overlap is significant (*p*-value = 1.92E-04, by Fisher’s exact test), and it includes genes involved in phase II detoxification (*gst-19*, *hsp-16*.*41*, *hsp-16*.*48* and *hsp-16*.*2)* and genes required for survival in H_2_S (*sqrd-1*, C33A12.7/*ethe-1* and *cysl-2)* [22,23,27]. The heat map in Fig 2D illustrates the hypoxia-induced changes in gene expression for these 25 genes.

### Genes regulated by HIF-1 and hypoxia overlap with genes regulated by DAF-16

**Fig 3:**
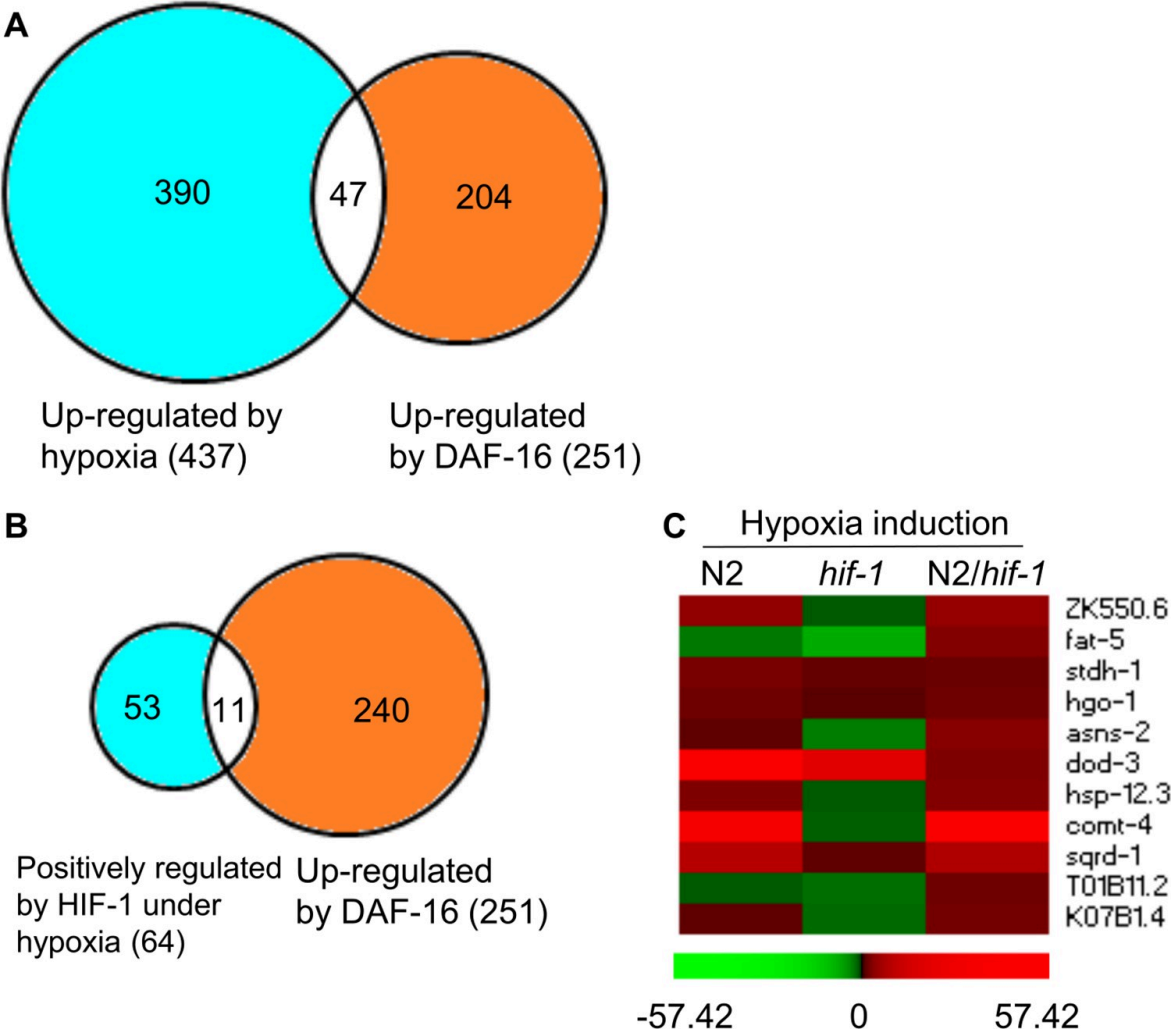
Genes regulated by HIF-1 and hypoxia overlap with genes regulated by DAF-16. Genes identified in this study as up-regulated by hypoxia overlap with genes previously shown to be up-regulated by DAF-16 [30] (*p*-value = 4.22E-29, by Fisher’s exact test). (B) Eleven genes shown in this study to be positively regulated by HIF-1 under hypoxia were also shown by others [30] to be up-regulated by DAF-16 (*p*-value = 6.80E-10, by Fisher’s exact test). (C) The heat map shows the hypoxia-induced changes in gene expression for these 11 genes in N2 and *hif-1(ia04)*, and the relative inductions (N2/*hif-1(ia04)*). The numeric values are in S6 Table. Values < 0 are green, values > 0 are red. The color intensities correspond to the induction levels.

We also asked whether our dataset overlapped significantly with a list of genes shown to be regulated by DAF-16. DAF-16 is an important transcriptional regulator for metabolism, stress response and aging in *C*. *elegans* [29]. Prior studies have identified 251 genes that were up-regulated by DAF-16, and 242 genes for which DAF-16 was a negative regulator [30]. Forty-seven of the identified DAF-16 targets are among the 437 genes identified herein as up-regulated by hypoxia (Fig 3A and S10 Table) [30]. The overlap is significant (*p*-value = 4.22E-29, by Fisher’s exact test). Genes up-regulated by hypoxia and DAF-16 are involved in multiple biological processes, including lipid metabolism (*acs-2*, ZK550.6, *far-3* and *stdh-1*), amino acid metabolism (*hgo-1*), stress response (*cyp-34A9*, *dod-*3, *mtl-1*, *ugt-41*, *hsp-12*.*3*, *hsp-16*.*2*, *sodh-1* and *sqrd-1*), and cellular signaling (*comt-*3 and *comt-4*) (S10 Table). And among the 64 genes positively regulated by HIF-1 under hypoxia, 11 genes have been shown to be up-regulated by DAF-16 (Fig 3B; *p*-value for overlap = 6.80E-10, by Fisher’s exact test). These 11 genes are involved in a variety of biological processes, including lipid metabolism (ZK550.6, *fat-5* and *stdh-1*), amino acid metabolism (*hgo-1* and *asns-2*), stress response (*dod-3*, *hsp-12*.*3* and *sqrd-1*), and cellular signaling (*comt-4*). The heat map in Fig 3C shows the hypoxia-responsive changes in gene expression for these 11 genes in N2 and *hif-1(ia04)*, and the relative inductions (N2/*hif-1(ia04)*).

### Effects of HIF-1-dependent hypoxia-responsive genes on hypoxia adaptation

Since *hif-1*-deficient mutants have a decreased ability to survive hypoxia, we expected that some of the genes regulated by HIF-1 might also have essential roles in hypoxia adaptation. We examined the requirement for genes that were induced by hypoxia in a *hif-1*-dependent manner, with a focus on genes that had not been tested for their effects on hypoxia survival or had not been shown to have essential roles in development in normoxia. Twenty-seven mutants or RNAi treatments were examined to test 23 genes, and these genes had functions in multiple biological processes, including lipid metabolism, protein and amino acid metabolism, detoxification and stress response, ion transport, oxygen binding, vitamin biosynthesis, cellular signaling, protein translation regulation and collagen synthesis.

**Fig 4:**
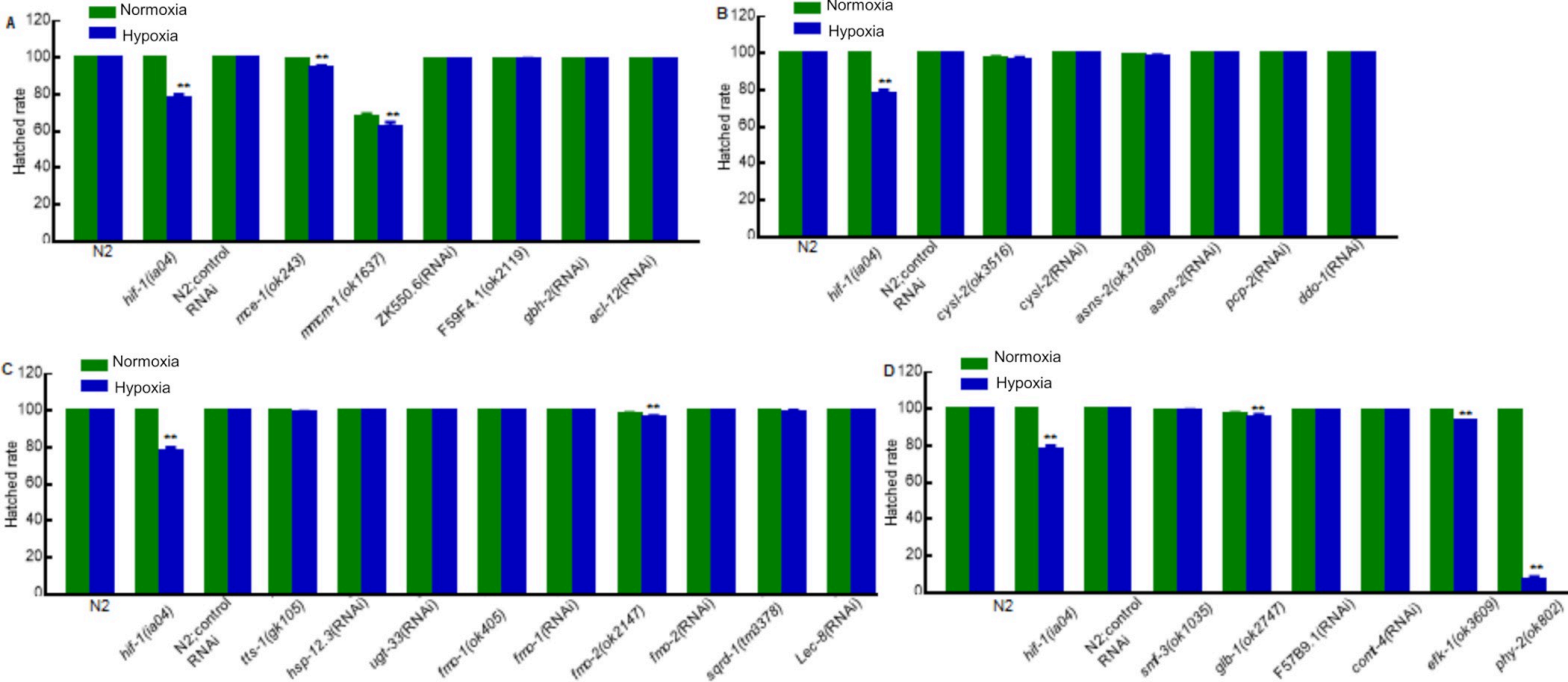
Effects of HIF-1-dependent hypoxia-responsive genes on embryogenesis. (A-D) Hatched rates in normoxia and hypoxia for animals lacking specific gene functions related to (A) lipid metabolism; (B) amino acid metabolism; (C) detoxification and stress response; (D) ion transport (*smf-3*), oxygen binding (*glb-1*), vitamin biosynthesis (F57B9.1), cellular signaling (*comt-4*), protein translation regulation (*efk-1*) and collagen synthesis (*phy-2*). Values are mean ± SEM calculated from three biological replicates. The total number of animals assayed from three biological replicates for each strain in normoxia or hypoxia ranged from 205 to 661. The specific total numbers of animals assayed for each strain in normoxia or hypoxia are provided in S11 Table. For each genotype, to test the effect of hypoxia on hatching, the binary hatched *vs*. un-hatched data were analyzed by fitting a generalized linear model using a logit link function with JMP 9 statistical software (SAS Institute Inc., Cary, NC, 2010) to generate *p*-value. The replicate (three levels) and the treatment (two levels, hypoxia and normoxia) were used as factors in the model. For situations in which such models were inappropriate, randomization tests were used. **p < 0.01, hypoxia against normoxia for each genotype.

**Fig 5:**
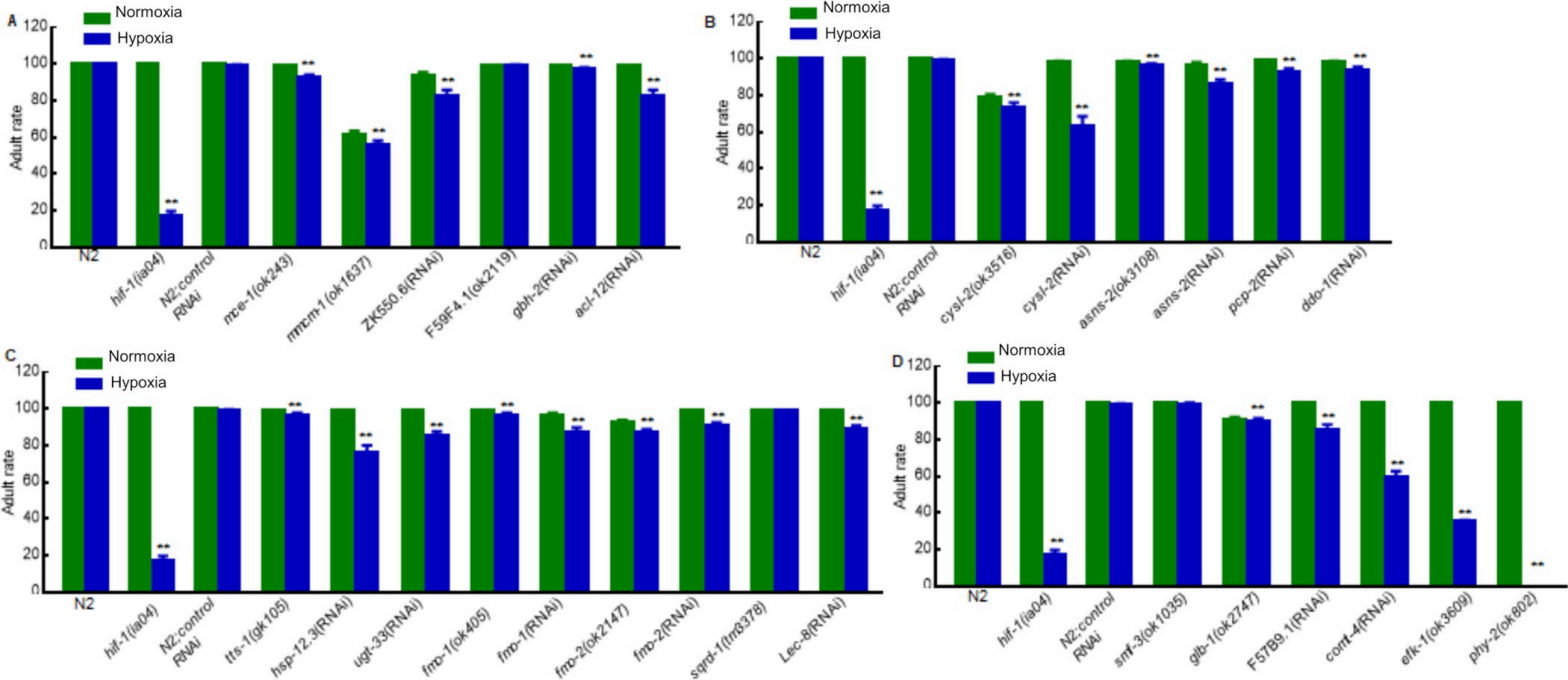
Effects of HIF-1-dependent hypoxia-responsive genes on survival to adulthood. (A-D) Rates of survival to adulthood in normoxia and hypoxia for animals lacking specific gene functions related to (A) lipid metabolism; (B) amino acid metabolism; (C) detoxification and stress response; (D) ion transport (*smf-3*), oxygen binding (*glb-1*), vitamin biosynthesis (F57B9.1), cellular signaling (*comt-4*), protein translation regulation (*efk-1*) and collagen synthesis (*phy-2*). Values were mean ± SEM calculated from three biological replicates. The total number of animals assayed from three biological replicates for each strain in normoxia or hypoxia ranged from 205 to 661. The specific total numbers of animals assayed for each strain in normoxia or hypoxia are provided in S11 Table. For each genotype, to test the effect of hypoxia on adulthood, the binary adult *vs*. non adult data were analyzed by fitting a generalized linear model using a logit link function with JMP 9 statistical software (SAS Institute Inc., Cary, NC, 2010) to generate *p*-value. The replicate (three levels) and the treatment (two levels, hypoxia and normoxia) were used as factors in the model. For situations in which such models were inappropriate, randomization tests were used. **p < 0.01, hypoxia against normoxia for each genotype.

To assay the effects of a particular gene on hypoxia development and survival, we compared the abilities of animals to survive embryogenesis and larval development in hypoxia (0.5% oxygen) *versus* normoxia. These data are illustrated in Figs 4 and 5 (see also S11 Table). Wild-type N2 or N2 fed control RNAi (L4440 empty vector) and *hif-1(ia04)* mutants were used as controls. As expected, N2 and N2 fed control RNAi were tolerant to hypoxia: their survival rates did not decrease under hypoxia compared to normoxia (*p*-values > 0.05). By contrast, *hif-1(ia04)* mutants were sensitive to hypoxia: only 78% hatched and 18% survived to adulthood in hypoxic conditions (**p-values < 0.01) (Figs 4 and 5 and S11 Table).

Among the 27 mutants or RNAi conditions tested, 7 exhibited decreases in embryonic viability under hypoxia compared to normoxia, and 23 were less able to survive to adulthood (**p-values < 0.01). Some mutant animals could complete embryogenesis and hatch under hypoxia, but they could not survive to adulthood in 0.5% oxygen conditions. In agreement with prior studies that have investigated other genes downstream of HIF-1 [17,18], the *hif-1* mutant control exhibited a more severe phenotype than most of the downstream targets tested. Notably, mutations in *hsp-12*.*3*, *cysl-2*, *comt-4*, *efk-1* or *phy-2* strongly impacted hypoxia survival, reducing survival to adulthood 23%, 34%, 41%, 64% and 100%, respectively, under hypoxia compared to normoxia.

### Identifying the direct targets of HIF-1 by ChIP-seq

**Fig 6:**
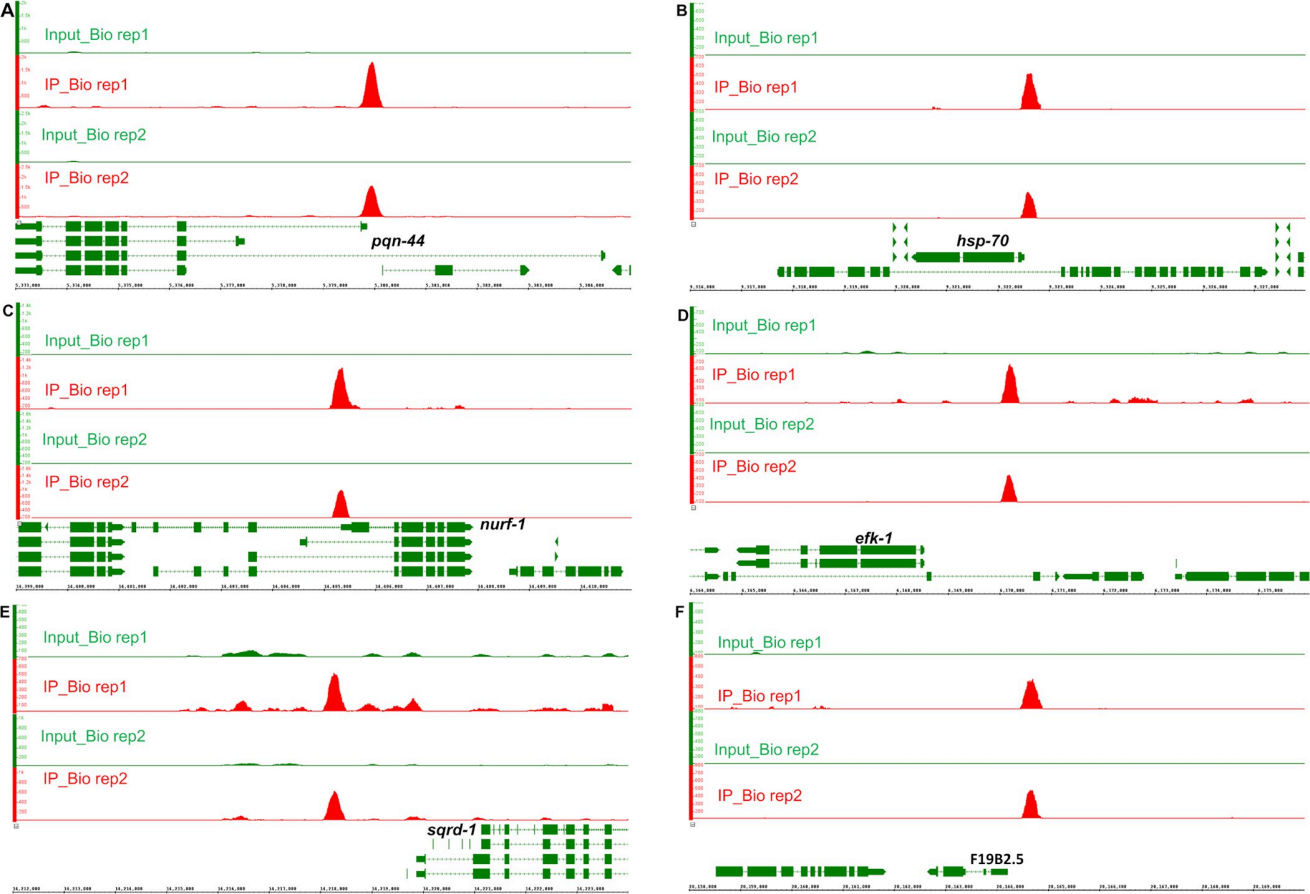
HIF-1 binding regions near six genes regulated by hypoxia or HIF-1. (A) A 600-bp HIF-1 binding region (chrI:5379600–5380199) within *pqn-44* (chrI:5372851–5384508). (B) A 400-bp HIF-1 binding region (chrI:9322400–9322799) upstream of *hsp-70* (chrI:9320325–9322519) and overlap with 5’ UTR. (C) A 600-bp HIF-1 binding region (chrII:14405000–14405599) within *nurf-1* (chrII:14390713–14407911). (D) A 400-bp HIF-1 binding region (chrIII:6170000–6170399) 1453-bp region upstream of the transcription start of *efk-1* (chrIII:6164906–6168547). (E) A 600-bp HIF-1 binding region (chrIV:14218000–14218599) that is 1272 bp upstream of the transcription start of *sqrd-1* (chrIV:14219871–14224440). (F) A 600-bp HIF-1 binding region (chrV:20164400–20164999) that is 235 bp upstream of the transcription start of F19B2.5 (chrV:20162615–20164165). The images show the IGB ChIP-seq signals from both biological replicates. For each binding region in each biological replicate, the minimum number for the y-axis scale was the normalized average input tag count, and the maximum number was the averaged ChIP tag count, as provided in S12 Table.

**Fig 7:**
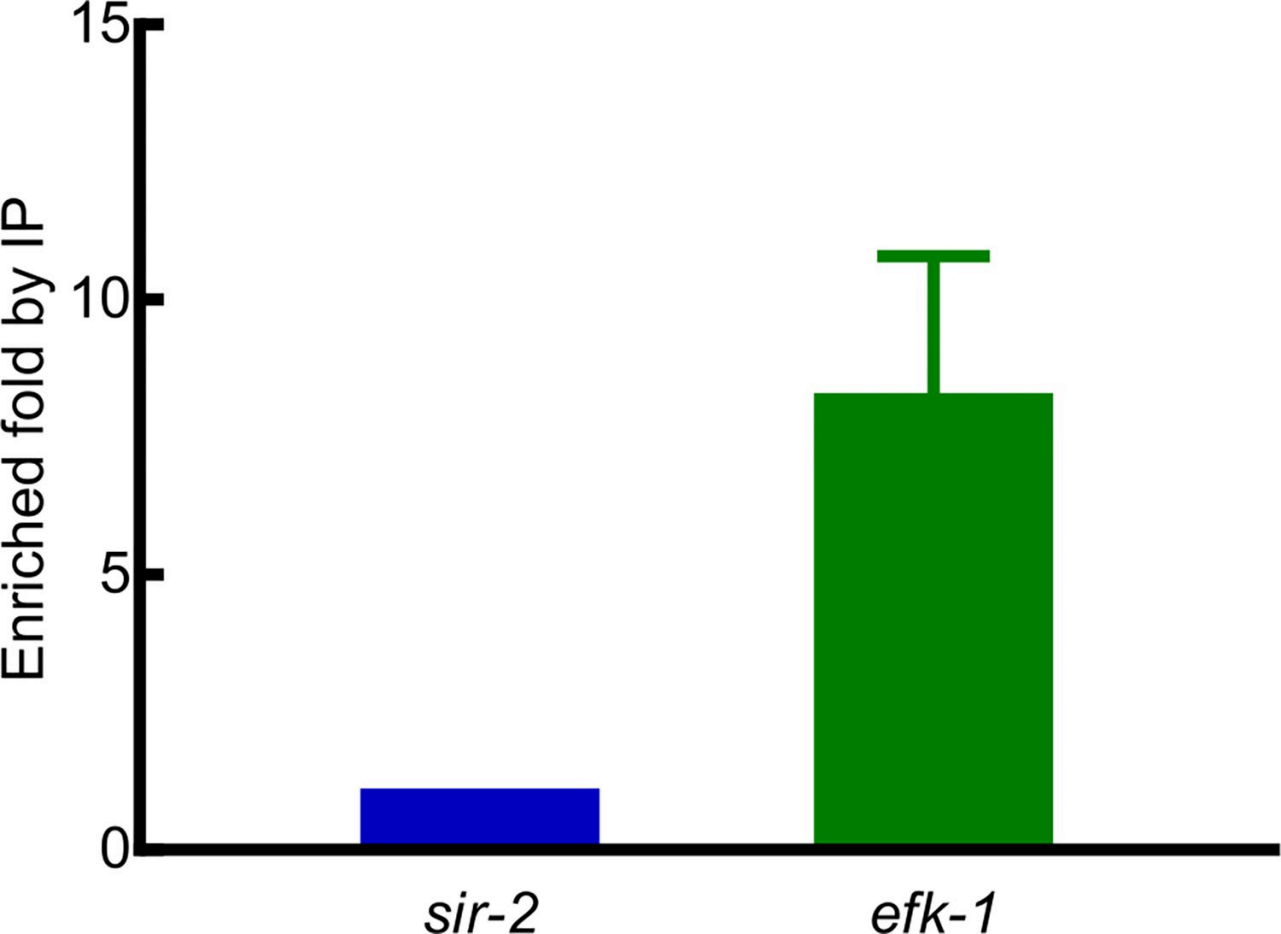
*efk-1* promoter region co-immunoprecipitation with HIF-1. ChIP-qPCR to verify the HIF-1 binding site in the *efk-1* promoter region. The *sir-2* promoter region was used as the reference. *sir-2* is not regulated by HIF-1, and the *sir-2* ChIP-qPCR amplicon contains no sequences similar to HIF-1 binding sites. *efk-1* promoter region ChIP-qPCR was normalized first to input and then to *sir-2* promoter region to obtain the relative enrichment. The bar shows the average *efk-1* promoter region enrichment from three biological replicates. Error bar is SEM.

To identify the genome sequences bound by HIF-1, we performed co-immunoprecipitation experiments. While there are six splicing isoforms of *hif-1*, the isoform a (*hif-1a*) had been shown to be essential for longevity and stress resistance [24,31,32]. Accordingly, we identified DNA sequences that co-immunoprecipitated with an epitope-tagged version of this HIF-1 isoform. We identified 94 HIF-1 binding peaks (FDR ≤ 0.05 and fold enrichment ≥ 1.6) that were reproducible in two biological replicates. The summaries of these peaks (including peak coordinates, sizes, distributions, ChIP and input tag counts, fold enrichments, gene assignment and related expression data) are provided in S12 Table. Sequences co-immunoprecipitated with HIF-1 were provided in S1–S6 Files, organized by chromosomes and peak coordinates, one chromosome one file. ChIP signals were visually verified in IGB (Integrated Genome Browser) and are provided in S1–S6 Figs, organized by chromosomes and target genes, one chromosome one file. The IGB ChIP signals for *pqn-44* (prion-like-(Q/N-rich)-domain-bearing protein), *hsp-70* (heat shock protein), *nurf-*1 (nucleosome remodeling factor complex homolog), *efk-1* (eukaryotic elongation factor 2 kinase), *sqrd-1* (sulfide quinone oxidoreductase) and F19B2.5 (SNF2_N domain-containing protein) are presented in Fig 6. These six genes were identified by both expression studies and co-immunoprecipitation analyses as HIF-1 direct targets (see Table 6). HIF-1 bound at different locations relative to these genes. The HIF-1 binding regions were within the coding regions of *pqn-44* and *nurf-1*. The HIF-1 binding site near *hsp-70* was upstream of the transcription start sites and overlapped with 5’ UTRs. The HIF-1 binding sites for *efk-1*, *sqrd-1* and F19B2.5 were also upstream of the transcription start sites (Fig 6). Among the HIF-1 direct targets, *efk-1* was of particular interest. *efk-1* expression was induced by hypoxia in a *hif-1*-dependent manner (Table 3), and as shown in Figs 4 and 5, animals lacking *efk-1* function were less able to survive hypoxic treatments. We verified the HIF-1 binding region in the *efk-1* promoter by ChIP-qPCR. The enrichment of this region was 8-fold relative to the reference *sir-2* promoter region (Fig 7). Consistent with this finding, this region was also identified as a HIF-1 binding site by the ModERN project [33].

**Fig 8:**
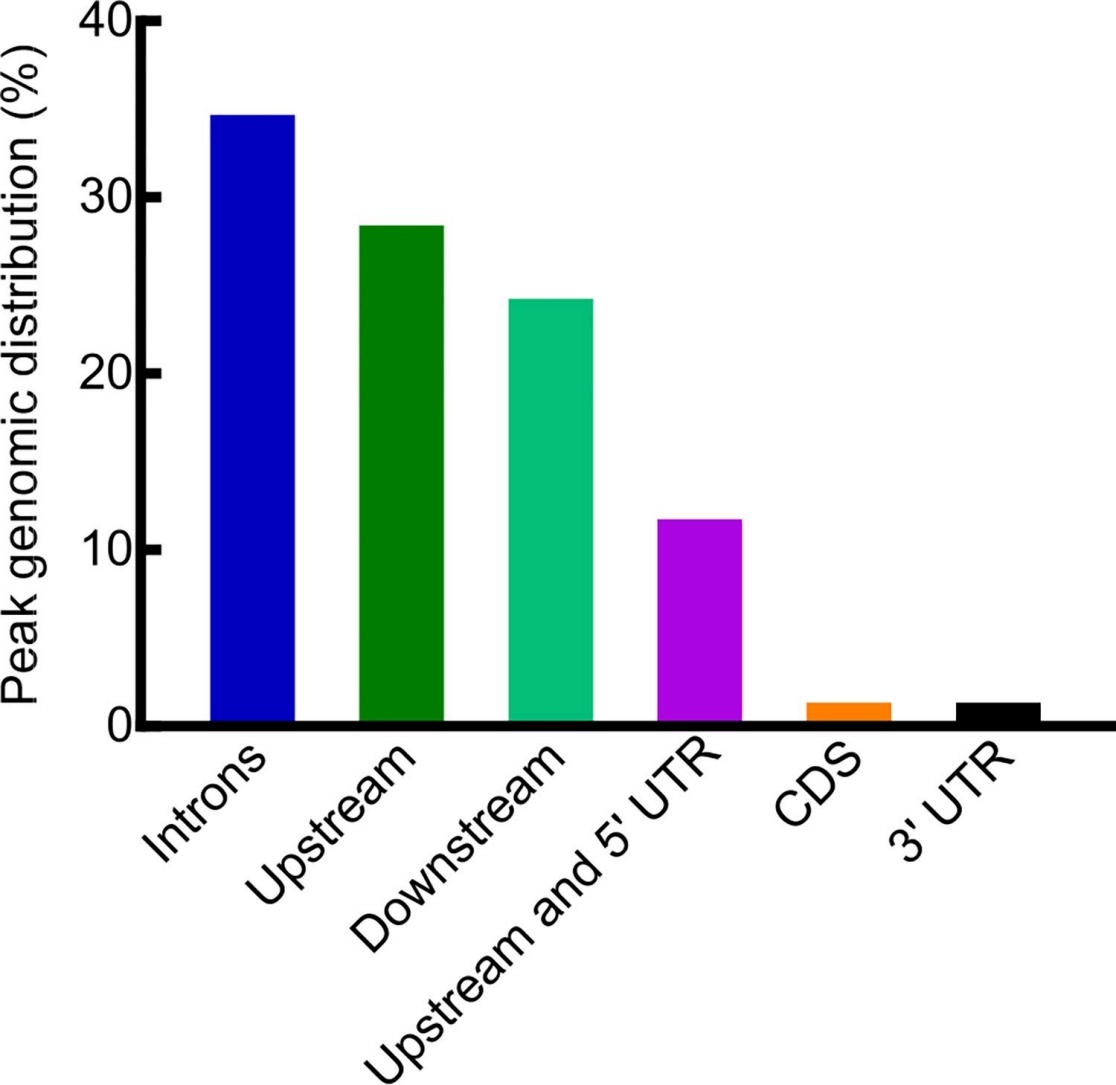
Genomic distributions of HIF-1 ChIP peaks. Genomic distributions of HIF-1 ChIP peaks relative to target genes. The detailed distributions for each peak were provided in S12 Table.

Most of the HIF-1 binding peaks were located in introns (34.38%), upstream of the transcription start sites (28.13%), or downstream of the transcription stop sites (23.96%). The rest of binding peaks lied upstream of the transcription start sites and overlap with 5’ UTRs (11.46%), or in the coding regions (1.04%), as well as in 3’ UTRs (1.04%) (Fig 8 and S12 Table). A majority of the HIF-1 binding peaks (60/94 = 64%) contained sequences similar to mammalian HRE (hypoxia response element) 5’-RCGTG-3’ (R = A or G), or the mandatory core HRE 5’-CGTG-3’ [34] (S12 Table and S1–S6 Files).

The 94 HIF-1 binding regions that we identified through chromatin immunoprecipitation were proximal to 96 genes (S12 Table), which we describe here as HIF-1 direct target genes. These genes are enriched for the functional category of stress response (Cat 1 in Tables 5 and S13), and the major subcategory is heat stress response, including the heat shock protein genes *hsp-16*.*2*, *hsp-16*.*41*, *hsp-70*, *hsp-90* and *hsp-110*. Other direct targets with stress response roles include immune response genes (*tir-1*, *cpr-3* and *irg-1*), *sqrd-1* (sulfide:quinone reductase), and the *ero-1* endoplasmic reticulum oxidase involved in unfolded protein response.

**Table 5:** Enriched biological terms associated with HIF-1 direct target genes.

Biological term	Count	Bonferroni FDR	Genes
Cat1: Stress response	15	4.44E-06	*cpr-3*, *hsp-90*, *daf-25*, *ero-1*, *hsp-16*.*2*, *hsp-16*.*41*, *hsp-70*, *tir-1*, *sqrd-1*, F44E5.4, F44E5.5, Y105E8B.9, *irg-1*, *hsp-110*, K01A2.5
Cat2: Stress response: heat	7	1.17E-09	*hsp-90*, *hsp-16*.*2*, *hsp-16*.*41*, *hsp-70*, F44E5.4, F44E5.5, *hsp-110*

### Genomic regions that co-immunoprecipitate with HIF-1 map to genes that are regulated by HIF-1

**Fig 9:**
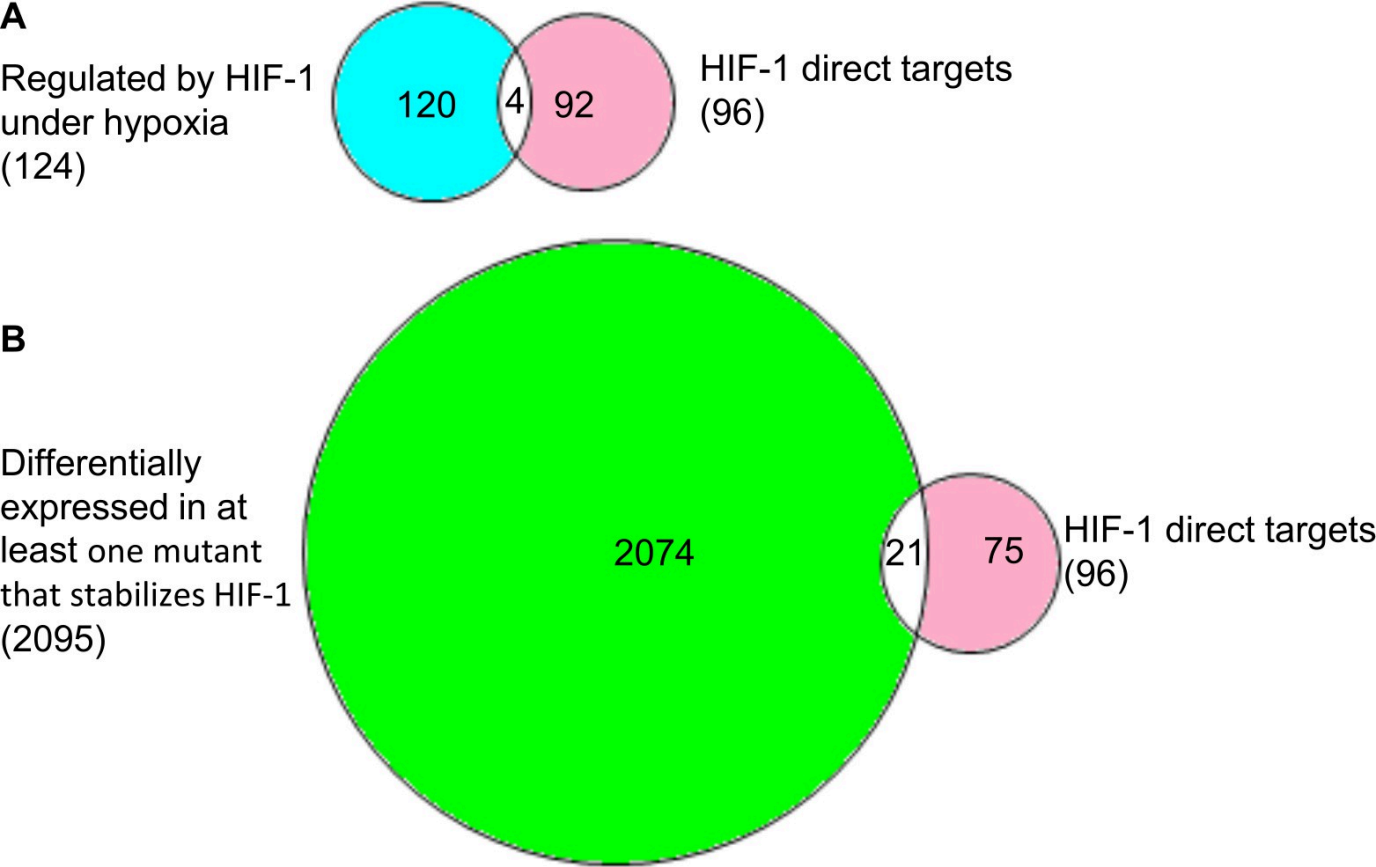
Overlaps between HIF-1 direct targets and genes regulated by HIF-1. (A) Illustration of the overlap between HIF-1 direct targets and genes identified herein as regulated by HIF-1 in response to hypoxia. (B) The overlap between HIF-1 direct targets and genes differentially expressed in at least one mutant that stabilizes HIF-1.

Among the 96 gene regions that co-immunoprecipitated with HIF-1, 23 mapped to genes that that we have shown to be regulated by HIF-1. Some of the 96 genes were regulated by hypoxia in a *hif-1*-dependent manner, and others were shown to be regulated in mutants with constitutively active HIF-1 [*vhl-1(ok161)*, *rhy-1(ok1402)* and *egl-9(sa307)*, and *swan-1(ok267);vhl-1(ok161)* double mutants] (Table 6). The Venn diagrams in Fig 9 illustrate these overlaps.

**Table 6:** Genes that were regulated by HIF-1 and identified as direct targets through chromatin immunoprecipitation.

Expression	Count	Genes
Positively regulated by HIF-1 under short-term hypoxia	3	*efk-1*, *sqrd-1*, W03F9.1
Negatively regulated by HIF-1 under short-term hypoxia	1	*tir-1*
Up-regulated in at least one mutant that stabilizes HIF-1	13	Y105E8B.9, F44E5.5, F44E5.4, *nurf-1*, *efk-1*, *sqrd-1*, *hsp-16*.*41*, *hsp-16*.*2*, *irg-*1, *ari-1*.*4/tag-349*, F54D5.12/ *dhgd-1*, *cpr-3*, *hsp-70*
Down-regulated in at least one mutant that stabilizes HIF-1	8	C50A2.3, F54D5.12/*dhgd-1*, *oat-1*, Y105C5B.5, F19B2.5, M04F3.3*/kin-35*, Y94H6A.5, C23H5.8

An interesting feature emerging from the expression patterns of HIF-1 direct targets is that they responded to HIF-1 in differing contexts. Some genes responded to HIF-1 under short-term hypoxia (for example, W03F9.1 and *tir-*1), while others were differentially expressed in mutants in which HIF-1 was constitutively active (for example, *oat-1* and *cpr-3*). As noted in Table 6, some of the genes that co-immunoprecipitated with HIF-1 had been shown to respond to HIF-1 under both short-term hypoxia and in the mutants with constitutively active HIF-1 (like *efk-1* and *sqrd-1*).

## Discussion

Transcription factors HIFs are the master regulators of hypoxia response. Identification of direct HIF-1 targets is a major step towards more fully understanding the transcriptional networks controlled by *C*. *elegans* HIF-1. Here, we describe hypoxia-responsive gene expression, in ways that provide new insights to HIF-1 mediated hypoxia response. By cross-referencing the genes that are differentially regulated by hypoxia or HIF-1 with those genomic regions that co-immunoprecipitate with HIF-1, we report the direct and downstream targets of HIF-1 with greater confidence.

### Refined understanding of HIF-1 mediated short-term hypoxia response

A prior microarray experiment provided a foundation for understanding HIF-1 function. In that experiment, L3-stage worms were treated with 0.1% oxygen for 4 hours, and 63 genes were identified as HIF-1-dependent hypoxia-responsive genes [17]. However, subsequent studies showed that HIF-1 activity reporter *Pnhr-57*::*GFP’*s levels actually peaked after 2 hours of hypoxia treatment rather than 4 hours of hypoxia treatment (0.5% oxygen) [35]. In this study, we examined the genome-wide gene expression changes after 2 hours of 0.5% oxygen treatment in L4-stage worms. By comparing the hypoxia responses in N2 and *hif-1*-deficient animals, we identified 124 genes as being regulated by hypoxia in a *hif-1*-dependent manner. Reassuringly, there was some overlap with genes that had been identified in previous studies [17] as responsive to a slightly longer hypoxia treatment (4 hours), including the genes F22B5.4, *egl-9*, *phy-2*, *fmo-12/fmo-2*, K10H10.2/*cysl-2*, *efk-1*, F57B9.1 and *dod-3*.

We hypothesized that some of the genes that were responsive to hypoxia in a *hif-1*-dependent manner would be essential for survival in hypoxic conditions. Indeed, the experiments illustrated in Figs 4 and 5 showed that most of the mutants or RNAi treatments tested did reduce embryonic or larval survival in hypoxia. We note that *phy-2*-deficient animals were especially sensitive to hypoxia, as might be expected for an essential prolyl 4-hydroxylase enzyme involved in collagen synthesis. Interestingly, mammalian HIF-1α has also been shown to regulate collagen synthesis, and misregulation of HIF-1 was shown to impair the development of cartilage [36]. In future studies, it might also be of interest to examine the functional roles of other hypoxia-responsive genes, beyond the 23 genes analyzed here (in Figs 4 and 5). It is likely that different hypoxia regimens might identify new functions. For example, some genes might be essential for completing specific embryonic or larval stages in low oxygen conditions. Interestingly, *sqrd-1* mutants were able to survive hypoxia in these assays. The *sqrd-1* mitochondrial sulfide quinone oxidoreductase gene has been shown to have an important role in detoxifying hydrogen sulfide, and survival in H_2_S requires both *sqrd-1* and *hif-1* function [27]. The data presented here, when considered with prior studies, suggest that *sqrd-1* acts downstream of HIF-1 and that s*qrd-1* function is more critical to H_2_S detoxification than it is to hypoxia survival. We note that some RNAi treatments did not reveal any roles for the affected genes in hypoxia survival. There are multiple possible explanations for this. Some genes may have nonessential functions, or they may act redundantly with other genes to enable hypoxia survival. In some cases, RNAi may not have fully knocked out gene function.

Our data also raise interesting questions for future studies. For example, it is clear that HIF-1 and DAF-16 both have distinct and overlapping roles in *C*. *elegans* stress responses, and there is still much to be learned about how these two important pathways complement each other. There are also many outstanding questions about how the genes that act downstream of HIF-1 protect *C*. *elegans* from hypoxic stress. Clearly, organisms need to adapt metabolic networks to manage energy needs and to assure proteostasis in reduced oxygen conditions.

### Insights from HIF-1 ChIP-seq

In this study, by performing ChIP-seq, we are able to identify HIF-1 direct targets at a whole genome level. Although the searching for cis-acting regulatory elements is usually focused on the proximal promoter region: 1–2 kb upstream of the start codon, we identified HIF-1 binding sites associated with proximal promoters as well as with enhancers in other locations including introns, coding regions and UTRs (S12 Table, S1–S6 Figs). Our finding that some HIF-1 binding sites are located in introns is in alignment with the emerging theme that introns can contain enhancer elements [37,38]. Among the 94 binding sites for HIF-1, 24 sites (24/94 = 25.53%) were also identified as HIF-1 binding sites by the ModERN project or Vora et al. [21,33]. In this study, we cross-reference HIF-1 chromatin immunoprecipitation data with microarray studies that identify genes regulated by HIF-1 in hypoxia or genes differentially expressed in mutants that up-regulate HIF-1. As might be expected for a regulatory transcription factor, the roles of HIF-1 appear to be context specific. HIF-1 is required for a suite of gene expression changes in response to short-term hypoxia, and these hypoxia-induced genes overlap to some extent with the genes that are misregulated in *vhl-1* or *egl-9* mutants that stabilize HIF-1. There are also gene expression changes specific to one condition or the other. Interestingly, HIF-1 has also been found to have roles in responses to heat, H_2_S and pathogen infection [27,32,39–42]. Some of the genes shown here to co-immunoprecipitate with HIF-1 might have roles in these or other environmental stresses.

## Materials and methods

### Strains

The wild-type *C*. *elegans* used in this study was N2 Bristol. The mutant strains used in this study were listed in S10 Table. All the worms were maintained at 21°C using the standard methods [43].

### Gene expression microarray experiment

Randomized complete block design was followed for the microarray experiment, with three biological replicates treated as three blocks. Each block included eight treatments: N2 wild type, N2 wild type with hypoxia treatment, *hif-1*(*ia04)* loss-of-function mutants, *hif-1*(*ia04)* loss-of-function mutants with hypoxia treatment, *vhl-1(ok161)* loss-of-function mutants, *rhy-1(ok1402)* loss-of-function mutants, *egl-9(sa307)* loss-of-function mutants and *swan-1(ok267);vhl-1(ok161)* loss-of-function double mutants. For each treatment, about 1,000 synchronized L4-stage larvae were pooled as one experimental unit to get sufficient RNA for hybridization. Total RNA isolation was performed using Trizol (Invitrogen) and RNeasy Mini Kit (Qiagen). RNA quality was checked with an Agilent 2100 BioAnalyzer (Agilent Technologies). The RNA integrity numbers (RINs) for all the samples used in this study were greater than 9.0. The total RNA isolated from one experimental unit was hybridized onto one Affymetrix GeneChip® C. elegans Genome array (Affymetrix, part number 900383). Probe synthesis, labeling, hybridization, washing, staining and scanning were performed by the GeneChip facility at Iowa State University. In brief, the total RNA was synthesized to biotin-labeled aRNA using the GeneChip® 3’ IVT Express Kit (Affymetrix, part number 901229) and hybridized to the array. The arrays were washed and stained in the GeneChip® fluidics station 450 and scanned with GeneChip® scanner 3000 7G. The Affymetrix® GeneChip® Command Console™ (AGCC) software was used to generate probe cell intensity data (.CEL) files. The resulting CEL files were normalized and summarized using the robust multichip average (RMA) algorithm [44] in R package (R Core Team, Vienna, Austria, 2016). An analysis of variance (ANOVA) model was then fitted to the summarized expression measures, with the block (three levels) and the treatment (eight levels) treated as fixed effect factors following the experimental design. Residual model diagnostics identified no severe violations of the model assumptions. Linear contrasts of treatment means were tested using the general F-test. To account for multiplicities of hypothesis testing, conservative estimates of false discovery rates (FDRs) were calculated according to the *q*-value procedure of Storey and Tibshirani [45]. Differentially expressed probesets were defined as *q*-value ≤ 0.05 and fold change ≥ 1.6. Probesets were converted to genes using the Affymetrix annotation file “Celegans.na36.annot.csv”. To deal with redundancy and count the number of unique genes detected on the array, we kept one probeset per gene and one gene per probeset. In this way, the total number of unique genes detected on the array was 18, 011. For the purpose of reference, the original complete lists of gene(s) annotated to each probeset were kept in S1-S3, S6 and S7. The complete analysis results for all the conditions [N2 wild type, N2 wild type with hypoxia treatment, *hif-1*(*ia04)* loss-of-function mutants, *hif-1*(*ia04)* loss-of-function mutants with hypoxia treatment, *vhl-1(ok161)* loss-of-function mutants, *rhy-1(ok1402)* loss-of-function mutants, *egl-9(sa307)* loss-of-function mutants and *swan-1(ok267);vhl-1(ok161)* loss-of-function double mutants] and all the probesets on the microarray were provided in S1 Table. This manuscript describes gene expression changes in N2 wild type animals and *hif-1(ia04)* mutants under hypoxia and nomoxia. Genes expression changes in *vhl-1(ok161)*, *egl-9(sa307)*, *rhy-1(ok1402)* and *swan-1(ok267);vhl-1(ok161)* double mutants have been described in a related study [26]. The microarray raw and probeset summary data had been deposited to NCBI’s Gene Expression Omnibus, the accession number is GSE228851.

### Gene function annotation and enrichment analyses

The WormCat online tool (www.wormcat.com) [19] was used to annotate the enriched biological terms associated with microarray and ChIP-seq-selected genes. The enriched biological terms were at Bonferroni false discovery rate cut off of 0.01.

### Heat maps

Heat maps for gene expression profiles were generated by the PermutMatrix graphical analysis program [46,47]. Average linkage clustering was performed using the hypoxia induction values. Green color represents negative values, and red color represents positive values. The intensities of the colors represent the magnitudes of fold changes. Other parameters were set as default.

### Gene lists overlap testing

Fisher’s exact test was performed to test whether the overlap between two gene lists was significant or not. The total number of 18, 011 genes detected on the microarray was used as the population size. The significant overlap is at *p*-value < 0.001.

### HIF-1 chromatin immunoprecipitation sequencing (ChIP-seq)

The ChIP experiments were performed in the *egl-9(sa307)* loss-of-function mutant background to maintain HIF-1 stability and activity in normoxia. The strain used for the ChIP experiments was ZG434 [*egl-9(sa307);iaIS28[Phif-1*::*hif-1a*::*Myc*::*HA];hif-1(ia04)*]. To use the commercially available ChIP grade anti-HA antibody (Abcam, cat. no. ab9110), an HA-tagged *hif-1a* transgene *iaIS28[Phif-1*::*hif-1a*::*Myc*::*HA]* [31] was introduced into *egl-9(sa307)*, and the endogenous *hif-1* gene was knocked out. The detailed ChIP protocol is provided in S7 File. Briefly, synchronized L4-stage worms for each biological replicate were harvested in separate batches at separate times. Harvesting enough synchronized worms for HIF-1 ChIP experiments was laborious due to the egg-laying defect inherent to the *egl-9(sa307)* loss-of-function mutation. For each batch, about 10, 000 L4-stage worms were harvested and cross-linked in 2% formaldehyde at 21°C for 30 minutes. The ChIP-seq experiment was performed with two biological replicates. For each biological replicate, nuclear lysates from about 50,000 worms (pooled from 5 batches of worm collection) were sonicated using a Branson sonifer microtip to fragment the chromatin to 200–800 bp. Immunoprecipitated protein-DNA complexes were captured on protein A-Sepharose beads (Sigma) and eluted in elution buffer (1% SDS and 100 mM NaHCO3) at 65°C for 30 minutes. Following RNase treatment and cross-link reversal, the ChIP DNA was purified with the Qiagen MinElute Kit and stored at -20°C for sequencing in parallel with the corresponding input DNA. The ChIP-Seq library preparation and sequencing were performed by the Iowa State University DNA facility. In brief, NEXTflex™ ChIP-Seq Barcodes kit (Illumina compatible) (BIOO Scientific Corp., cat. no. 514123) was used to prepare multiplexed single-end genomic DNA libraries. The gel slices corresponding to the 200–300 bp maker were cut and purified. The purified DNA was amplified and sequenced in a single flow cell on the IlluminaHiSeq 2000 platform. The length of reads was 50 bp.

### HIF-1 ChIP-seq data analyses

The HIF-1 ChIP-seq raw and processed data has been deposited to NCBI’s Gene Expression Omnibus and the accession number is GSE228846. The quality scores of the fastq reads for the input and ChIP DNA samples from both biological replicates were all above 30, indicating high quality sequencing data (S7 Fig). Reads were mapped to *C*. *elegans* reference genome ce11 using bowtie 2 with the default settings. Reads with mapping quality (MAPQ) score less than 10 or reads mapped to the mitochondrial genome were excluded. At the end, reads kept for peak calling were 19–43 million per sample. The kept reads were assigned to bins, the size of which was set at 200 bp to match the fragment length for Illumina sequencing. Bin-level read counts were analyzed by the R package MOSAiCS (MOdel-based one and two Sample Analysis and Inference for ChIP-Seq Data) to call peaks [48] (https://www.bioconductor.org/packages/release/bioc/manuals/mosaics/man/mosaics.pdf). The false discovery rate (FDR) was set at 0.05. Neighboring peaks were merged. The output peaks were further filtered with the following criteria: (1) minimum posterior probability ≤ 0.05; (2) averaged input tag count ≥ 10; (3) averaged ChIP tag count ≥ 10; and (4) fold enrichment (averaged ChIP tag count/normalized average input tag count) ≥ 1.6. The identified peaks were visually verified in IGB (Integrated Genome Browser) [49]. The WIG files for IGB were provided in GEO database (accession number GSE228846). Peaks identified by both biological replicates were treated as HIF-1 binding regions. Peaks were assigned to genes within 6 kb. Within this region, if there was a gene(s) differentially expressed under hypoxia or in the HIF-1 negative regulator mutants (*vhl-1(ok161)*, *egl-9(sa307)*, *rhy-1(ok1402)* and *swan-1(ok267);vhl-1(ok161)* mutants), the peak was assigned to this gene. We reasoned that a gene(s) showed expression change under these conditions was more likely to be a HIF-1 direct target than genes showed no expression changes. Otherwise the nearest gene was assigned to the peak. Most often (90 out of 96 genes), the assigned HIF-1 direct target was the nearest gene.

### ChIP-qPCR to verify the HIF-1 binding site in the *efk-1* promoter

The primers for ChIP-qPCR to verify the HIF-1 binding region in the *efk-1* promoter were the forward primer 5’-CAATCTGACCGAGCCGAATG-3’ and reverse primer 5’-AGGCCTTTCTCGATTTTCCA-3’. The amplicon was 172 bp and contained a HRE 5’-ACGTG-3’. The promoter region of *sir-2*, a gene not regulated by HIF-1 under short-term hypoxia or in the HIF-1 negative regulator mutants, was used as the reference. The primers for *sir-2* ChIP-qPCR were the forward primer 5’-AGATTGCTTCTTTGGCTGGA-3’ and reverse primer 5’-GTAACGCACCTTGCAACAGA-3’. The amplicon was 218 bp and did not contain HRE similar sequences. Three biological replicates were performed. qPCR quantification was performed using the efficiency-corrected comparative quantification method [50].

### Hypoxia development and survival assays

For each mutant genotype, the normoxia and hypoxia treatments were performed in parallel at 21°C. For each treatment, 20 young adults (one day after L4 molt) were used as parents to lay eggs on one NGM plate seeded with OP50 for 30 minutes. After counting the eggs laid, the plates were kept in normoxia or put into a sealed plexiglass chamber with constant hypoxic gas flow for 24 hours. Compressed air and 100% nitrogen were mixed to achieve 0.5% oxygen, and gas flow was controlled by an oxygen sensor [17]. After 24 hours, the un-hatched eggs were counted for both treatments. After that, the plates for both treatments were maintained in normoxia. The adult worms were counted 72 hours after the eggs had been laid. The data collection time points were set to match the development rate of N2 eggs in normoxia: they hatched within 24 hours and reached adulthood within 72 hours.

For RNAi strains, RNAi was induced by bacterial feeding as described [51,52]. Except for F57B9.1, *gbh-2* and *comt-4*, the RNAi clones were purchased from the Ahringer RNAi library (Geneservice, Cambridge, UK) and validated by sequencing. The RNAi constructs for F57B9.1, *gbh-2* and *comt-4* were generated by cloning the coding regions into the L4440 double-T7 vector [51]. The primers for cloning the F57B9.1 RNAi fragment were the forward primer 5’-TTCAGACATTCGGGCAAAAT-3’ and reverse primer 5’-AAGCCTCTTGAGAAGCCACA-3’; this amplicon was 752 bp, including exons 2, 3, 4 and introns 2 and 3. The primers for cloning the *gbh-2* RNAi fragment were the forward primer 5’-GTTACCGGCTGGAATTTGAA-3’ and reverse primer 5’-TGGGCTTTCGTTTCTCAACT-3’; this amplicon was 1626 bp, including exons 1, 2, 3, 4 and introns 1, 2, 3. And the primers for cloning the *comt-4* RNAi fragment were the forward primer 5’-GCTCCTGAAGTTCTTACATTTGG-3’ and reverse primer 5’-GATTGAGAAGCGCCGAGTAG-3’; this amplicon was 749 bp, including exons 5, 6, 7 and introns 5 and 6. To generate the RNAi parent generation, 20 N2 adults maintained with OP50 were transferred to RNAi plates to lay eggs for 1 hour. Three days later, 20 young adults grown up from these eggs were randomly picked as RNAi parents to lay eggs on a new RNAi plate for 30 minutes for either normoxia or hypoxia treatment. The normoxia and hypoxia treatments were performed in parallel at 21°C. The downstream procedures for hypoxia treatment and counting the hatched/un-hatched eggs and adult/non adult animals were the same as those described above for the mutant strains.

The experiments were performed with three biological replicates. To test the effect of hypoxia on animal development and survival, the binary hatched *vs*. un-hatched or adult *vs*. non adult data were analyzed by fitting a generalized linear model using a logit link function with JMP 9 statistical software (SAS Institute Inc., Cary, NC, 2010). The replicate (three levels) and the treatment (two levels) were used as factors in the model. For situations in which such models were inappropriate, randomization tests were used.

## Supporting information

S1 Fig HIF-1 direct targets ChIP-seq IGB signals on Chromosome 1. (PPTX)

S2 Fig HIF-1 direct targets ChIP-seq IGB signals on Chromosome 2. (PPTX)

S3 Fig HIF-1 direct targets ChIP-seq IGB signals on Chromosome 3. (PPTX)

S4 Fig HIF-1 direct targets ChIP-seq IGB signals on Chromosome 4. (PPTX)

S5 Fig HIF-1 direct targets ChIP-seq IGB signals on Chromosome 5. (PPTX)

S6 Fig HIF-1 direct targets ChIP-seq IGB signals on Chromosome X. (PPTX)

S7 Fig Quality scores of fastq reads for the input and ChIP DNA samples. (PPTX)

S1 Table Gene expression for all the probesets under hypoxia and in the HIF-1 negative regulator mutants. (XLSX)

S2 Table Genes up-regulated by short-term hypoxia in N2. (XLSX)

S3 Table Genes down-regulated by short-term hypoxia in N2. (XLSX)

S4 Table Enriched biological terms for genes up-regulated by short-term hypoxia in N2. (XLSX)

S5 Table Enriched biological terms for genes down-regulated by short-term hypoxia in N2. (XLSX)

S6 Table Genes positively regulated by HIF-1 under short-term hypoxia. (XLSX)

S7 Table Genes negatively regulated by HIF-1 under short-term hypoxia. (XLSX)

S8 Table Enriched biological terms for genes positively regulated by HIF-1 under hypoxia. (XLSX)

S9 Table Enriched biological terms for genes negatively regulated by HIF-1 under hypoxia. (XLSX)

S10 Table Genes upregulated by hypoxia and DAF-16. (XLSX)

S11 Table Effects of HIF-1-dependent hypoxia responsive genes on hypoxia development and survival. (XLSX)

S12 Table Direct targets for HIF-1 identified by ChIP-seq. (XLSX)

S13 Table Enriched biological terms for HIF-1 dirct target genes. (XLSX)

S14 Table Descriptions of mutations used in this study. (DOCX)

S1 File Sequences co-immunoprecipitated with HIF-1 on chromosome 1. (DOCX)

S2 File Sequences co-immunoprecipitated with HIF-1 on chromosome 2. (DOCX)

S3 File Sequences co-immunoprecipitated with HIF-1 on chromosome 3. (DOCX)

S4 File Sequences co-immunoprecipitated with HIF-1 on chromosome 4. (DOCX)

S5 File Sequences co-immunoprecipitated with HIF-1 on chromosome 5. (DOCX)

S6 File Sequences co-immunoprecipitated with HIF-1 on chromosome X. (DOCX)

S7 File Detailed protocol for HIF-1 chromatin immunoprecipitation (ChIP). (DOCX)
