# Supplementary material for: Whole genome profiling of short-term hypoxia induced genes and identification of HIF-1 binding sites provide insights into HIF-1 function in Caenorhabditis elegans
Source: PLoS One. 2024 May 14;19(5):e0295094. doi: 10.1371/journal.pone.0295094 (PMC11093353; doi:10.1371/journal.pone.0295094)
Supplement: S1 Fig — (PPTX) [file pone.0295094.s001.pptx]

## Slide 1
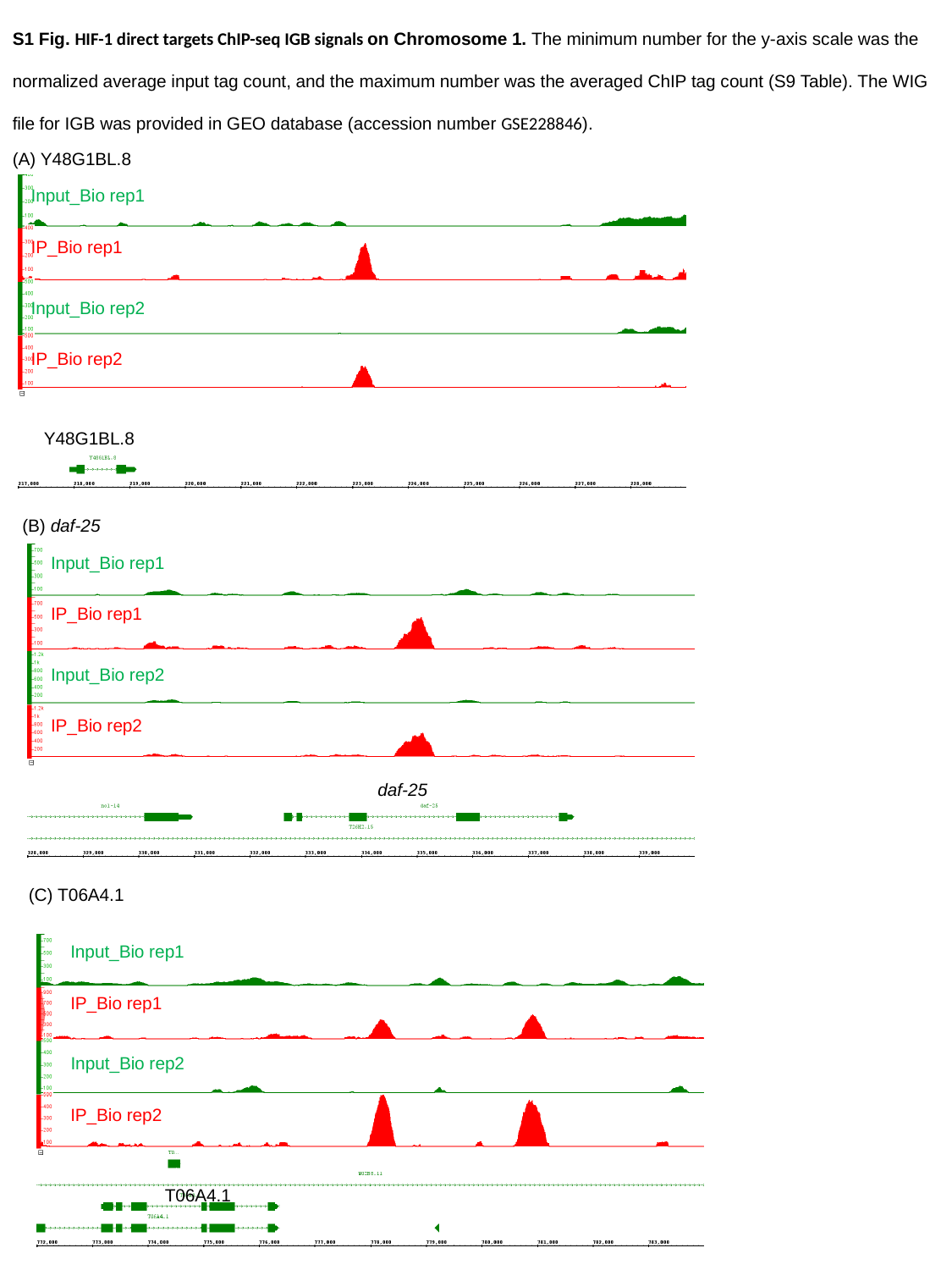

S1 Fig. HIF-1 direct targets ChIP-seq IGB signals on Chromosome 1. The minimum number for the y-axis scale was the normalized average input tag count, and the maximum number was the averaged ChIP tag count (S9 Table). The WIG file for IGB was provided in GEO database (accession number GSE228846).
(A) Y48G1BL.8
Input_Bio rep1
IP_Bio rep1
Input_Bio rep2
IP_Bio rep2
Y48G1BL.8
(B) daf-25
daf-25
Input_Bio rep1
IP_Bio rep1
Input_Bio rep2
IP_Bio rep2
(C) T06A4.1
Input_Bio rep1
IP_Bio rep1
Input_Bio rep2
IP_Bio rep2
T06A4.1

## Slide 2
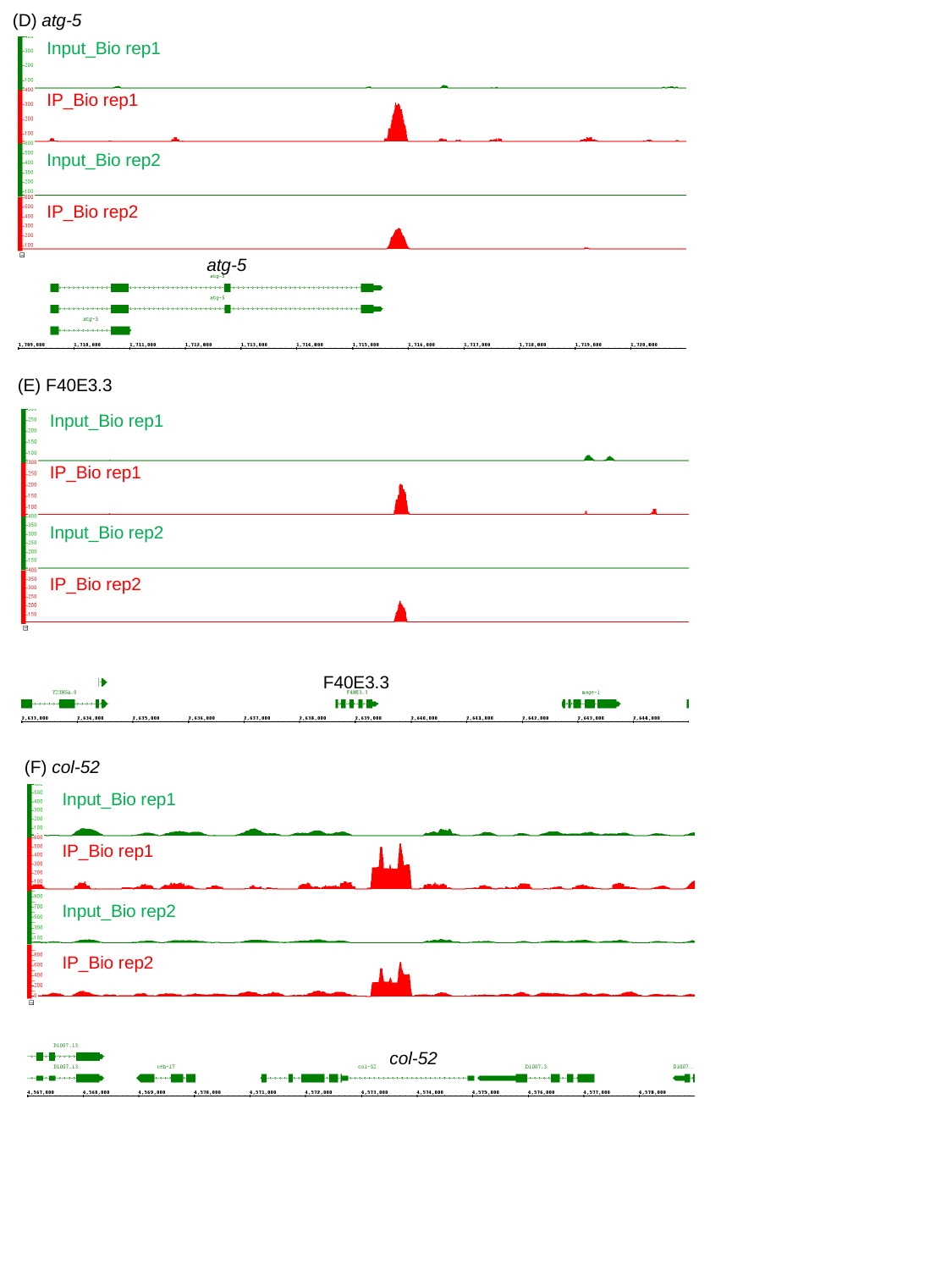

(D) atg-5
Input_Bio rep1
IP_Bio rep1
Input_Bio rep2
IP_Bio rep2
atg-5
(E) F40E3.3
Input_Bio rep1
IP_Bio rep1
Input_Bio rep2
IP_Bio rep2
F40E3.3
(F) col-52
Input_Bio rep1
IP_Bio rep1
Input_Bio rep2
IP_Bio rep2
col-52

## Slide 3
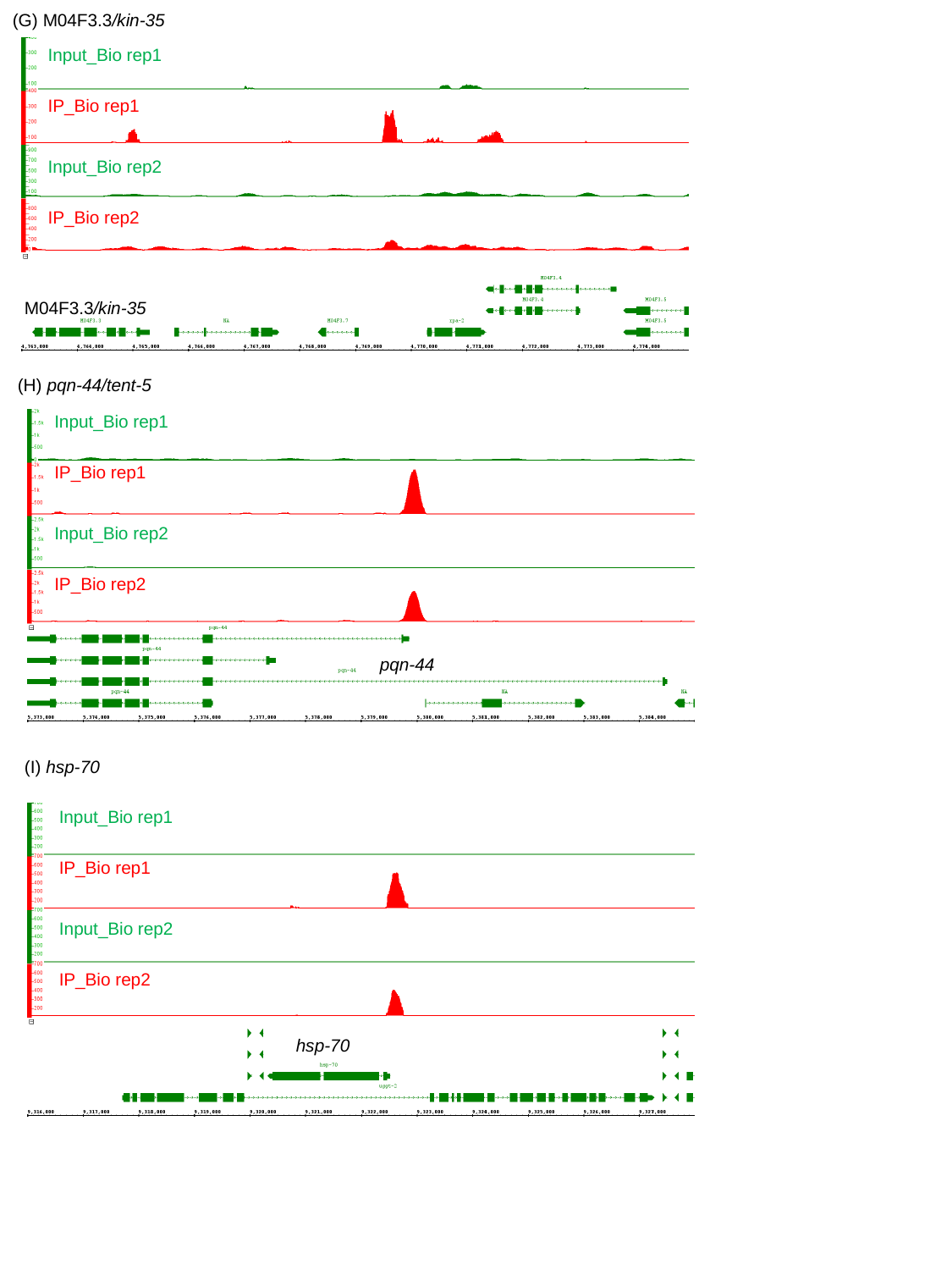

(G) M04F3.3/kin-35
Input_Bio rep1
IP_Bio rep1
Input_Bio rep2
IP_Bio rep2
M04F3.3/kin-35
(H) pqn-44/tent-5
Input_Bio rep1
IP_Bio rep1
Input_Bio rep2
IP_Bio rep2
pqn-44
(I) hsp-70
Input_Bio rep1
IP_Bio rep1
Input_Bio rep2
IP_Bio rep2
hsp-70

## Slide 4
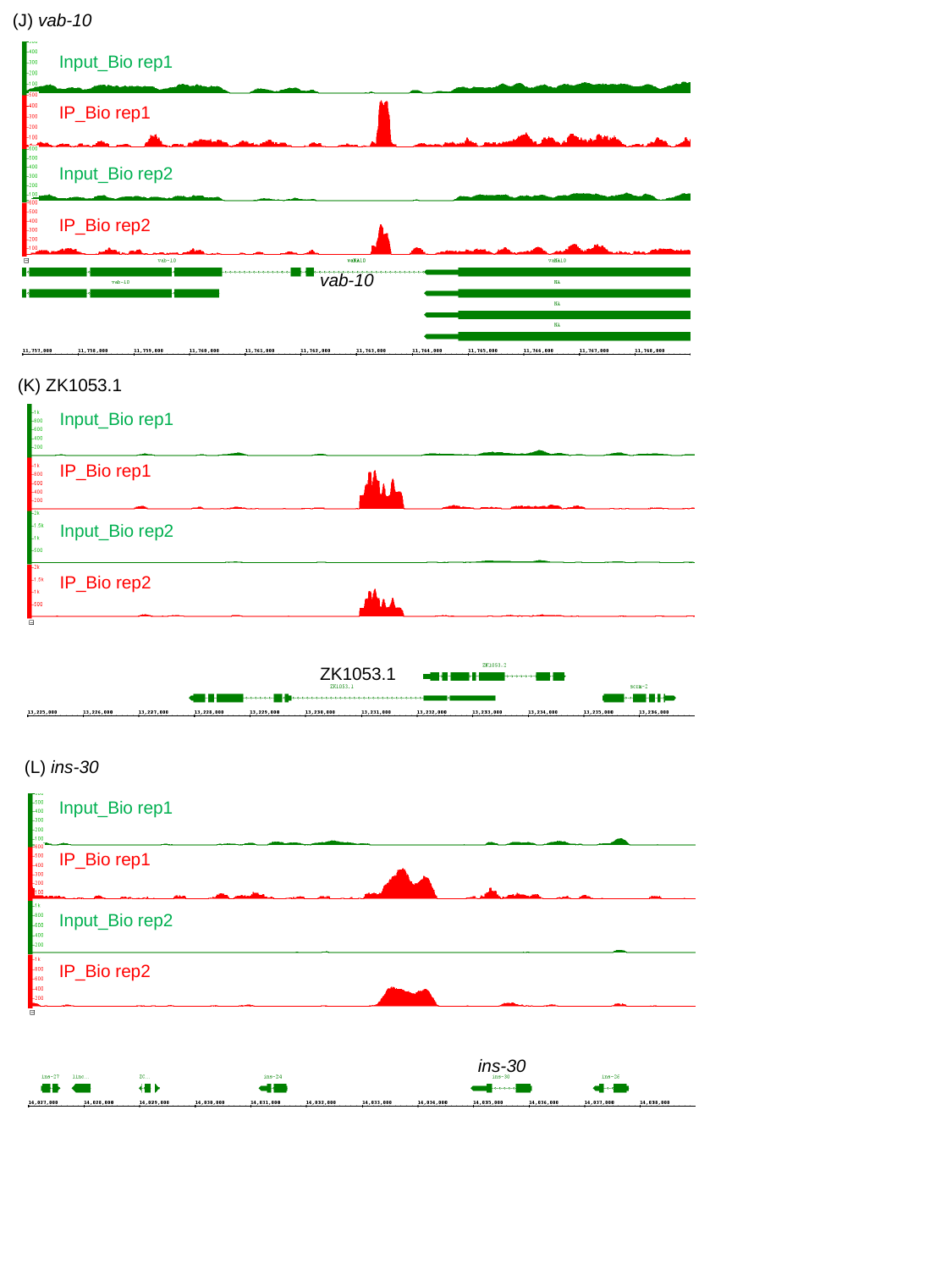

(J) vab-10
Input_Bio rep1
IP_Bio rep1
Input_Bio rep2
IP_Bio rep2
vab-10
(K) ZK1053.1
Input_Bio rep1
IP_Bio rep1
Input_Bio rep2
IP_Bio rep2
ZK1053.1
(L) ins-30
Input_Bio rep1
IP_Bio rep1
Input_Bio rep2
IP_Bio rep2
ins-30

## Slide 5
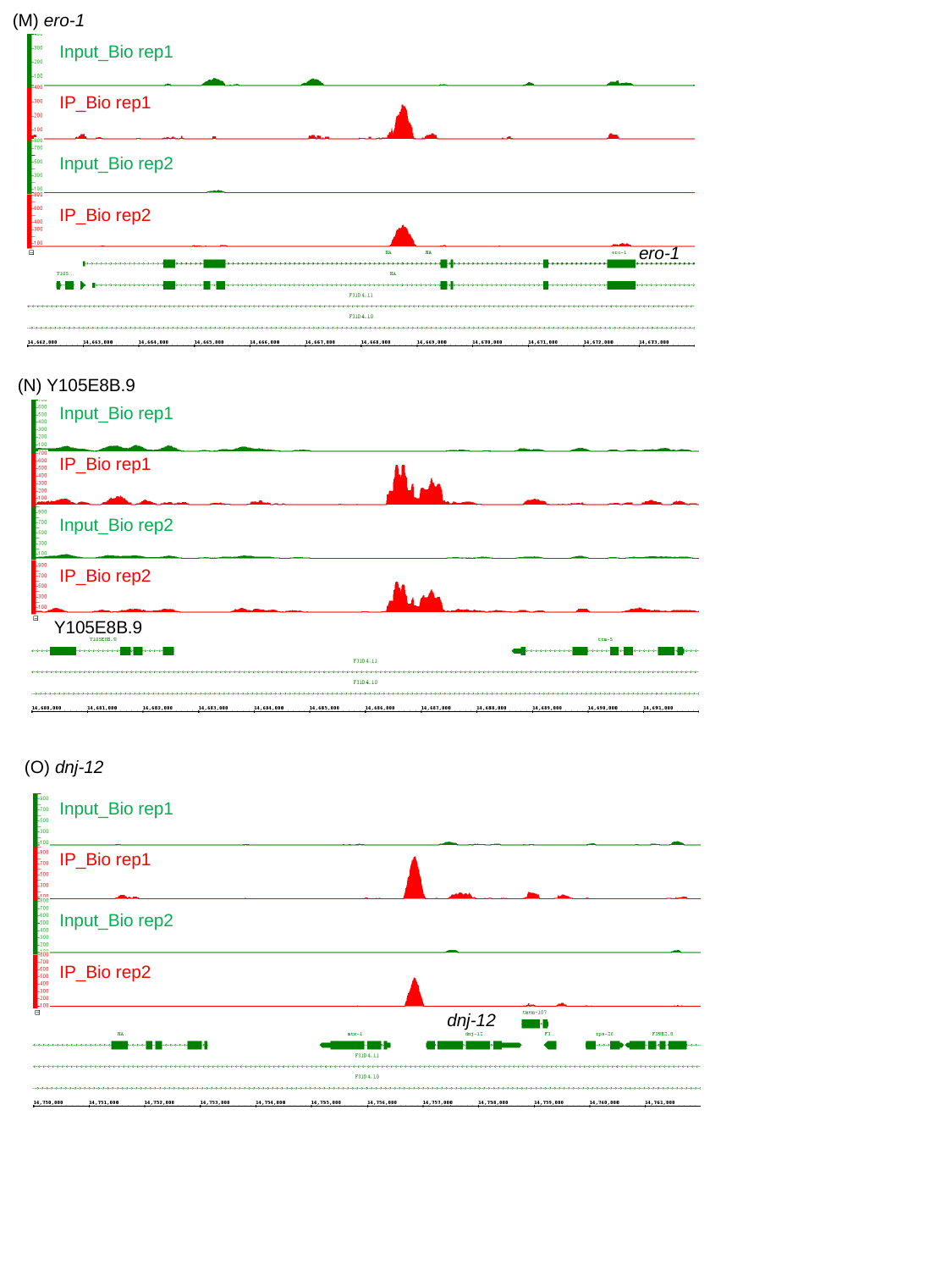

(M) ero-1
Input_Bio rep1
IP_Bio rep1
Input_Bio rep2
IP_Bio rep2
ero-1
(N) Y105E8B.9
Input_Bio rep1
IP_Bio rep1
Input_Bio rep2
IP_Bio rep2
Y105E8B.9
(O) dnj-12
Input_Bio rep1
IP_Bio rep1
Input_Bio rep2
IP_Bio rep2
dnj-12
