# Supplementary material for: Whole genome profiling of short-term hypoxia induced genes and identification of HIF-1 binding sites provide insights into HIF-1 function in Caenorhabditis elegans
Source: PLoS One. 2024 May 14;19(5):e0295094. doi: 10.1371/journal.pone.0295094 (PMC11093353; doi:10.1371/journal.pone.0295094)
Supplement: S2 Fig — (PPTX) [file pone.0295094.s002.pptx]

## Slide 1
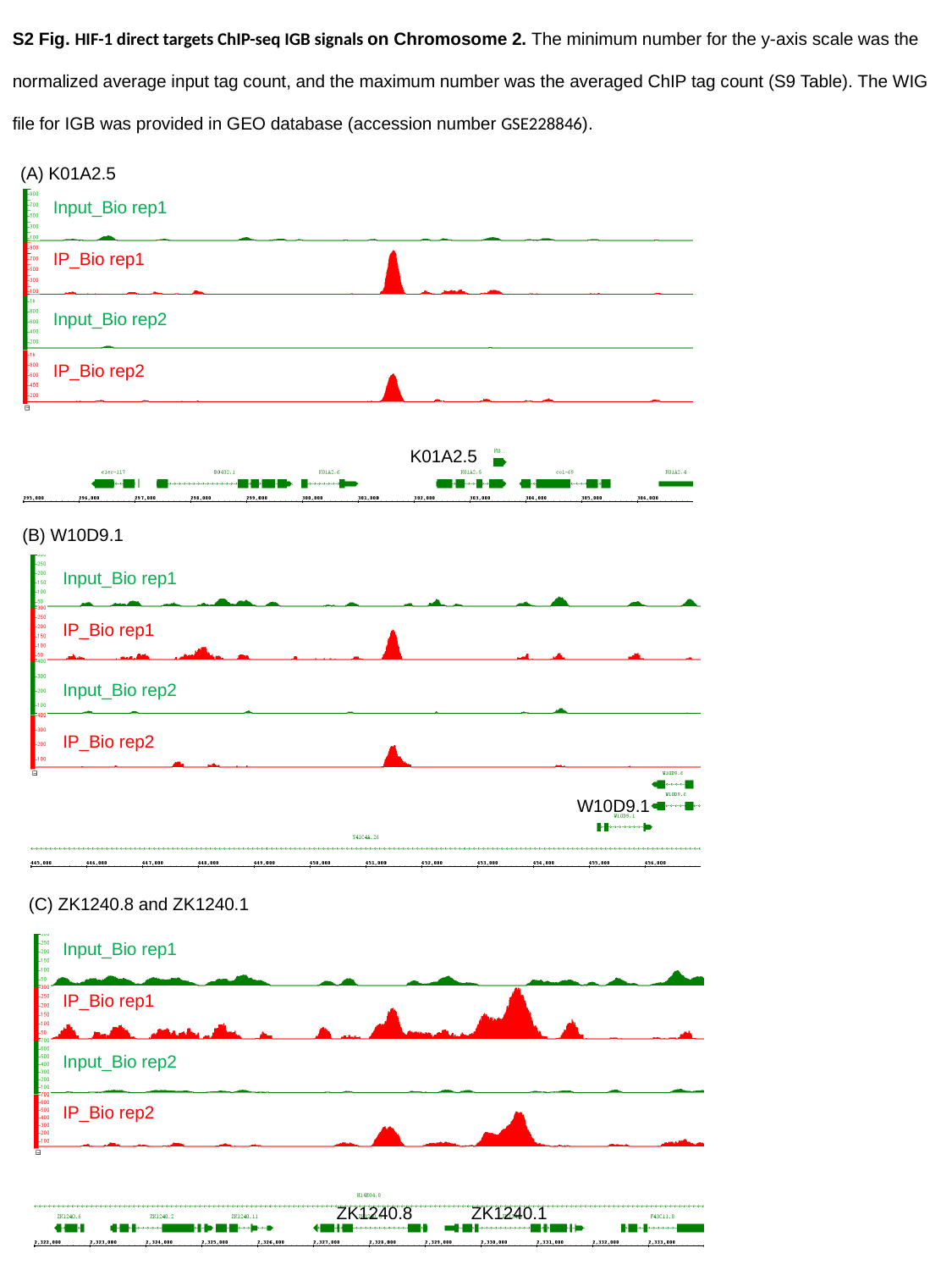

S2 Fig. HIF-1 direct targets ChIP-seq IGB signals on Chromosome 2. The minimum number for the y-axis scale was the normalized average input tag count, and the maximum number was the averaged ChIP tag count (S9 Table). The WIG file for IGB was provided in GEO database (accession number GSE228846).
(A) K01A2.5
Input_Bio rep1
IP_Bio rep1
Input_Bio rep2
IP_Bio rep2
K01A2.5
(B) W10D9.1
Input_Bio rep1
IP_Bio rep1
Input_Bio rep2
IP_Bio rep2
W10D9.1
(C) ZK1240.8 and ZK1240.1
Input_Bio rep1
IP_Bio rep1
Input_Bio rep2
IP_Bio rep2
ZK1240.8
ZK1240.1

## Slide 2
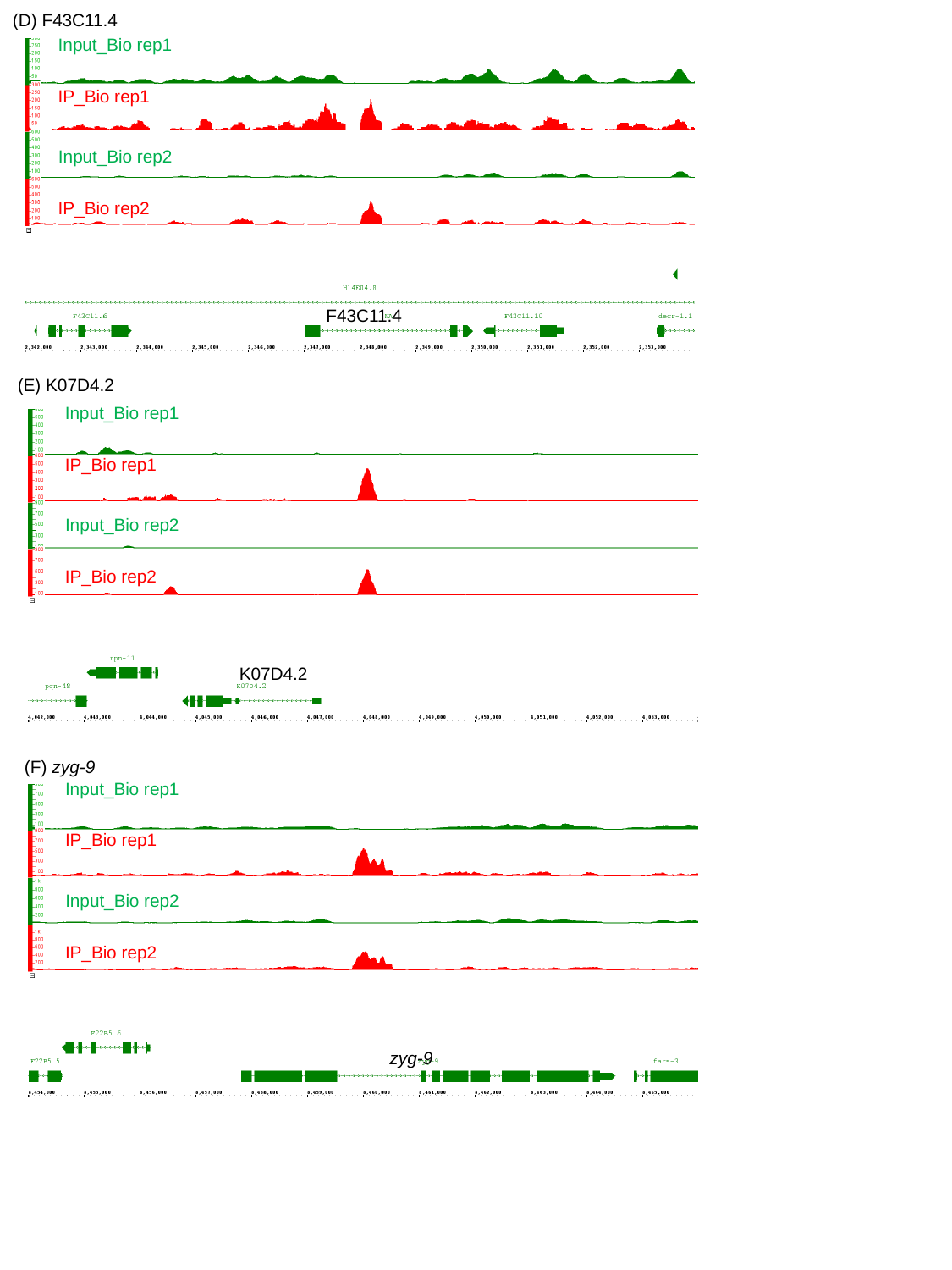

(D) F43C11.4
Input_Bio rep1
IP_Bio rep1
Input_Bio rep2
IP_Bio rep2
F43C11.4
(E) K07D4.2
Input_Bio rep1
IP_Bio rep1
Input_Bio rep2
IP_Bio rep2
K07D4.2
(F) zyg-9
Input_Bio rep1
IP_Bio rep1
Input_Bio rep2
IP_Bio rep2
zyg-9

## Slide 3
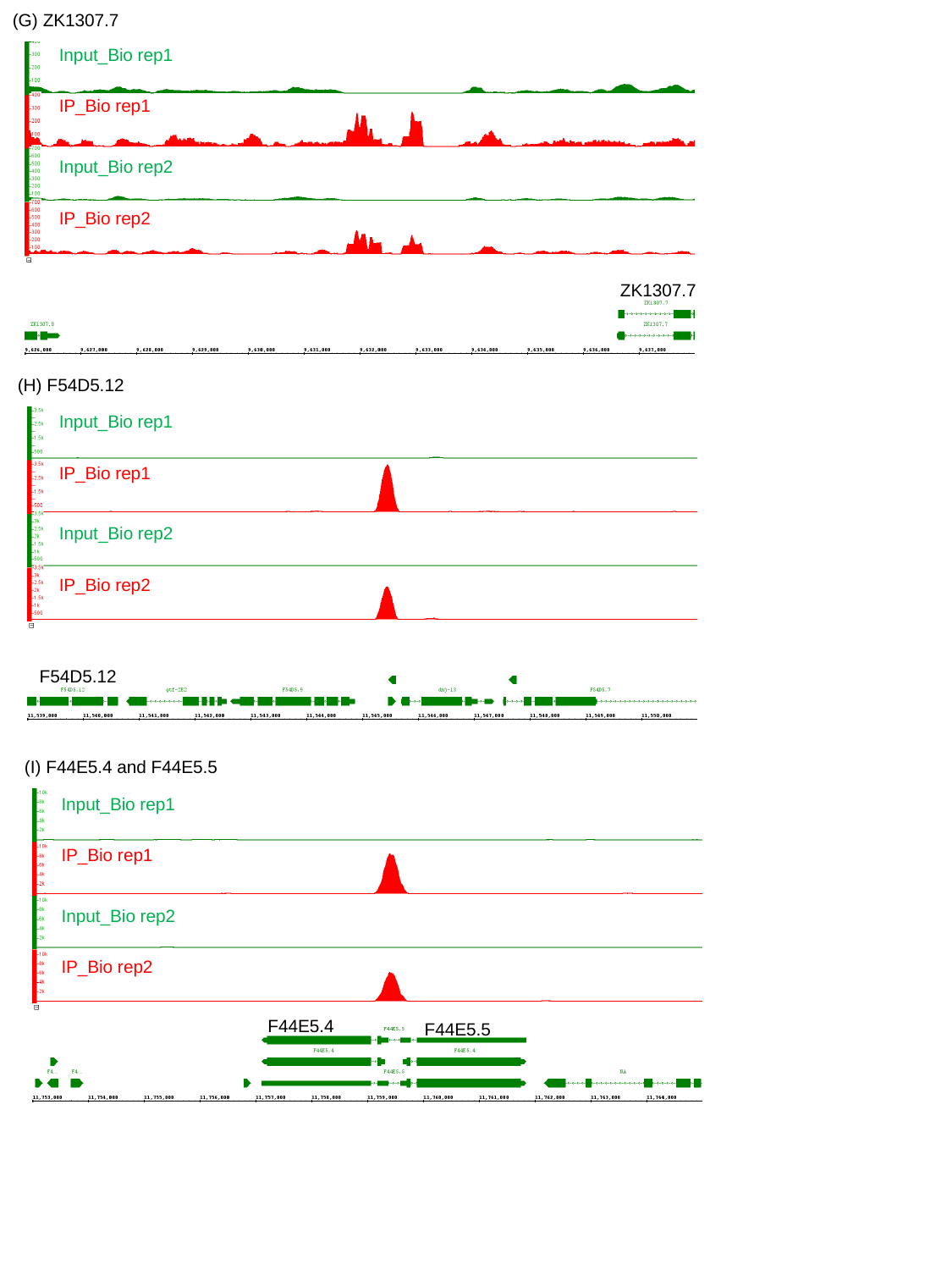

(G) ZK1307.7
Input_Bio rep1
IP_Bio rep1
Input_Bio rep2
IP_Bio rep2
ZK1307.7
(H) F54D5.12
Input_Bio rep1
IP_Bio rep1
Input_Bio rep2
IP_Bio rep2
F54D5.12
(I) F44E5.4 and F44E5.5
Input_Bio rep1
IP_Bio rep1
Input_Bio rep2
IP_Bio rep2
F44E5.4
F44E5.5

## Slide 4
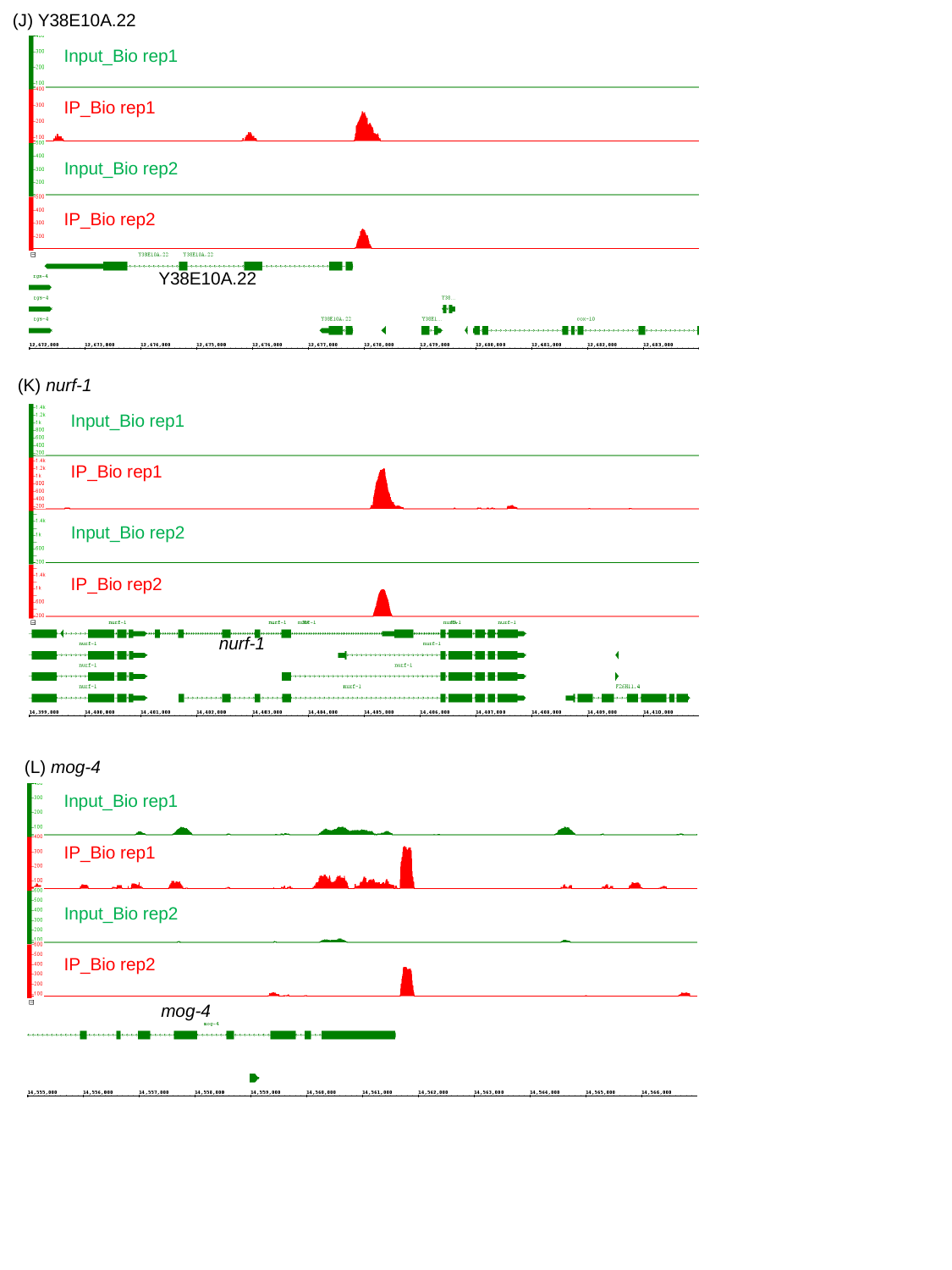

(J) Y38E10A.22
Input_Bio rep1
IP_Bio rep1
Input_Bio rep2
IP_Bio rep2
Y38E10A.22
(K) nurf-1
Input_Bio rep1
IP_Bio rep1
Input_Bio rep2
IP_Bio rep2
nurf-1
(L) mog-4
Input_Bio rep1
IP_Bio rep1
Input_Bio rep2
IP_Bio rep2
mog-4

## Slide 5
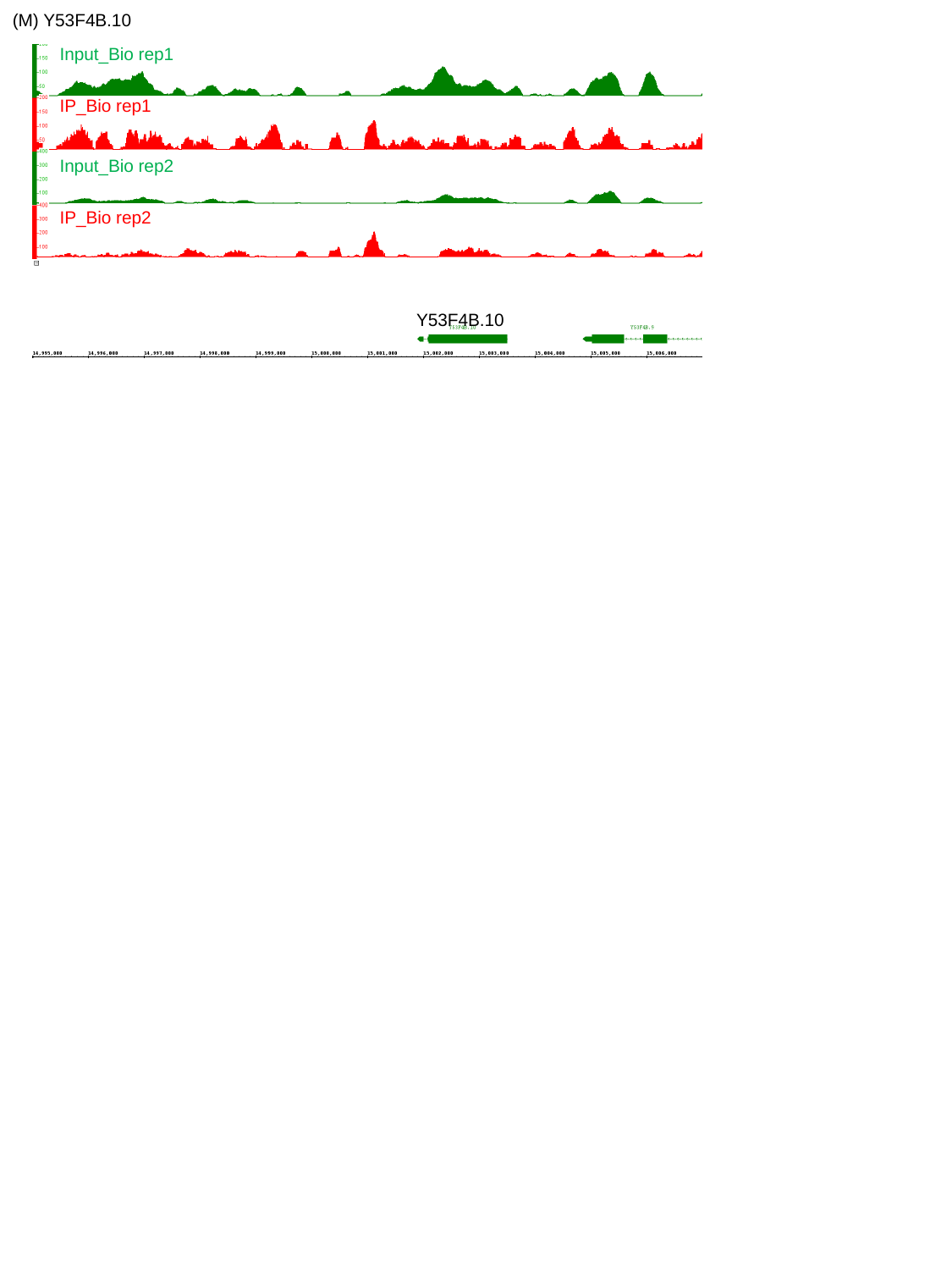

(M) Y53F4B.10
Input_Bio rep1
IP_Bio rep1
Input_Bio rep2
IP_Bio rep2
Y53F4B.10
