# Supplementary material for: Whole genome profiling of short-term hypoxia induced genes and identification of HIF-1 binding sites provide insights into HIF-1 function in Caenorhabditis elegans
Source: PLoS One. 2024 May 14;19(5):e0295094. doi: 10.1371/journal.pone.0295094 (PMC11093353; doi:10.1371/journal.pone.0295094)
Supplement: S3 Fig — (PPTX) [file pone.0295094.s003.pptx]

## Slide 1
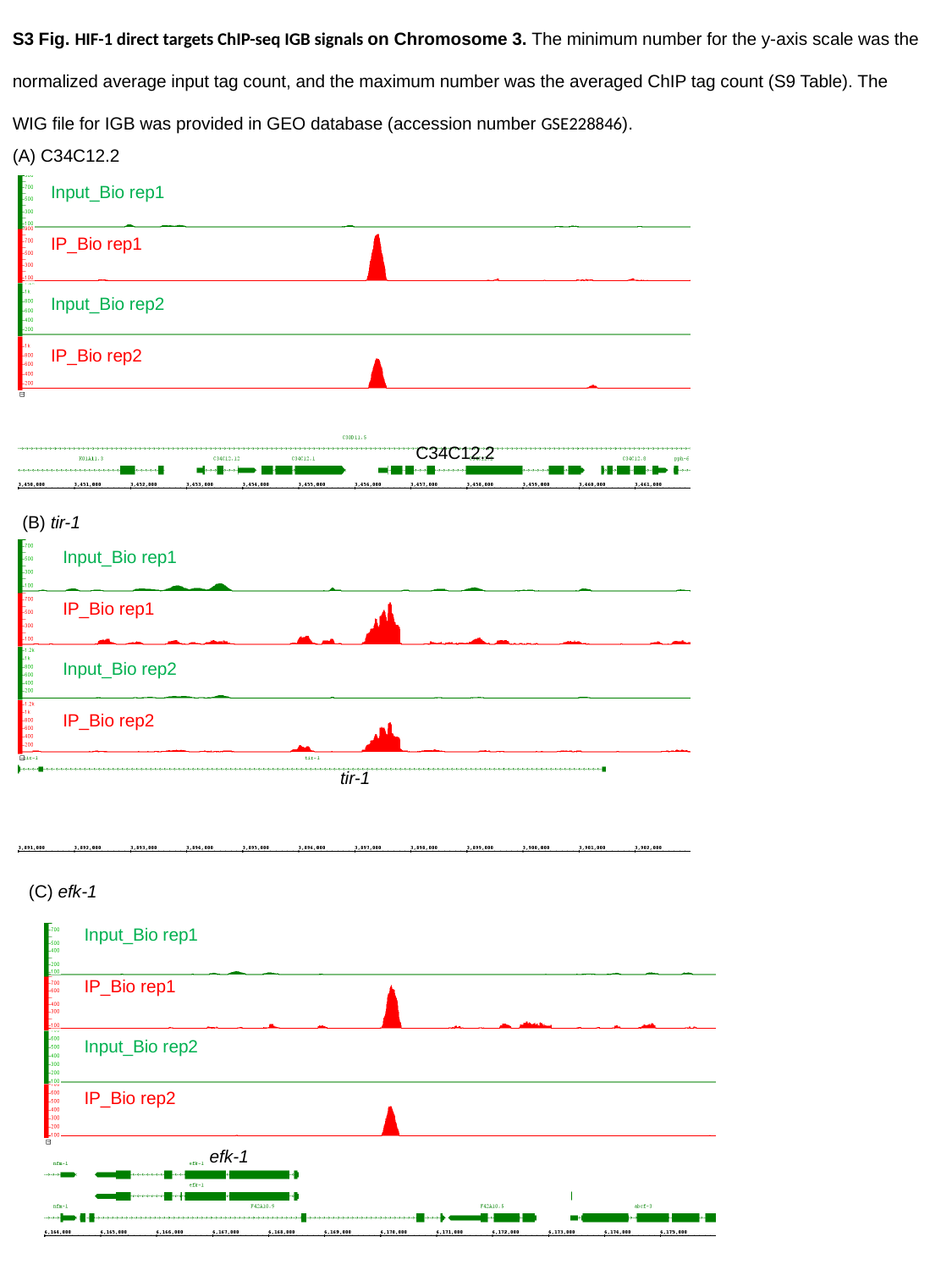

S3 Fig. HIF-1 direct targets ChIP-seq IGB signals on Chromosome 3. The minimum number for the y-axis scale was the normalized average input tag count, and the maximum number was the averaged ChIP tag count (S9 Table). The WIG file for IGB was provided in GEO database (accession number GSE228846).
(A) C34C12.2
Input_Bio rep1
IP_Bio rep1
Input_Bio rep2
IP_Bio rep2
C34C12.2
(B) tir-1
Input_Bio rep1
IP_Bio rep1
Input_Bio rep2
IP_Bio rep2
tir-1
(C) efk-1
Input_Bio rep1
IP_Bio rep1
Input_Bio rep2
IP_Bio rep2
efk-1

## Slide 2
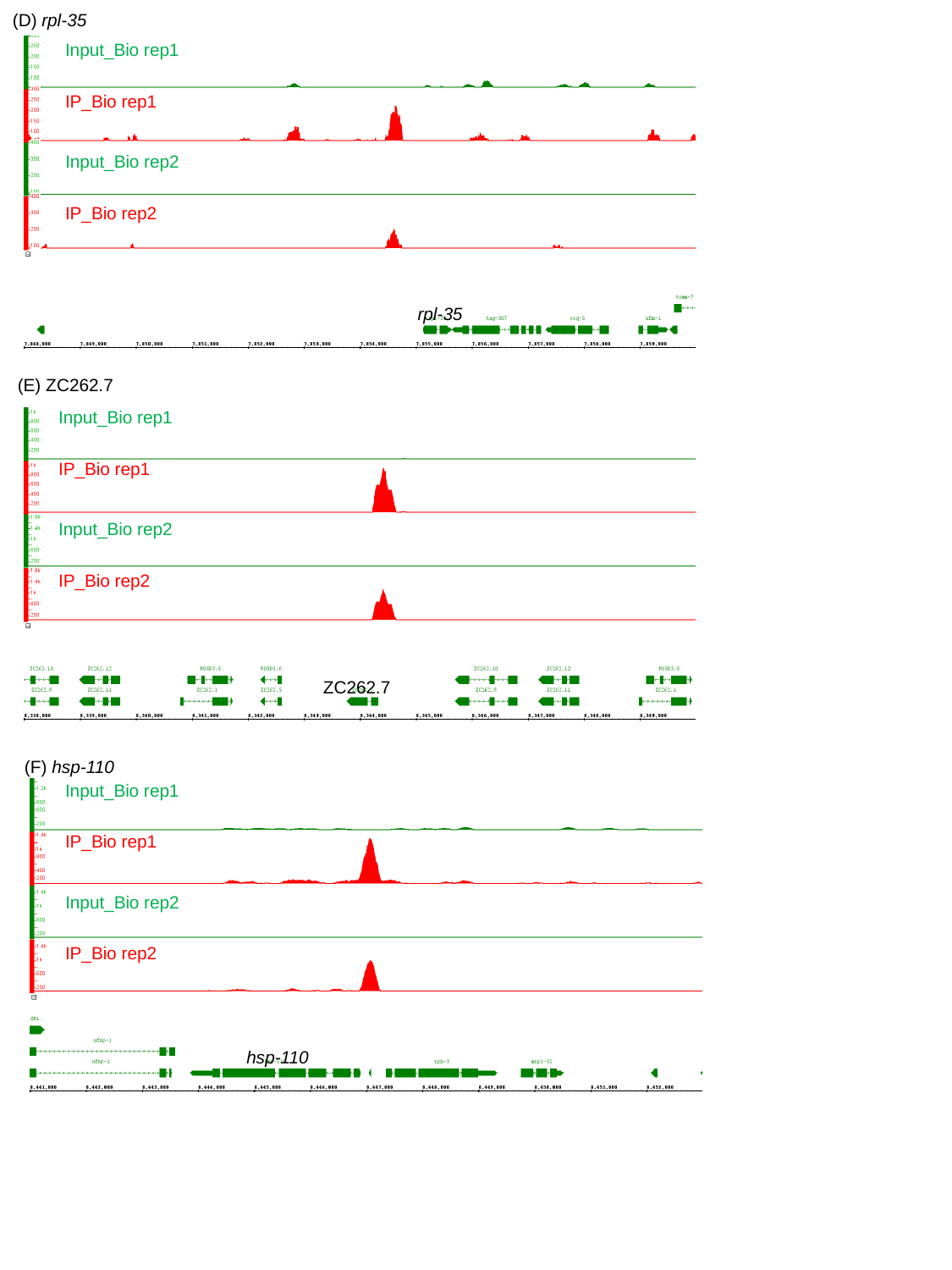

(D) rpl-35
Input_Bio rep1
IP_Bio rep1
Input_Bio rep2
IP_Bio rep2
rpl-35
(E) ZC262.7
Input_Bio rep1
IP_Bio rep1
Input_Bio rep2
IP_Bio rep2
ZC262.7
(F) hsp-110
Input_Bio rep1
IP_Bio rep1
Input_Bio rep2
IP_Bio rep2
hsp-110

## Slide 3
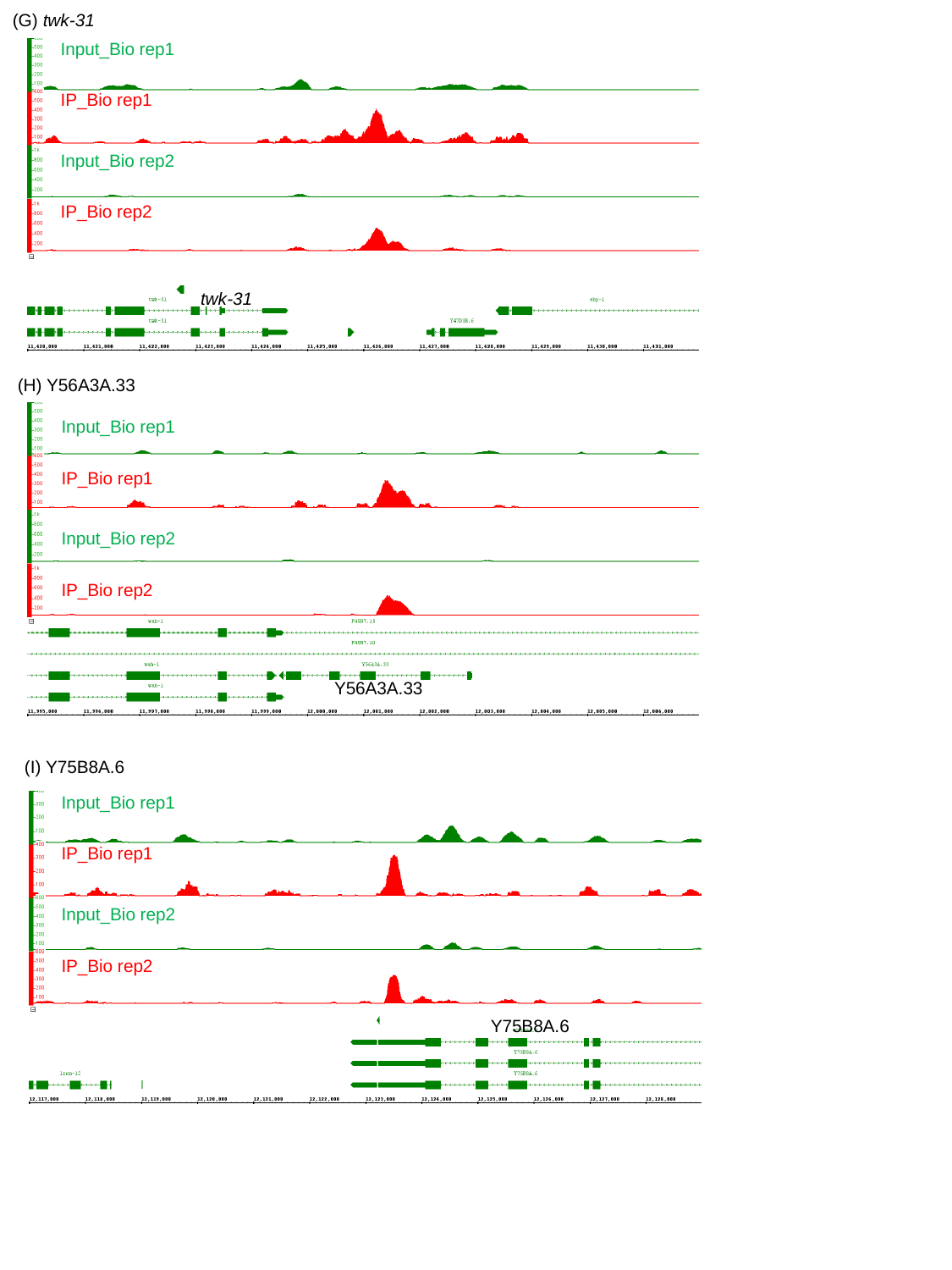

(G) twk-31
Input_Bio rep1
IP_Bio rep1
Input_Bio rep2
IP_Bio rep2
twk-31
(H) Y56A3A.33
Input_Bio rep1
IP_Bio rep1
Input_Bio rep2
IP_Bio rep2
Y56A3A.33
(I) Y75B8A.6
Input_Bio rep1
IP_Bio rep1
Input_Bio rep2
IP_Bio rep2
Y75B8A.6

## Slide 4
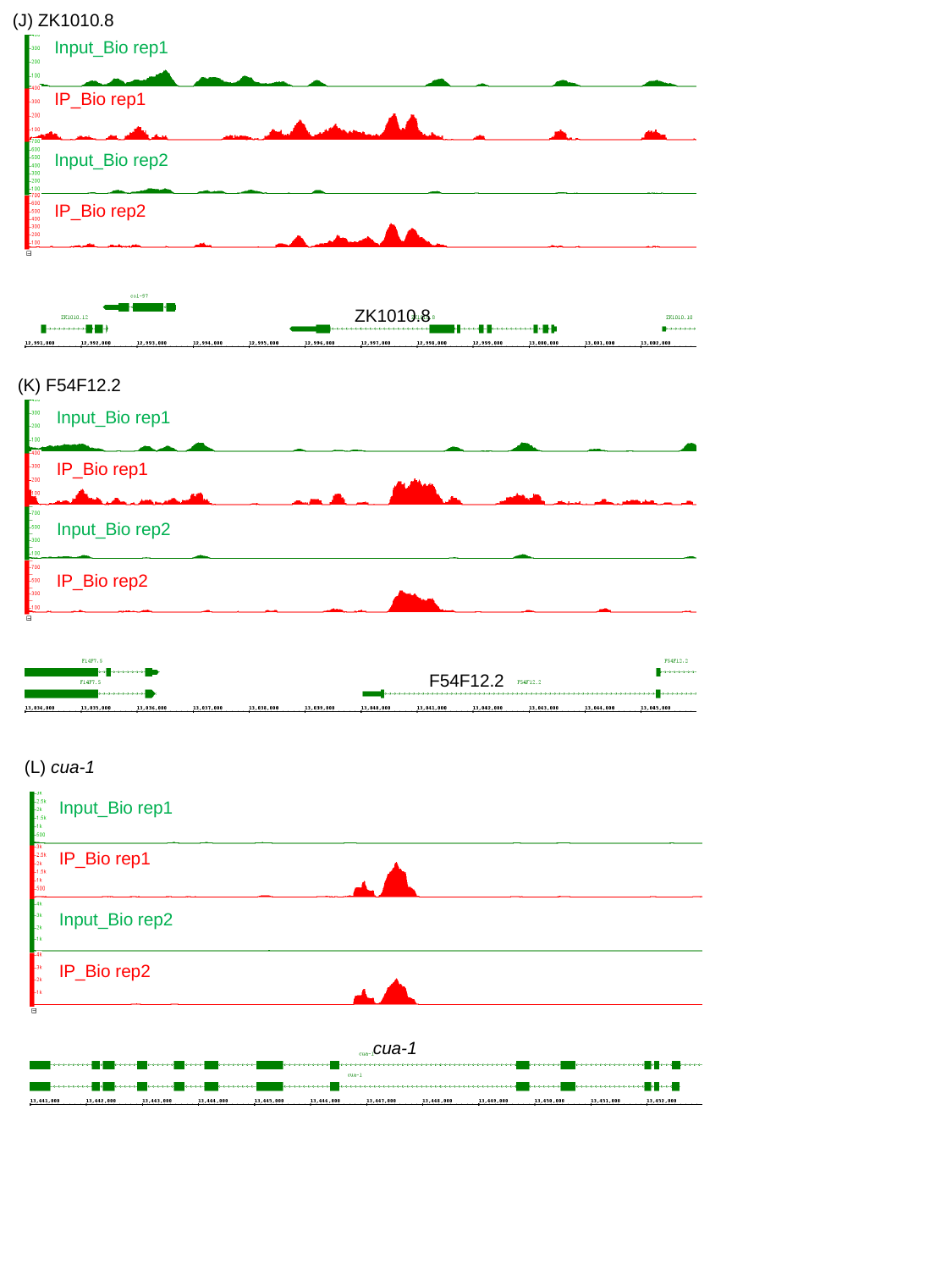

(J) ZK1010.8
Input_Bio rep1
IP_Bio rep1
Input_Bio rep2
IP_Bio rep2
ZK1010.8
(K) F54F12.2
Input_Bio rep1
IP_Bio rep1
Input_Bio rep2
IP_Bio rep2
F54F12.2
(L) cua-1
Input_Bio rep1
IP_Bio rep1
Input_Bio rep2
IP_Bio rep2
cua-1
