# Supplementary material for: Whole genome profiling of short-term hypoxia induced genes and identification of HIF-1 binding sites provide insights into HIF-1 function in Caenorhabditis elegans
Source: PLoS One. 2024 May 14;19(5):e0295094. doi: 10.1371/journal.pone.0295094 (PMC11093353; doi:10.1371/journal.pone.0295094)
Supplement: S4 Fig — (PPTX) [file pone.0295094.s004.pptx]

## Slide 1
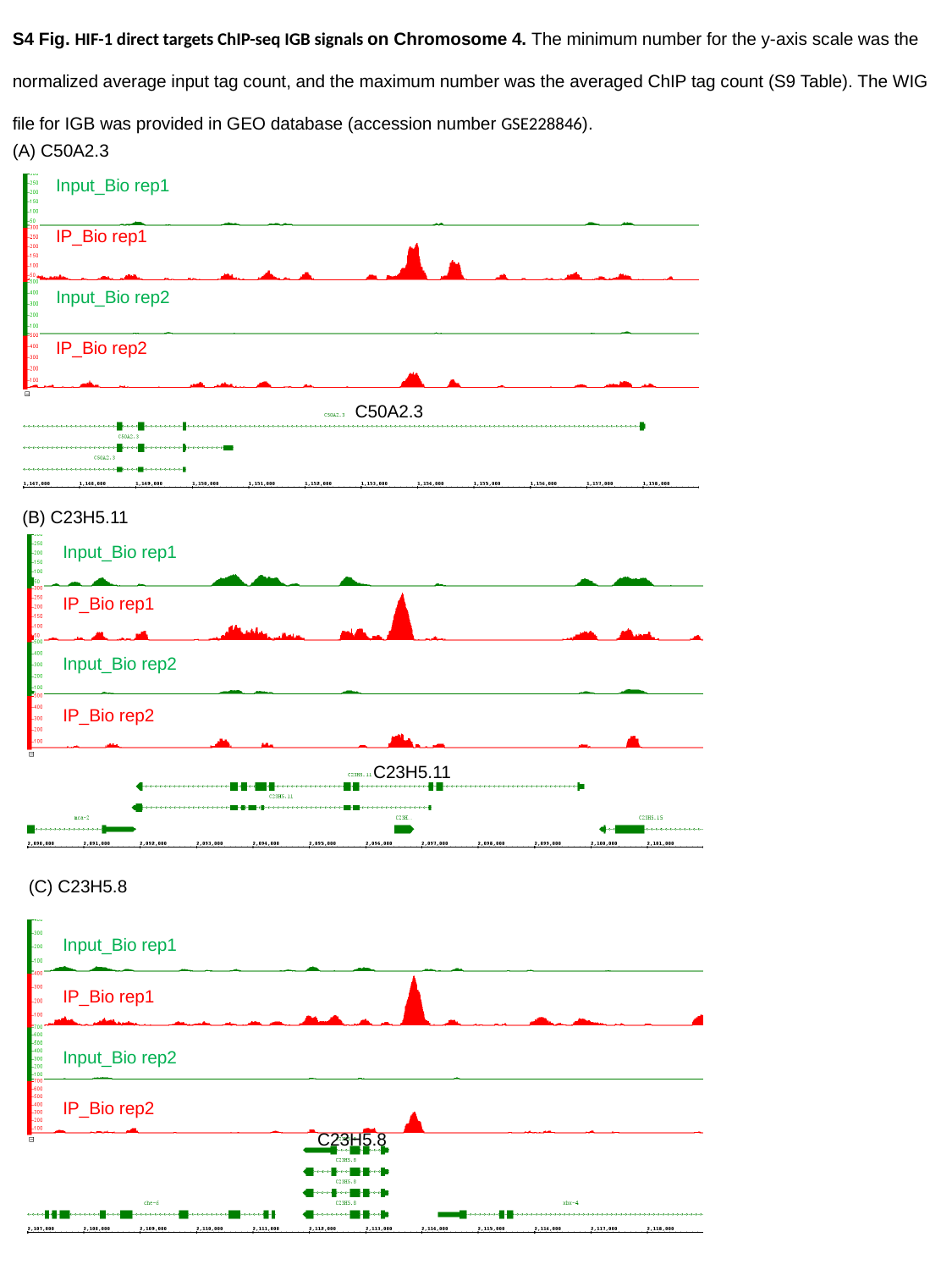

S4 Fig. HIF-1 direct targets ChIP-seq IGB signals on Chromosome 4. The minimum number for the y-axis scale was the normalized average input tag count, and the maximum number was the averaged ChIP tag count (S9 Table). The WIG file for IGB was provided in GEO database (accession number GSE228846).
(A) C50A2.3
Input_Bio rep1
IP_Bio rep1
Input_Bio rep2
IP_Bio rep2
C50A2.3
(B) C23H5.11
Input_Bio rep1
IP_Bio rep1
Input_Bio rep2
IP_Bio rep2
C23H5.11
(C) C23H5.8
Input_Bio rep1
IP_Bio rep1
Input_Bio rep2
IP_Bio rep2
C23H5.8

## Slide 2
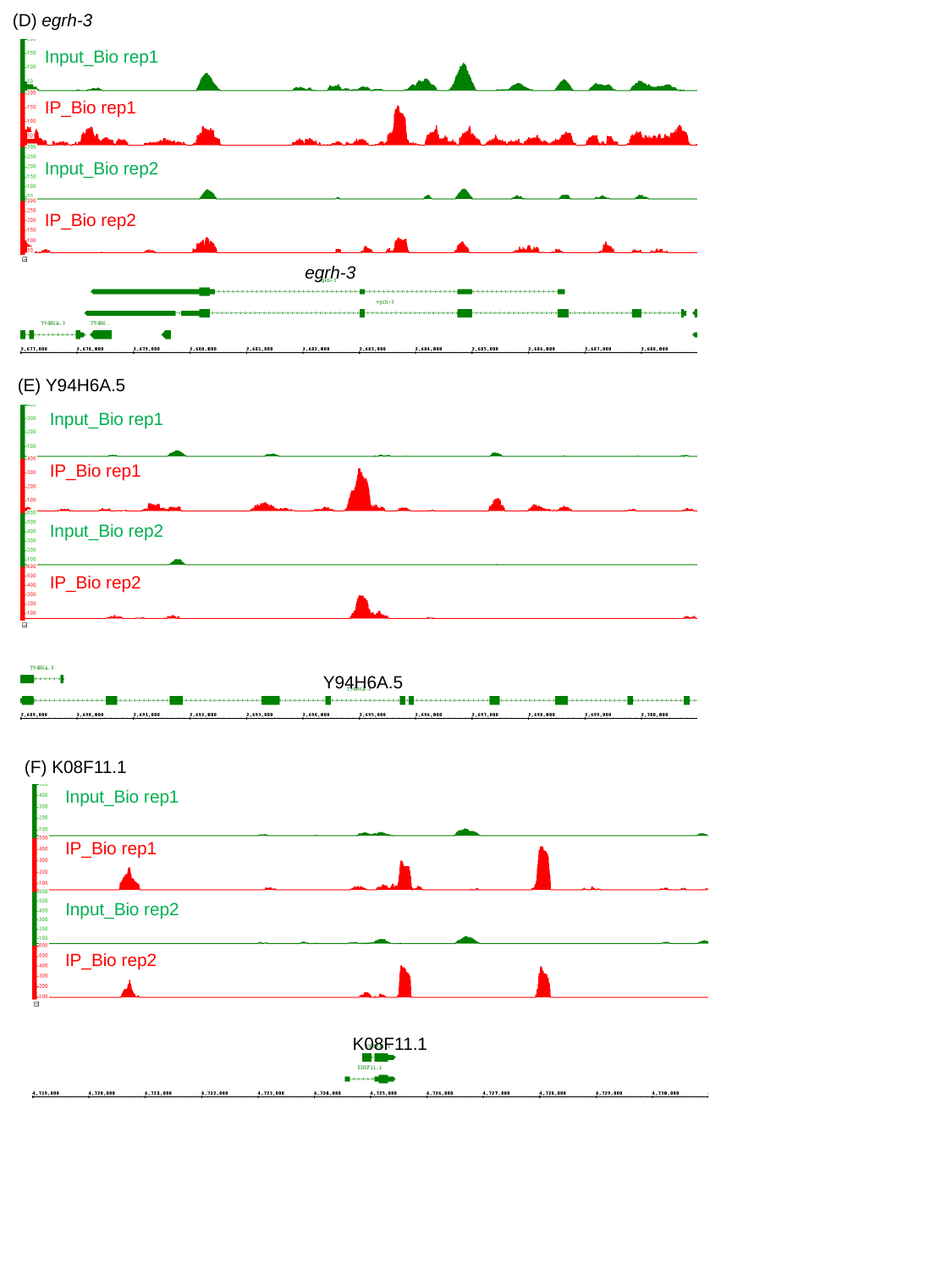

(D) egrh-3
Input_Bio rep1
IP_Bio rep1
Input_Bio rep2
IP_Bio rep2
egrh-3
(E) Y94H6A.5
Input_Bio rep1
IP_Bio rep1
Input_Bio rep2
IP_Bio rep2
Y94H6A.5
(F) K08F11.1
Input_Bio rep1
IP_Bio rep1
Input_Bio rep2
IP_Bio rep2
K08F11.1

## Slide 3
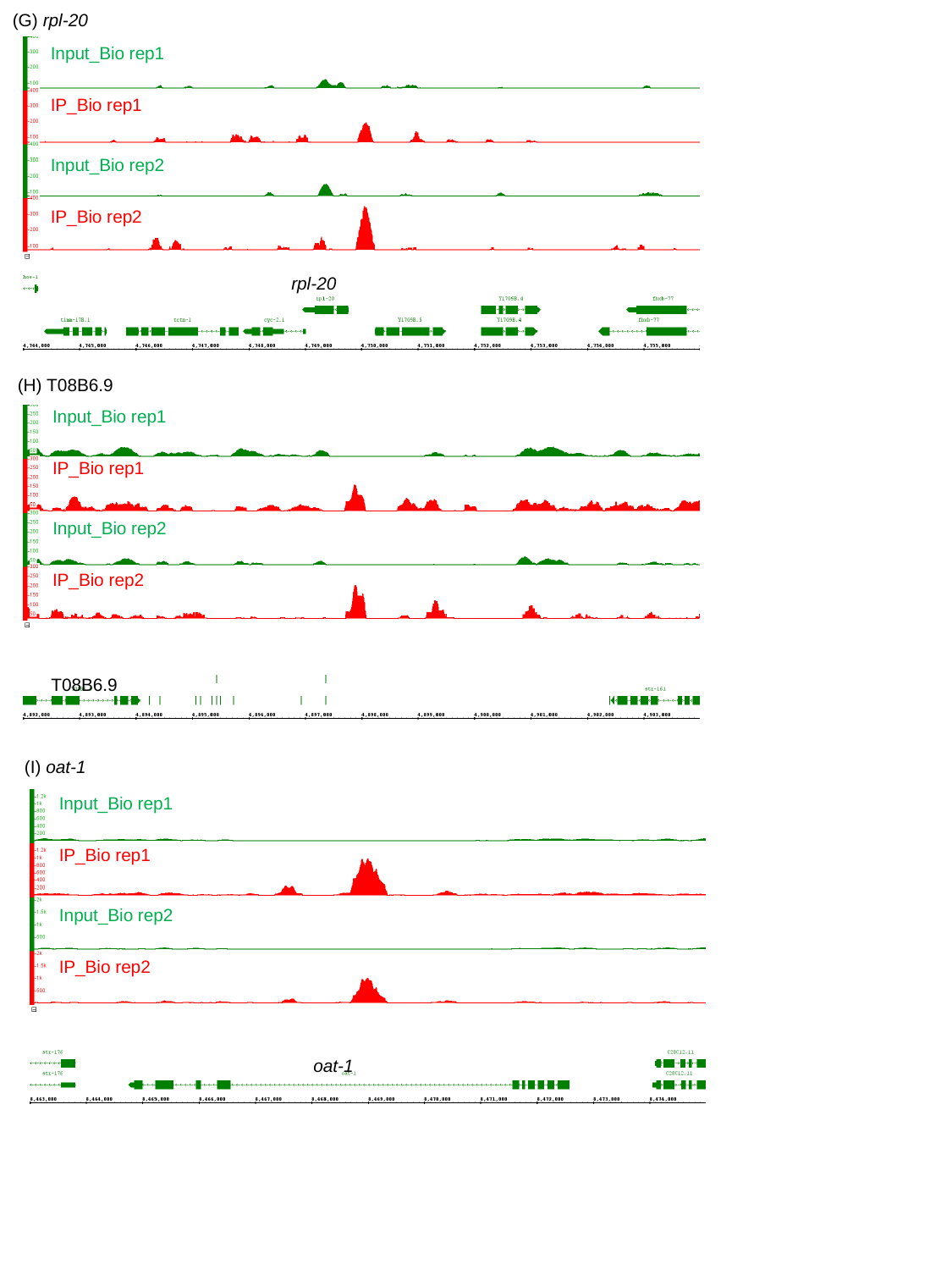

(G) rpl-20
Input_Bio rep1
IP_Bio rep1
Input_Bio rep2
IP_Bio rep2
rpl-20
(H) T08B6.9
Input_Bio rep1
IP_Bio rep1
Input_Bio rep2
IP_Bio rep2
T08B6.9
(I) oat-1
Input_Bio rep1
IP_Bio rep1
Input_Bio rep2
IP_Bio rep2
oat-1

## Slide 4
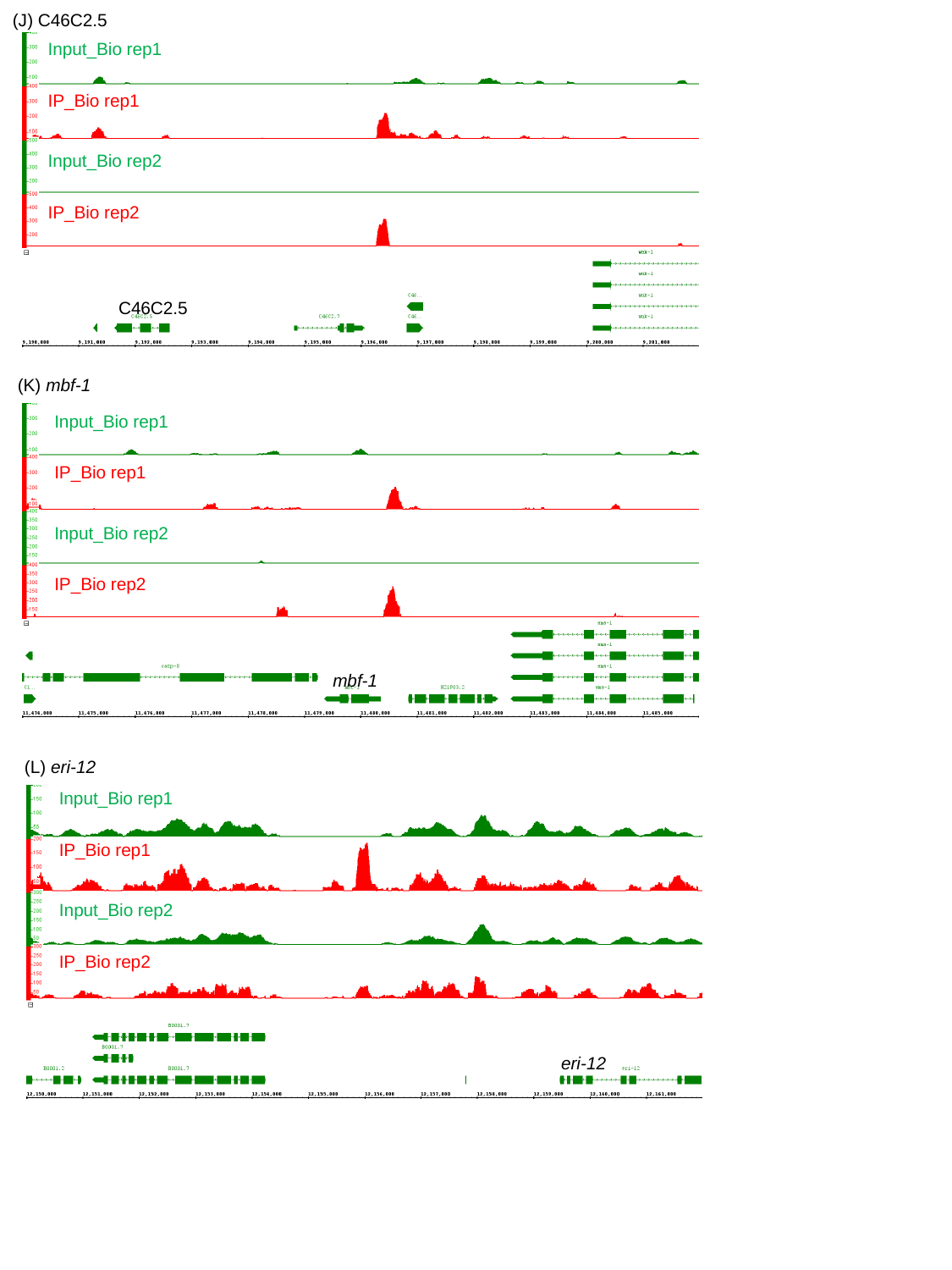

(J) C46C2.5
Input_Bio rep1
IP_Bio rep1
Input_Bio rep2
IP_Bio rep2
C46C2.5
(K) mbf-1
Input_Bio rep1
IP_Bio rep1
Input_Bio rep2
IP_Bio rep2
mbf-1
(L) eri-12
Input_Bio rep1
IP_Bio rep1
Input_Bio rep2
IP_Bio rep2
eri-12

## Slide 5
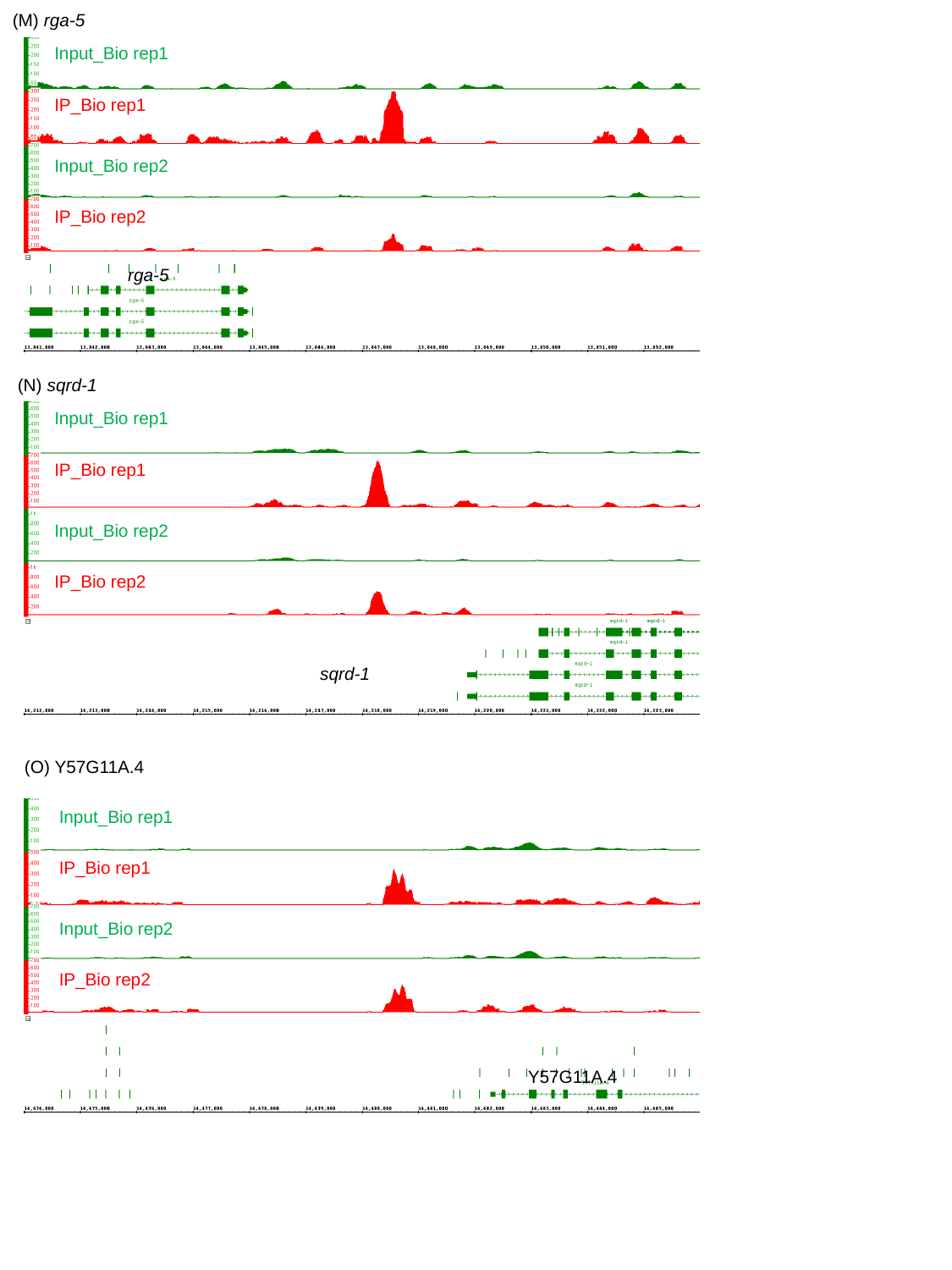

(M) rga-5
Input_Bio rep1
IP_Bio rep1
Input_Bio rep2
IP_Bio rep2
rga-5
(N) sqrd-1
Input_Bio rep1
IP_Bio rep1
Input_Bio rep2
IP_Bio rep2
sqrd-1
(O) Y57G11A.4
Input_Bio rep1
IP_Bio rep1
Input_Bio rep2
IP_Bio rep2
Y57G11A.4

## Slide 6
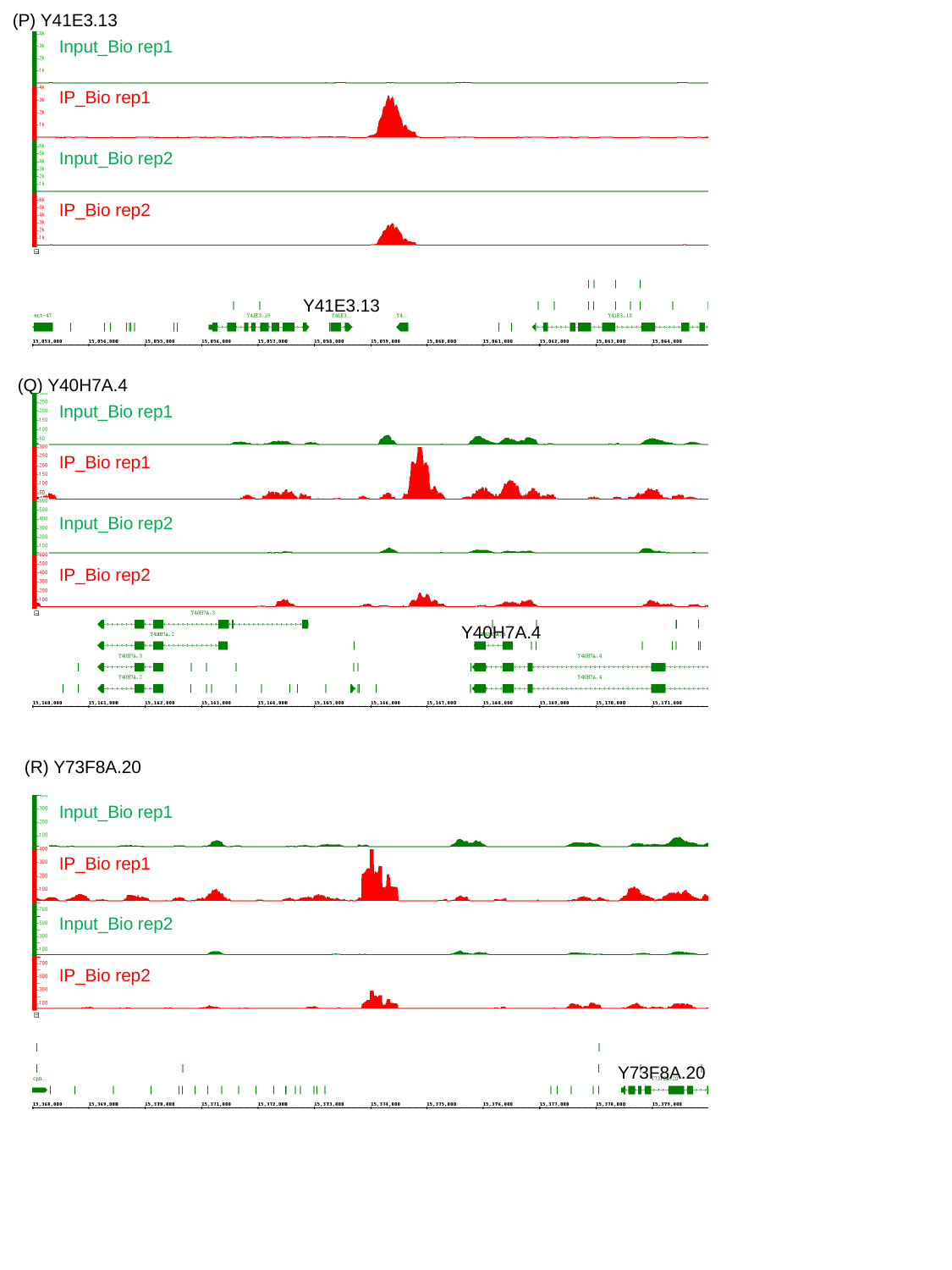

(P) Y41E3.13
Input_Bio rep1
IP_Bio rep1
Input_Bio rep2
IP_Bio rep2
Y41E3.13
(Q) Y40H7A.4
Input_Bio rep1
IP_Bio rep1
Input_Bio rep2
IP_Bio rep2
Y40H7A.4
(R) Y73F8A.20
Input_Bio rep1
IP_Bio rep1
Input_Bio rep2
IP_Bio rep2
Y73F8A.20

## Slide 7
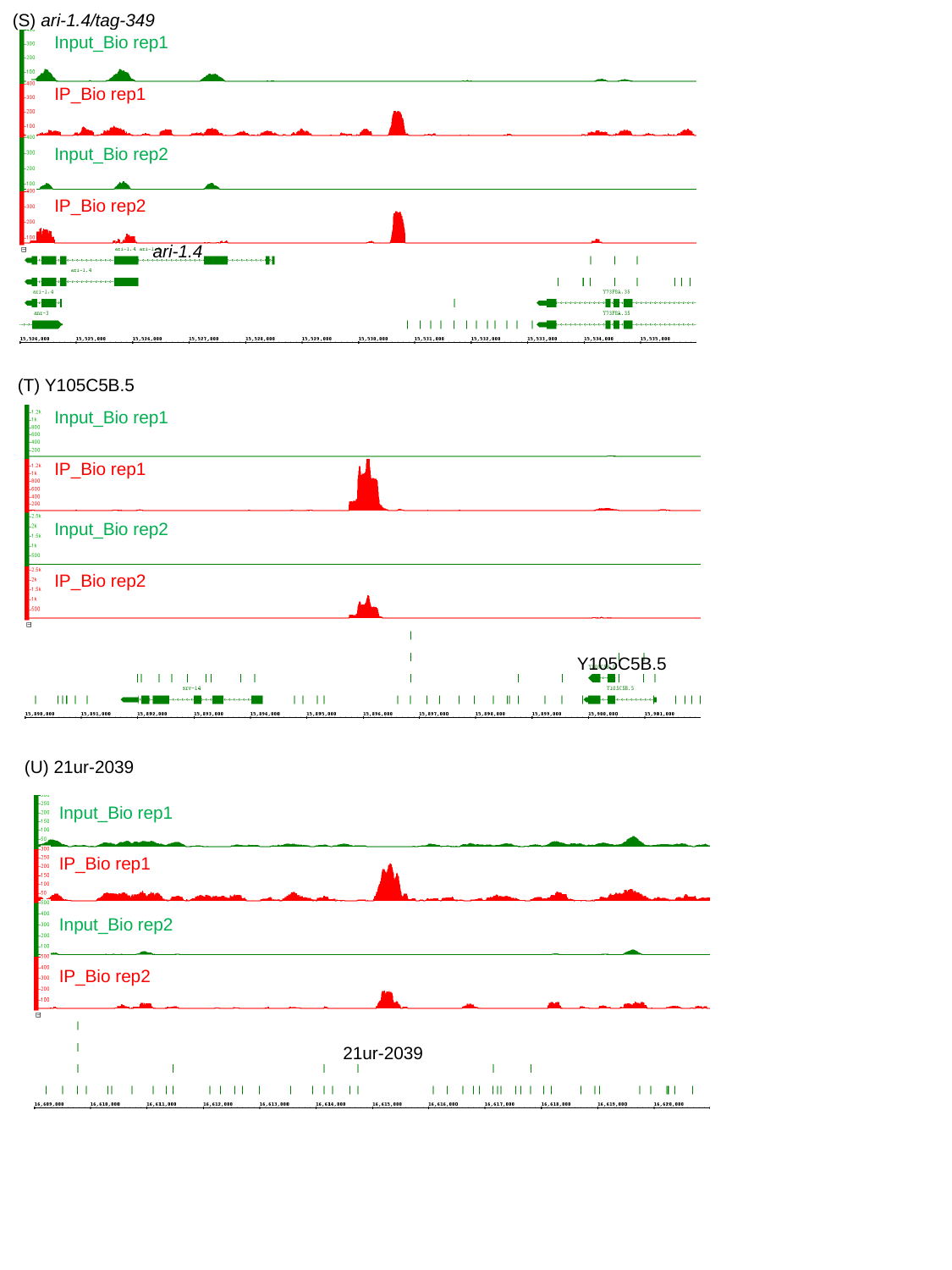

(S) ari-1.4/tag-349
Input_Bio rep1
IP_Bio rep1
Input_Bio rep2
IP_Bio rep2
ari-1.4
(T) Y105C5B.5
Input_Bio rep1
IP_Bio rep1
Input_Bio rep2
IP_Bio rep2
Y105C5B.5
(U) 21ur-2039
Input_Bio rep1
IP_Bio rep1
Input_Bio rep2
IP_Bio rep2
21ur-2039

## Slide 8
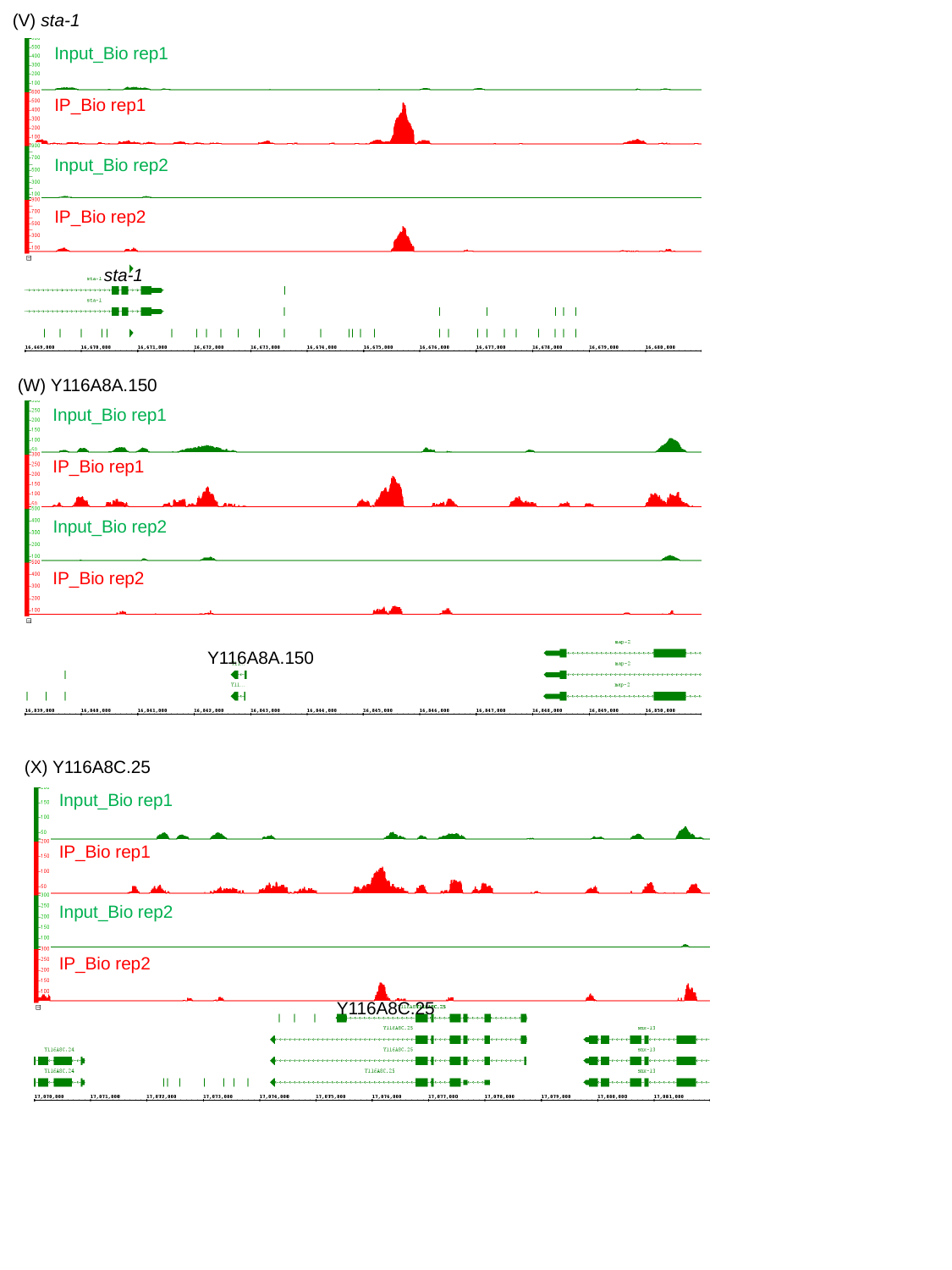

(V) sta-1
Input_Bio rep1
IP_Bio rep1
Input_Bio rep2
IP_Bio rep2
sta-1
(W) Y116A8A.150
Input_Bio rep1
IP_Bio rep1
Input_Bio rep2
IP_Bio rep2
Y116A8A.150
(X) Y116A8C.25
Input_Bio rep1
IP_Bio rep1
Input_Bio rep2
IP_Bio rep2
Y116A8C.25
