# Supplementary material for: Whole genome profiling of short-term hypoxia induced genes and identification of HIF-1 binding sites provide insights into HIF-1 function in Caenorhabditis elegans
Source: PLoS One. 2024 May 14;19(5):e0295094. doi: 10.1371/journal.pone.0295094 (PMC11093353; doi:10.1371/journal.pone.0295094)
Supplement: S5 Fig — (PPTX) [file pone.0295094.s005.pptx]

## Slide 1
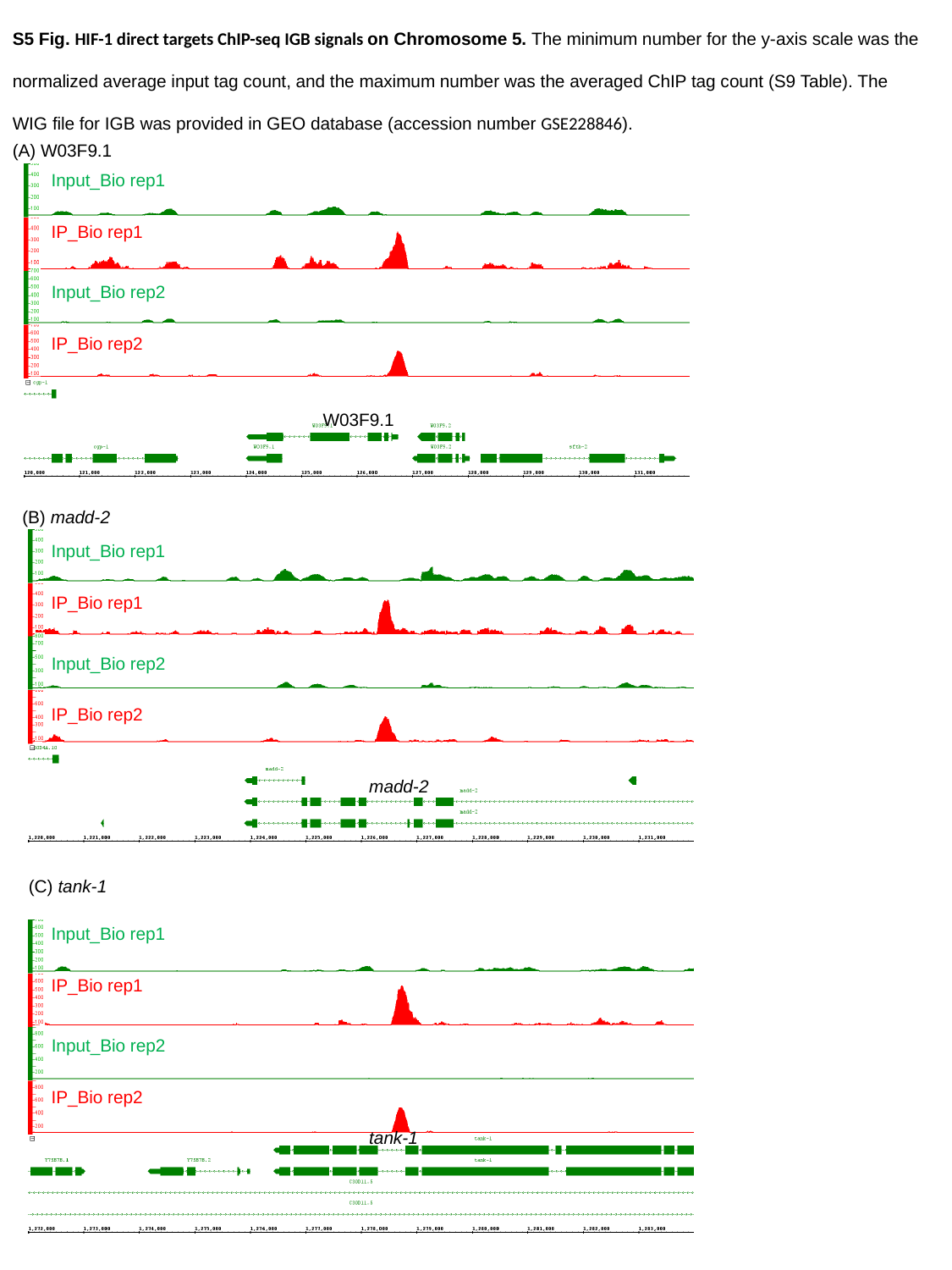

S5 Fig. HIF-1 direct targets ChIP-seq IGB signals on Chromosome 5. The minimum number for the y-axis scale was the normalized average input tag count, and the maximum number was the averaged ChIP tag count (S9 Table). The WIG file for IGB was provided in GEO database (accession number GSE228846).
(A) W03F9.1
Input_Bio rep1
IP_Bio rep1
Input_Bio rep2
IP_Bio rep2
W03F9.1
(B) madd-2
Input_Bio rep1
IP_Bio rep1
Input_Bio rep2
IP_Bio rep2
madd-2
(C) tank-1
Input_Bio rep1
IP_Bio rep1
Input_Bio rep2
IP_Bio rep2
tank-1

## Slide 2
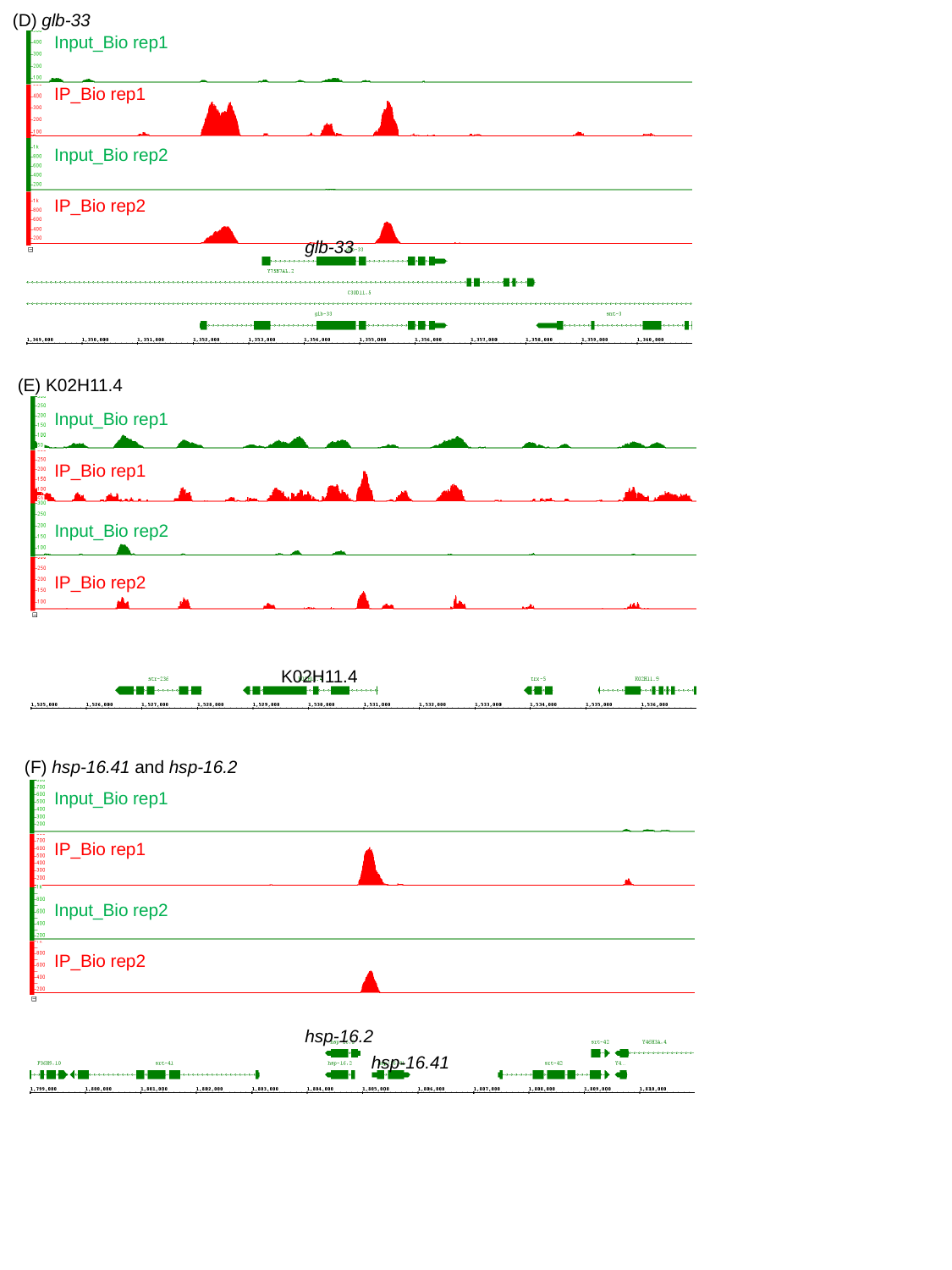

(D) glb-33
Input_Bio rep1
IP_Bio rep1
Input_Bio rep2
IP_Bio rep2
glb-33
(E) K02H11.4
Input_Bio rep1
IP_Bio rep1
Input_Bio rep2
IP_Bio rep2
K02H11.4
(F) hsp-16.41 and hsp-16.2
Input_Bio rep1
IP_Bio rep1
Input_Bio rep2
IP_Bio rep2
hsp-16.2
hsp-16.41

## Slide 3
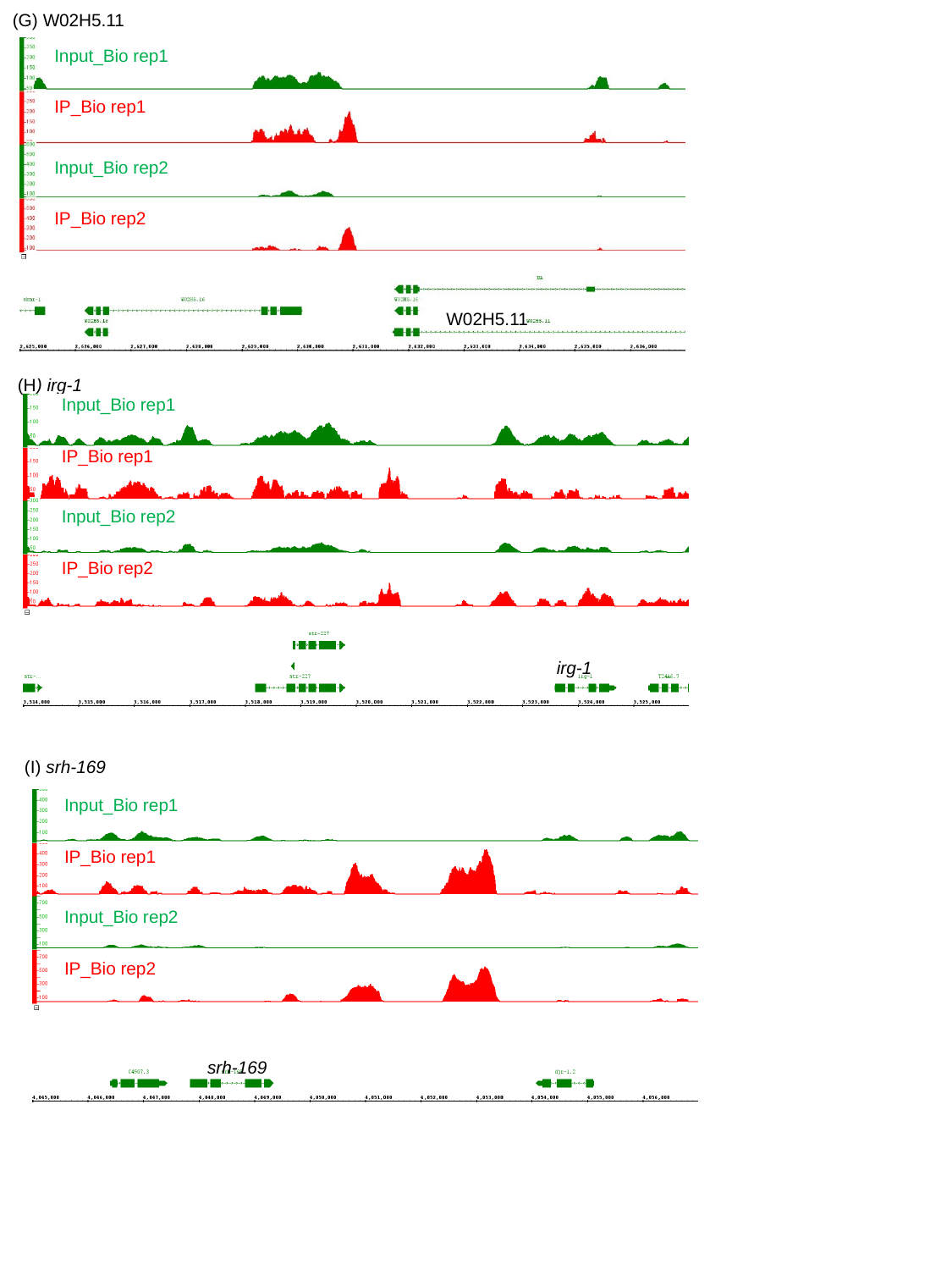

(G) W02H5.11
Input_Bio rep1
IP_Bio rep1
Input_Bio rep2
IP_Bio rep2
W02H5.11
(H) irg-1
Input_Bio rep1
IP_Bio rep1
Input_Bio rep2
IP_Bio rep2
irg-1
(I) srh-169
Input_Bio rep1
IP_Bio rep1
Input_Bio rep2
IP_Bio rep2
srh-169

## Slide 4
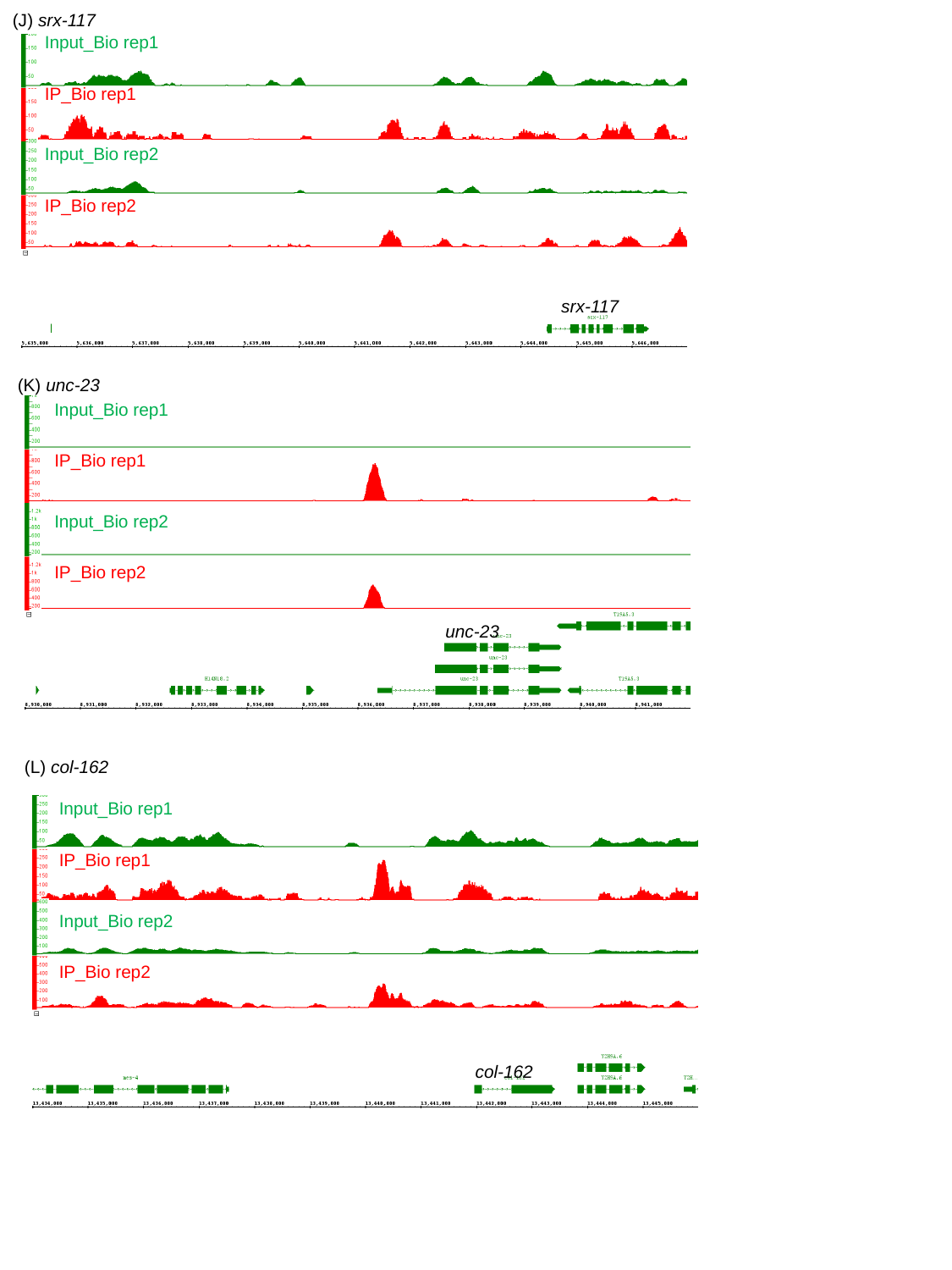

(J) srx-117
Input_Bio rep1
IP_Bio rep1
Input_Bio rep2
IP_Bio rep2
srx-117
(K) unc-23
Input_Bio rep1
IP_Bio rep1
Input_Bio rep2
IP_Bio rep2
unc-23
(L) col-162
Input_Bio rep1
IP_Bio rep1
Input_Bio rep2
IP_Bio rep2
col-162

## Slide 5
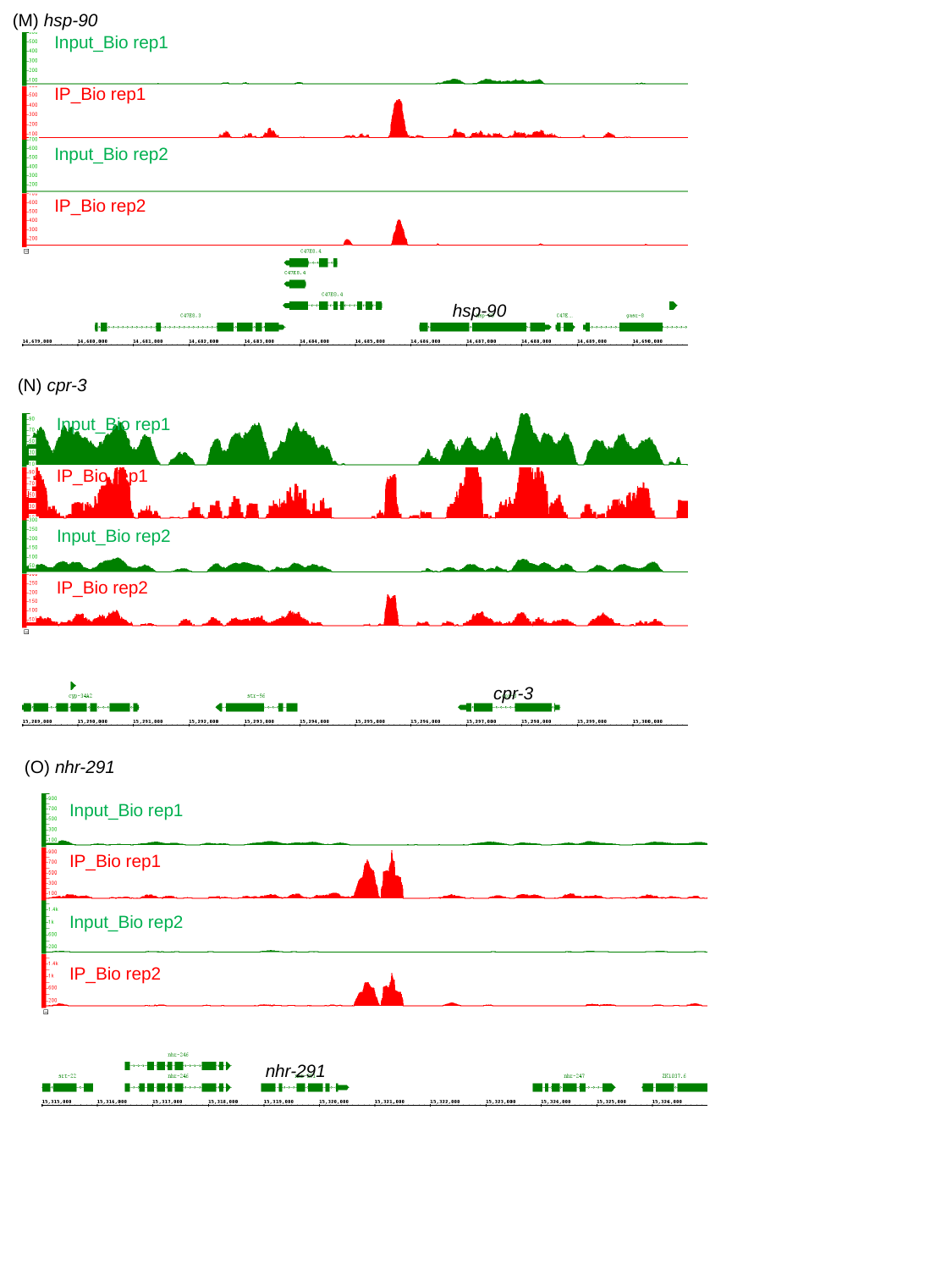

(M) hsp-90
Input_Bio rep1
IP_Bio rep1
Input_Bio rep2
IP_Bio rep2
hsp-90
(N) cpr-3
Input_Bio rep1
IP_Bio rep1
Input_Bio rep2
IP_Bio rep2
cpr-3
(O) nhr-291
Input_Bio rep1
IP_Bio rep1
Input_Bio rep2
IP_Bio rep2
nhr-291

## Slide 6
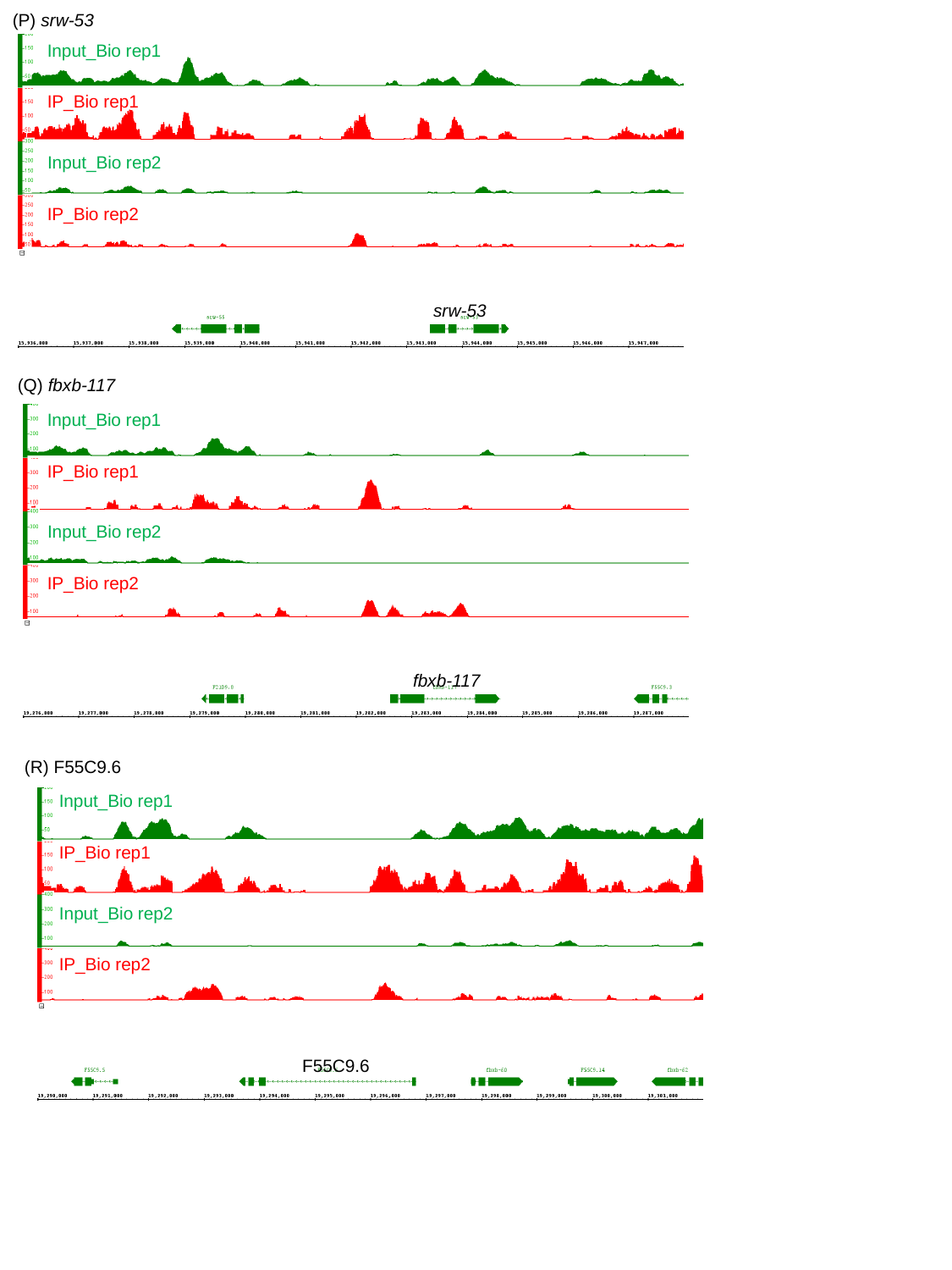

(P) srw-53
Input_Bio rep1
IP_Bio rep1
Input_Bio rep2
IP_Bio rep2
srw-53
(Q) fbxb-117
Input_Bio rep1
IP_Bio rep1
Input_Bio rep2
IP_Bio rep2
fbxb-117
(R) F55C9.6
Input_Bio rep1
IP_Bio rep1
Input_Bio rep2
IP_Bio rep2
F55C9.6

## Slide 7
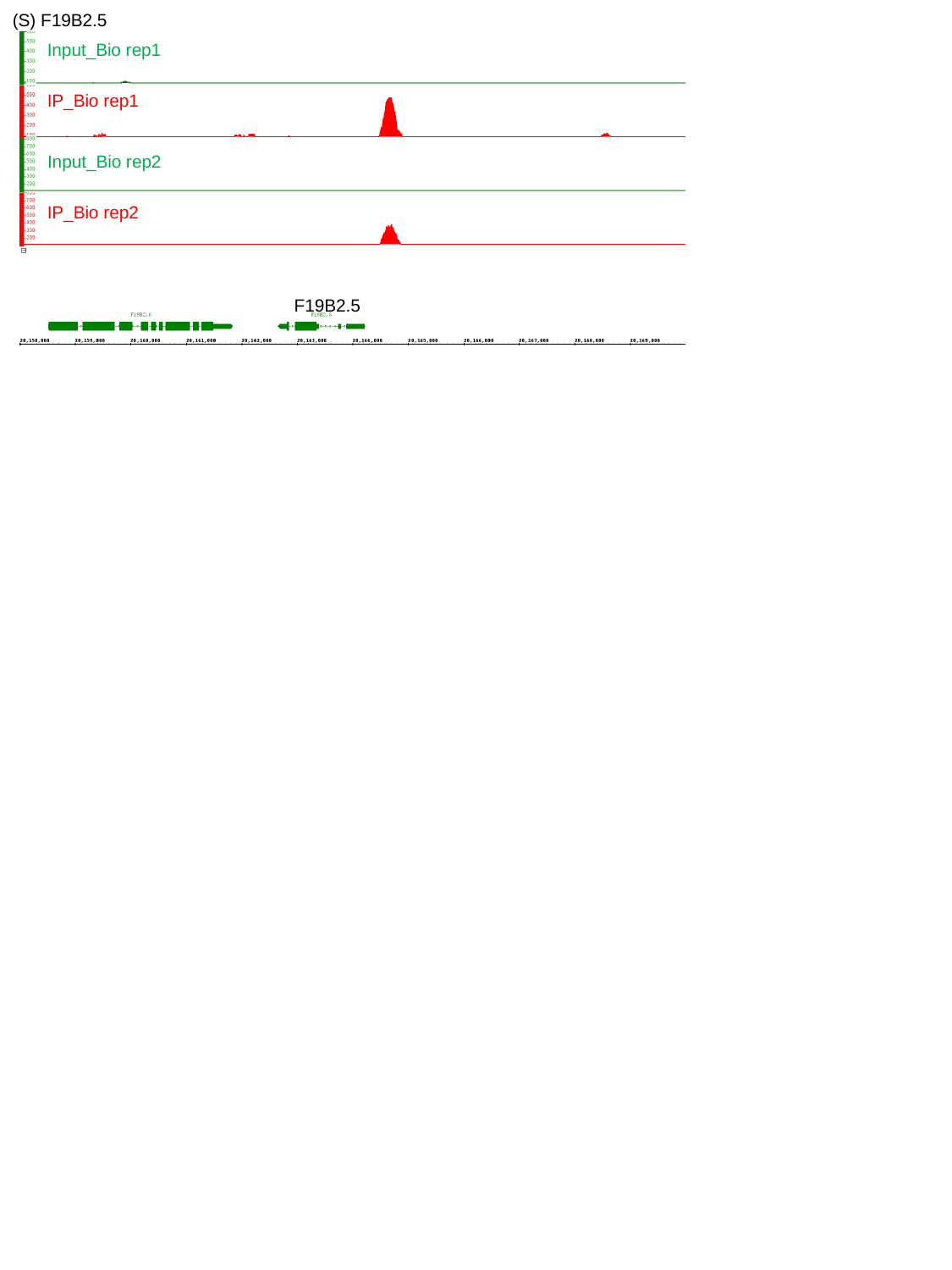

(S) F19B2.5
Input_Bio rep1
IP_Bio rep1
Input_Bio rep2
IP_Bio rep2
F19B2.5
