# Supplementary material for: Whole genome profiling of short-term hypoxia induced genes and identification of HIF-1 binding sites provide insights into HIF-1 function in Caenorhabditis elegans
Source: PLoS One. 2024 May 14;19(5):e0295094. doi: 10.1371/journal.pone.0295094 (PMC11093353; doi:10.1371/journal.pone.0295094)
Supplement: S6 Fig — (PPTX) [file pone.0295094.s006.pptx]

## Slide 1
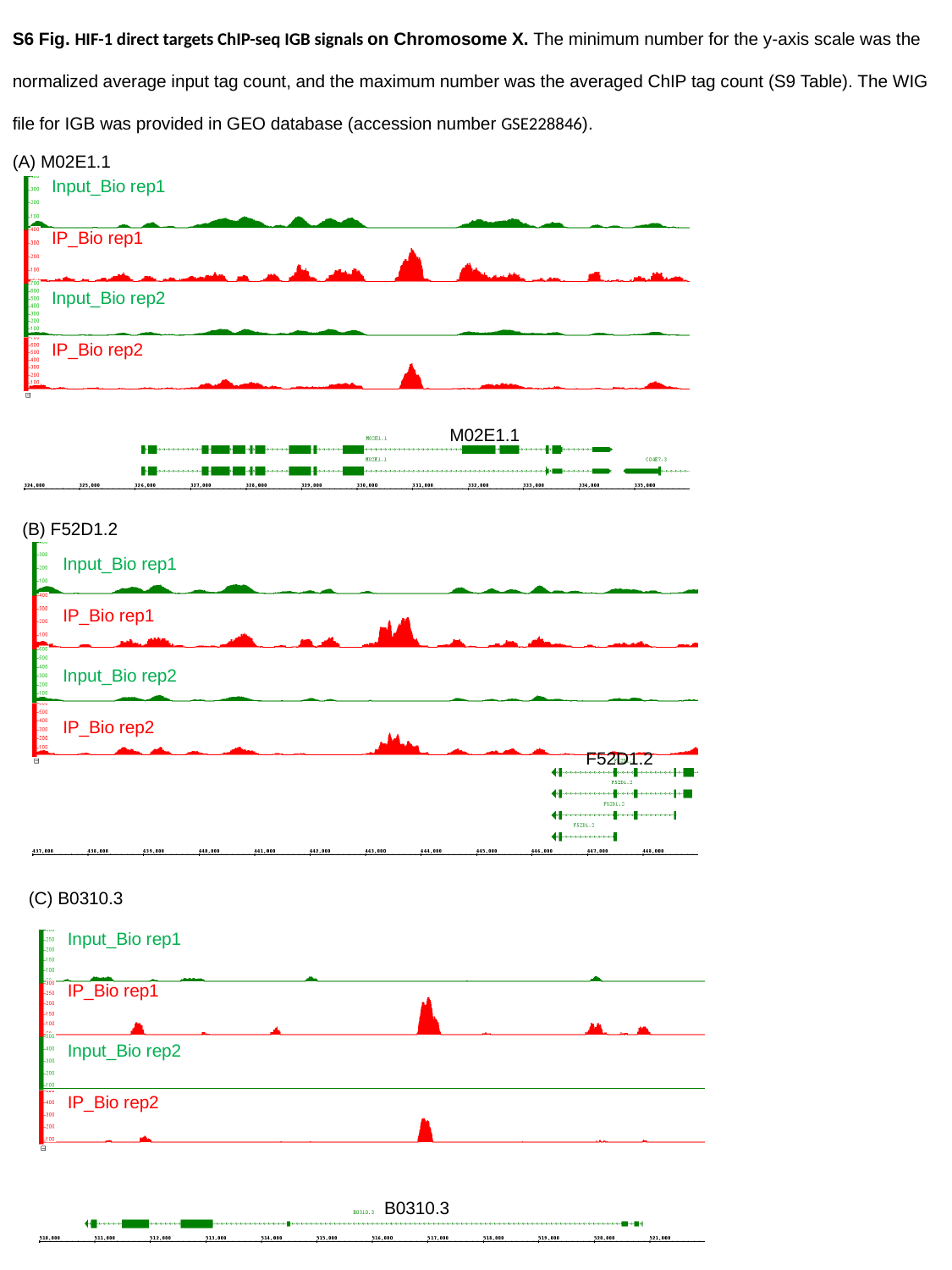

S6 Fig. HIF-1 direct targets ChIP-seq IGB signals on Chromosome X. The minimum number for the y-axis scale was the normalized average input tag count, and the maximum number was the averaged ChIP tag count (S9 Table). The WIG file for IGB was provided in GEO database (accession number GSE228846).
(A) M02E1.1
Input_Bio rep1
IP_Bio rep1
Input_Bio rep2
IP_Bio rep2
M02E1.1
(B) F52D1.2
Input_Bio rep1
IP_Bio rep1
Input_Bio rep2
IP_Bio rep2
F52D1.2
(C) B0310.3
Input_Bio rep1
IP_Bio rep1
Input_Bio rep2
IP_Bio rep2
B0310.3

## Slide 2
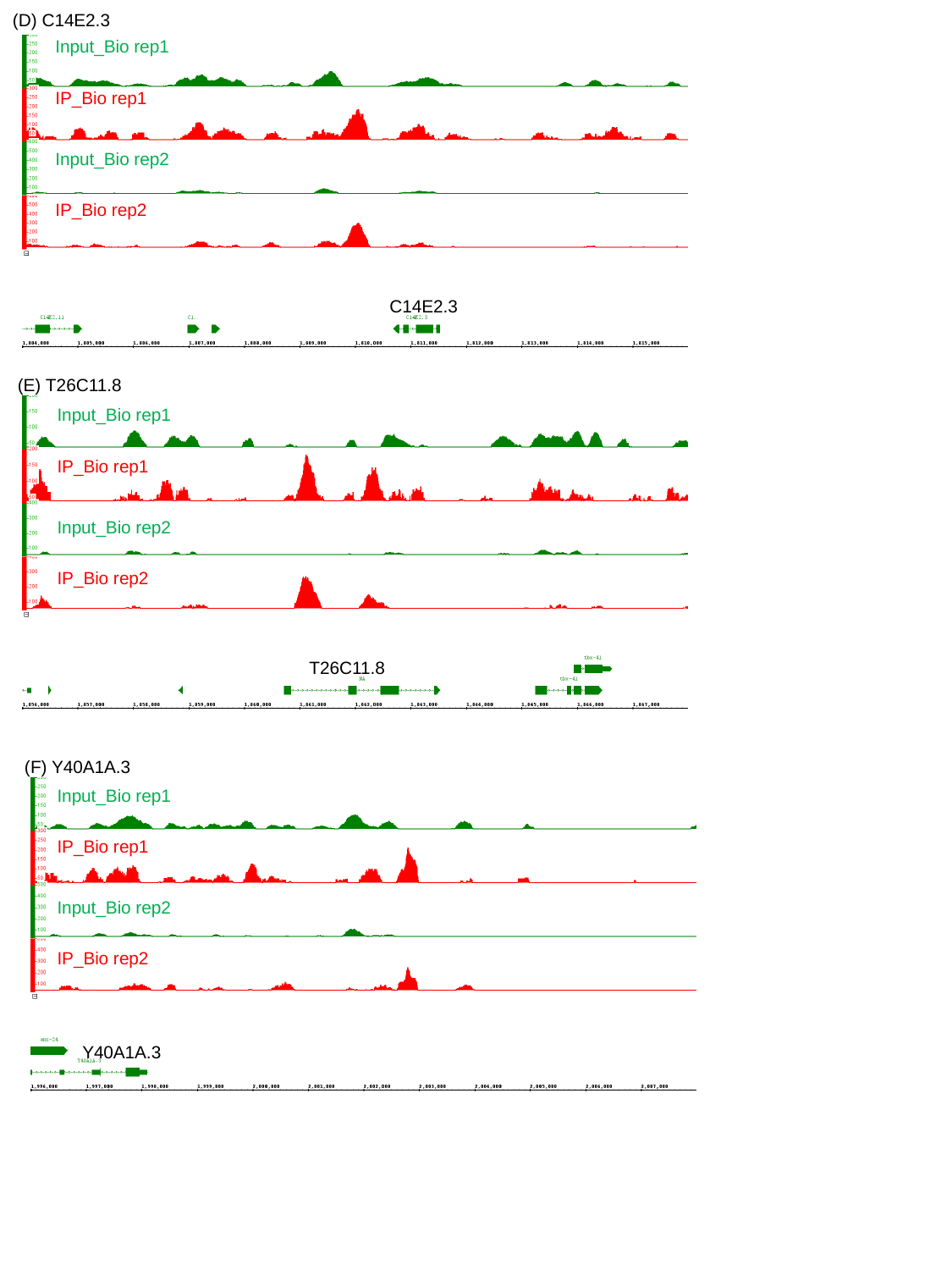

(D) C14E2.3
Input_Bio rep1
IP_Bio rep1
Input_Bio rep2
IP_Bio rep2
C14E2.3
(E) T26C11.8
Input_Bio rep1
IP_Bio rep1
Input_Bio rep2
IP_Bio rep2
T26C11.8
(F) Y40A1A.3
Input_Bio rep1
IP_Bio rep1
Input_Bio rep2
IP_Bio rep2
Y40A1A.3

## Slide 3
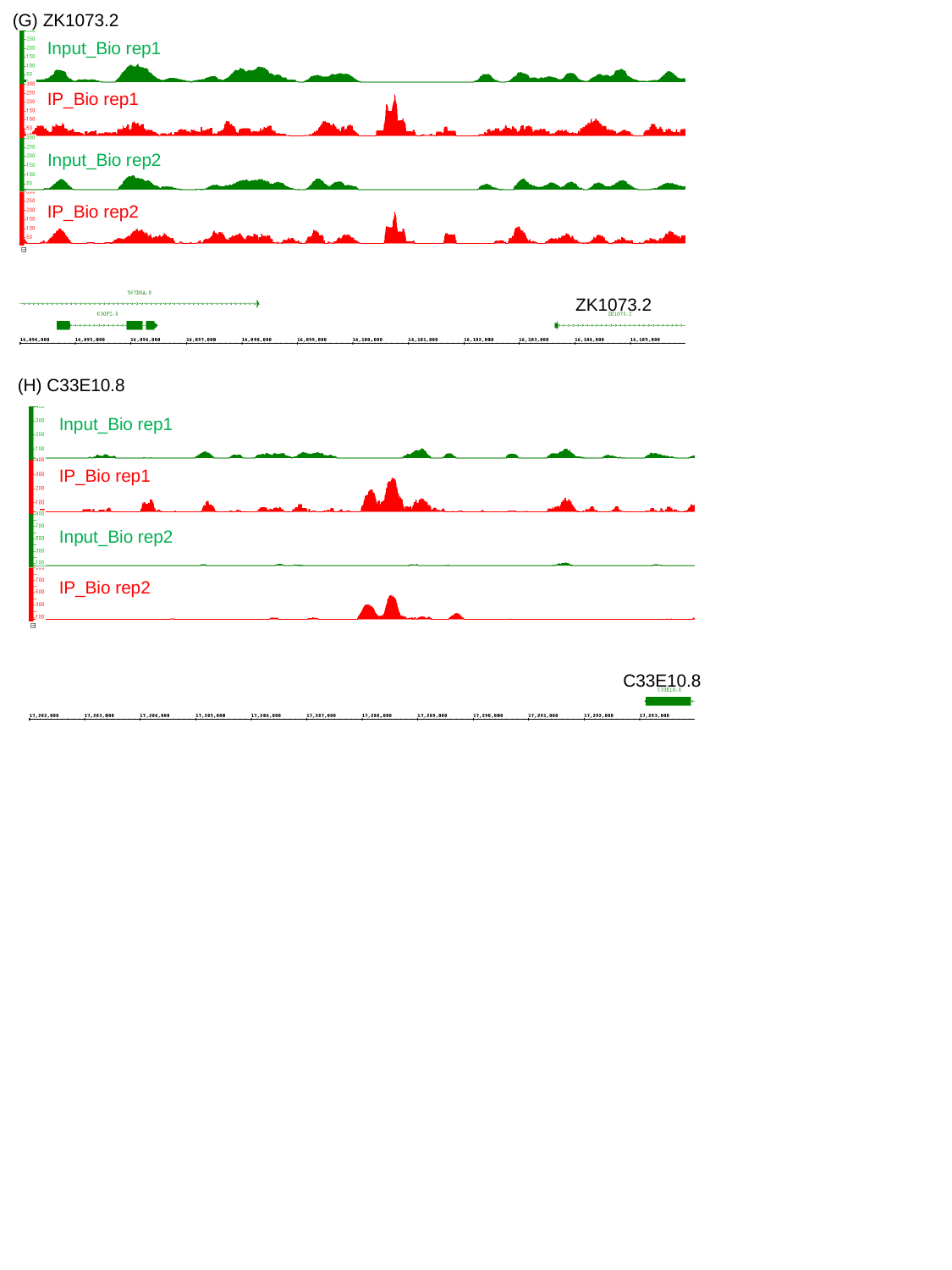

(G) ZK1073.2
Input_Bio rep1
IP_Bio rep1
Input_Bio rep2
IP_Bio rep2
ZK1073.2
(H) C33E10.8
Input_Bio rep1
IP_Bio rep1
Input_Bio rep2
IP_Bio rep2
C33E10.8
