# Supplementary material for: Whole genome profiling of short-term hypoxia induced genes and identification of HIF-1 binding sites provide insights into HIF-1 function in Caenorhabditis elegans
Source: PLoS One. 2024 May 14;19(5):e0295094. doi: 10.1371/journal.pone.0295094 (PMC11093353; doi:10.1371/journal.pone.0295094)
Supplement: S7 Fig — (PPTX) [file pone.0295094.s007.pptx]

## Slide 1
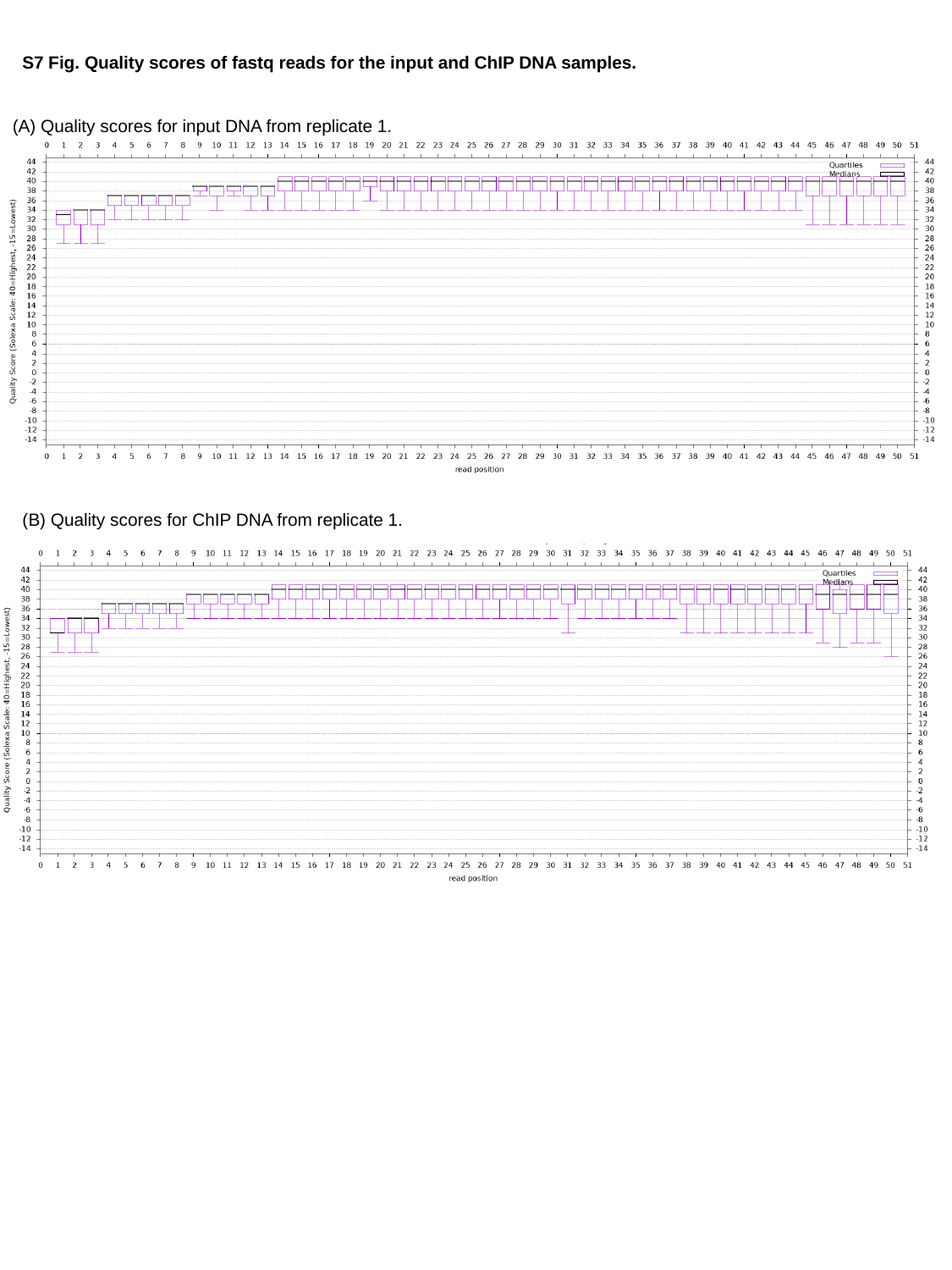

S7 Fig. Quality scores of fastq reads for the input and ChIP DNA samples.
(A) Quality scores for input DNA from replicate 1.
(B) Quality scores for ChIP DNA from replicate 1.

## Slide 2
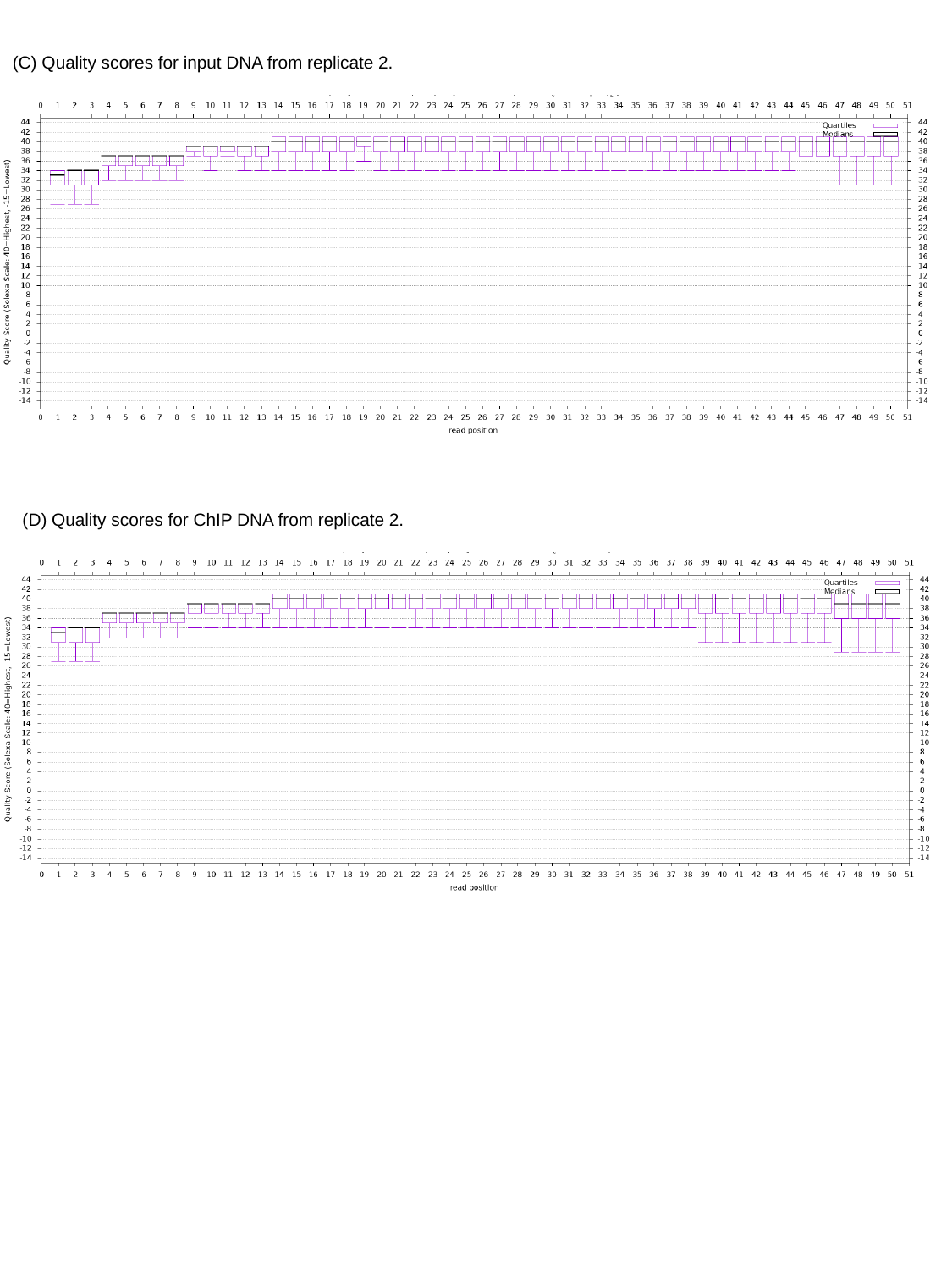

(C) Quality scores for input DNA from replicate 2.
(D) Quality scores for ChIP DNA from replicate 2.
