# Supplementary material for: Whole genome profiling of short-term hypoxia induced genes and identification of HIF-1 binding sites provide insights into HIF-1 function in Caenorhabditis elegans
Source: PLoS One. 2024 May 14;19(5):e0295094. doi: 10.1371/journal.pone.0295094 (PMC11093353; doi:10.1371/journal.pone.0295094)
Supplement: S14 Table — (DOCX) [file pone.0295094.s021.docx]

**S14 Table. Descriptions of mutations used in this study.**

| **Strain** | **Gene** | **Allele** | **Allele description** | **Allele reference** |
| --- | --- | --- | --- | --- |
| ZG31 | *hif-1* | *ia04* | Deletion, loss of function | [[1](#_ENREF_1)] |
| CB5602 | *vhl-1* | *ok161* | Deletion, loss of function | [[2](#_ENREF_2)] |
| RB1297 | *rhy-1* | *ok1402* | Deletion, loss of function | [[3](#_ENREF_3)] |
| JT307 | *egl-9* | *sa307* | Deletion, loss of function | [[4](#_ENREF_4)] |
| ZG1000 ^a^ | *swan-1* | *ok267* | Deletion, loss of function | [[5](#_ENREF_5)] |
|  | *vhl-1* | *ok161* | Deletion, loss of function | [[2](#_ENREF_2)] |
| ZG1001^b^ | *efk-*1 | ok3609 | Deletion, premature stop mutation | This study |
| ZG1002^c^ | *let-363* | *ok3018* | Deletion, loss of function | This study |
|  | *dpy-5* | *e61* | Nonsense substitution | [[6](#_ENREF_6)] |
| RB512 | *mce-1* | *ok243* | Deletion mutation | [[7](#_ENREF_7)] |
| RB1434 | *mmcm-1* | *ok1637* | Deletion mutation | [[8](#_ENREF_8)] |
| RB1700 | *F59F4.1* | *ok2119* | Deletion mutation | This study |
| RB1074 | *smf-3* | *ok1035* | Deletion mutation | [[9](#_ENREF_9)] |
|  | *sqrd-1* | *tm3378* | Deletion mutation | [[10](#_ENREF_10)] |
| RB928 | *phy-2* | *ok802* | Deletion, loss of function | This study |
| RB671 | *fmo-1* | *ok405* | Deletion mutation | This study |
| VC1668 | *fmo-2* | *ok2147* | Deletion mutation | This study |
| RB2535 | *cysl-2* | *ok3516* | Deletion mutation | [[10](#_ENREF_10)] |
| RB2285 | *asns-2* | *ok3108* | Deletion mutation | This study |
| VC107 | *tts-1* | *gk105* | Deletion mutation | [[11](#_ENREF_11)] |
| CF1038 | *daf-16* | *mu86* | Deletion, loss of function | [[12](#_ENREF_12)] |
| GR1307 | *daf-16* | *MgDf50* | Deletion, loss of function | [[13](#_ENREF_13)] |
| ZG372 ^d^ | *hif-1* | *ia04* | Deletion, loss of function | [[1](#_ENREF_1)] |
|  | *daf-16* | *MgDf50* | Deletion, loss of function | [[13](#_ENREF_13)] |
| ZG434 | *hif-1* | *ia04* | Deletion, loss of function | [[1](#_ENREF_1)] |
|  | *egl-9* | *sa307* | Deletion, loss of function | [[4](#_ENREF_4)] |
|  | *iaIS28* | *Phif-1::hif-1::*HA*::*myc | Integrated *hif-1* transgene, 5.2 kb *hif-1* promoter + exon 1 + intron 1 + *hif-1* a exons 2-9 cDNA + HA + myc | [[14](#_ENREF_14)] |

^a^This is *swan-1(ok267);vhl-1(ok161)* double mutant.

^b^This is generated by back cross RB2699*(ok3609)* to N2 four times.

^c^This is generated by cross VC2312(*let-363(ok3018)*) to N2 for once, then cross to CB61(*dpy-5(e61))* for once.

^d^This is *hif-1(ia04);daf-16(MgDf50*) double mutant.

References for S11 Table

1. Jiang H, Guo R, Powell-Coffman JA. The Caenorhabditis elegans hif-1 gene encodes a bHLH-PAS protein that is required for adaptation to hypoxia. Proc Natl Acad Sci U S A. 2001;98(14):7916-21. Epub 2001/06/28. doi: 10.1073/pnas.141234698

141234698 [pii]. PubMed PMID: 11427734; PubMed Central PMCID: PMC35443.

2. Epstein AC, Gleadle JM, McNeill LA, Hewitson KS, O'Rourke J, Mole DR, et al. C. elegans EGL-9 and mammalian homologs define a family of dioxygenases that regulate HIF by prolyl hydroxylation. Cell. 2001;107(1):43-54. Epub 2001/10/12. doi: S0092-8674(01)00507-4 [pii]. PubMed PMID: 11595184.

3. Shen C, Shao Z, Powell-Coffman JA. The Caenorhabditis elegans rhy-1 gene inhibits HIF-1 hypoxia-inducible factor activity in a negative feedback loop that does not include vhl-1. Genetics. 2006;174(3):1205-14. Epub 2006/09/19. doi: genetics.106.063594 [pii]

10.1534/genetics.106.063594. PubMed PMID: 16980385.

4. Darby C, Cosma CL, Thomas JH, Manoil C. Lethal paralysis of Caenorhabditis elegans by Pseudomonas aeruginosa. Proc Natl Acad Sci U S A. 1999;96(26):15202-7. Epub 1999/12/28. PubMed PMID: 10611362.

5. Yang Y, Lu J, Rovnak J, Quackenbush SL, Lundquist EA. SWAN-1, a Caenorhabditis elegans WD repeat protein of the AN11 family, is a negative regulator of Rac GTPase function. Genetics. 2006;174(4):1917-32. Epub 2006/09/19. doi: genetics.106.063115 [pii]

10.1534/genetics.106.063115. PubMed PMID: 16980389; PubMed Central PMCID: PMC1698646.

6. Thacker C, Sheps JA, Rose AM. Caenorhabditis elegans dpy-5 is a cuticle procollagen processed by a proprotein convertase. Cell Mol Life Sci. 2006;63(10):1193-204. Epub 2006/05/02. doi: 10.1007/s00018-006-6012-z. PubMed PMID: 16649143.

7. Kuhnl J, Bobik T, Procter JB, Burmeister C, Hoppner J, Wilde I, et al. Functional analysis of the methylmalonyl-CoA epimerase from Caenorhabditis elegans. FEBS J. 2005;272(6):1465-77. Epub 2005/03/09. doi: EJB4579 [pii]

10.1111/j.1742-4658.2005.04579.x. PubMed PMID: 15752362.

8. Chandler RJ, Aswani V, Tsai MS, Falk M, Wehrli N, Stabler S, et al. Propionyl-CoA and adenosylcobalamin metabolism in Caenorhabditis elegans: evidence for a role of methylmalonyl-CoA epimerase in intermediary metabolism. Mol Genet Metab. 2006;89(1-2):64-73. Epub 2006/07/18. doi: S1096-7192(06)00214-9 [pii]

10.1016/j.ymgme.2006.06.001. PubMed PMID: 16843692; PubMed Central PMCID: PMC2761207.

9. Romney SJ, Newman BS, Thacker C, Leibold EA. HIF-1 regulates iron homeostasis in Caenorhabditis elegans by activation and inhibition of genes involved in iron uptake and storage. PLoS Genet. 2011;7(12):e1002394. Epub 2011/12/24. doi: 10.1371/journal.pgen.1002394

PGENETICS-D-11-01251 [pii]. PubMed PMID: 22194696; PubMed Central PMCID: PMC3240588.

10. Budde MW, Roth MB. The response of Caenorhabditis elegans to hydrogen sulfide and hydrogen cyanide. Genetics. 2011;189(2):521-32. Epub 2011/08/16. doi: genetics.111.129841 [pii]

10.1534/genetics.111.129841. PubMed PMID: 21840852; PubMed Central PMCID: PMC3189795.

11. O'Rourke D, Baban D, Demidova M, Mott R, Hodgkin J. Genomic clusters, putative pathogen recognition molecules, and antimicrobial genes are induced by infection of C. elegans with M. nematophilum. Genome Res. 2006;16(8):1005-16. Epub 2006/07/01. doi: gr.50823006 [pii]

10.1101/gr.50823006. PubMed PMID: 16809667; PubMed Central PMCID: PMC1524860.

12. Murphy CT, McCarroll SA, Bargmann CI, Fraser A, Kamath RS, Ahringer J, et al. Genes that act downstream of DAF-16 to influence the lifespan of Caenorhabditis elegans. Nature. 2003;424(6946):277-83. Epub 2003/07/08. doi: 10.1038/nature01789

nature01789 [pii]. PubMed PMID: 12845331.

13. Ogg S, Paradis S, Gottlieb S, Patterson GI, Lee L, Tissenbaum HA, et al. The Fork head transcription factor DAF-16 transduces insulin-like metabolic and longevity signals in C. elegans. Nature. 1997;389(6654):994-9. Epub 1997/11/14. doi: 10.1038/40194. PubMed PMID: 9353126.

14. Zhang Y, Shao Z, Zhai Z, Shen C, Powell-Coffman JA. The HIF-1 hypoxia-inducible factor modulates lifespan in C. elegans. PLoS One. 2009;4(7):e6348. Epub 2009/07/28. doi: 10.1371/journal.pone.0006348. PubMed PMID: 19633713.
