# Supplementary material for: Whole genome profiling of short-term hypoxia induced genes and identification of HIF-1 binding sites provide insights into HIF-1 function in Caenorhabditis elegans
Source: PLoS One. 2024 May 14;19(5):e0295094. doi: 10.1371/journal.pone.0295094 (PMC11093353; doi:10.1371/journal.pone.0295094)
Supplement: S1 File — (DOCX) [file pone.0295094.s022.docx]

**S1 File. Sequences co-immunoprecipitated with HIF-1** **on chromosome 1.** The HRE similar sites were color coded as red on the reference Watson strand, and as green on the Crick strand.

>chrI:223000-223399_Y48G1BL.8

ATTTTCTAAGAATAATTTTGACGGGAAATTCAAATTTGTTGAGAAACTCGAGTAAATGCTGGAATGTTCTAGAACCTTCCTGGAGATTGGGGAAAATTTTAGTATGTTCTAGAACCATCGGCGAACTTAAGAAAATTCTGGAAAGTTCTAAAACCTTCTAGAAAATTCGTGAAAATTCTGGATTGTTCTAGAAATTTCTAGAAAATTCGAGAAAACTCTGGAACGTTCTATAGAACCTTCTAGAAAATTCGGGAAAATTCTGGAATGTTCTAGAAAATCTTGTTTGCCAAAAGCTCTCGGAAGGAACCGGAAAAGTTTCTTATGATTTGAAGAACGTCTAGAAACGTTCAAGAAATTTAGAAGTAGTTCCAGGAAACCTGGTATTTTTAAGTTGTAACTC

>chrI:334600-335399_*daf-25*

TTAAATGTTTTTCGAGATTAAAATAAACCGAAAACCCAAAAAAAATGTAAAAATTGTGTTCTTTTTGTTATAATAAACCAGAATTTTCTCGAAATTTTCAGAAGGTTCTAGAATATTTCAGAATTTTCTCGAAATTTCCAAAAGGTTCTAGAACATTTCAGAATTTTCTCGAAATTTTCAGAAGGTTCTAGAACATTCCAGAATTTTCTCGAAATTTTCAGAAGGTTCTAGAATAGTTCAGAATTTTCTCGAAATTTCCAAAAGGTTCTAGAACATTACATAATTTTCTCGAAATTTCCAGAAGGTTCTAGAACATTCCAGAATTTTCTCGAAATTTTCAGAAGGTTCTAGAATATTTCAGAATTTTCTCGAAATTTCCAAAAGGTTCTAGAACATTTCAGAATTTTCTCGAAATTTTCAGAAGGTTCTAGAATATTTCAGAATTTTCTCGAAATTTCCAAAAGGTTCTAGAACATTACAGAATTTTCTCGAAATTTCCAAAAGGTTCTAGAACATTACAGAATTTTCTCGAAATTTTCAGAAGGTTCTAGAATATTTCAGAATTTTCTCGAAATTTCCAAAAGGTTCTAGAACAATCCAGAATAATGTTTTCAAAAAATTCAAATTTGAATTCCCGCCAAAATGTTTTCAAAAAATTAAAATTCGAATTTCCCGCCAAAATATGTACAGTACTCCTACAGTACCTCTACAGTACTACTACAGTACCCCGACCATATCCCACTACTAACCCCAAACCTATATCTCTTCAAAAGACTAAAACACAATTTTTCCTAAACTAC

>chrI:778000-778599_T06A4.1

AAAACACTTTGGCGGGAATTCAAAATGTTATTTCTTAACAACTTCCTGAAATGCTCTAGAACCTTCTGGAATATTTGAGAAAACTCTAGAATGTTCTAGAACCTTCTGAAAAATTCGAAAAAAGTCTAGAATGTTCTAGAGCCTTTTGGAAAATTCGAAAAAAATCTGGAATATTCTAGAACCTTTTGGAAATTTTGAGAAAATTCTGGAATGTTTTGGAACCTTCTGGAAAATTCGAGAAAATTCTGGAATGTTCTAGAACCTTCTGAAAAATTTGAGAAAATTCTGGAATGTTCTAGACCCTTCTGGAAATCCGAGAAAATTCTGGAATGTTCTGGAACCTTCTGGGAAATTTTTAGAAAAATCCTGGAATTCTCTAGGACCTTCTGGAAAATTTGAGAAAATTCTTGTCGCCAAAGTTTTGTGAAAAAATTTAGCTGGAAACTAAATAATTTTGTGAGAATTCAAACTTTAATTTTTCCAATTTTTTCGGATTTTTTTTTTAGCTTTTAAGCTTTTTACATTTTCTATAAATTTTAGATTTCAAAAAAAAATTGGCGAAAAATTTTGACCAAATTTTTTGGCTTTATAGCATAATTT

>chrI:780600-781199_T06A4.1 TACGAGGCAGTAGGCATCAGGGCTTTGTGGCAACCGGCAACCGGCAACCAAAAACCAGGTTGCCGGTTACCGAAAAATTGCCGAAATTTCAGCAACCAAAAGTCGCCAGAATTTTCTCGAATTTTCCAAAAGGTTCTAGAACATTCCAGAATTTTCTCGAATTTTCCAAAAAGTTCTAGAACATTCCAAACTTTTCTCAAAGTTTCCAAAAGACTCTAGAACATTCCAGAATATTCTCGAATTTTTCAGAAGGTTCTAGAACATTCCTGAATTTTCTCGAATTTTCCAAAAGGTTCTAGAACATTTCAGAATTTTCTCAAATTTTCCAAAAGGTTCTAGAACATTCCAGAATTTTCTCGAATTTCCCAAAAGATTCTAGAACATTCCAGAATATTCTCGAATTTTTCAGAAGGCTCTAGAACATTCCAGAATTTTCTCGAATTTTCCAAAAAGTTCTAGAACAAGTTGCAGAAATTTTCAGCGTACGGCAACTTCAGCAATTGCGGGTTGGCATGTAGGCAGGCAGGCATGTTAGTAGGCAGTTTTGACAGTTTTGAAGTTAACAATCCTACCTGATGCACAAGAAAATGCGCGGCAATT

>chrI:1715600-1715999_*atg-5*

CCATAGTTTGTAGTCTATGTAGTCTTTGTAGTGTGCAAAGTTATGGCTTATGGAGGGATATTGATTTTATGTTAGTTGTGGGATATGGTCGGGGTACTGTAGTAAAACTGTAGAGGTACTGTAGGGTTACTGTAGGATTACTGTAGTTTGGGAAAAATTGATTTCAGATCTTTCTGGACAGTTCAAGAACCTTCTGGGATTTTCTGGAAAATTCCGGAATGTTCTAGAATTTTCTGAAAAATTCGAGAAAACTTTGGAATGTTCCAGAATCTTATTAAAAATTCGAGAAAATTCTGGAATGTTCGTGGCGAGACCTATCCGTTTTTCGATTCATCCAAATGGTCGGAAAACAATTATTTTTTGAAGAATTTTGAAATTTGGAATGGCCTAGAAGTGTTTT

>chrI:2639600-2639999_F40E3.3

TTTTCGAGAATTTATTTTTAATATTTTTTCAACTTTTTTGGTTAAAAATTGTCTTCCAAAATTTGCAAATTCAGTGCGAAACGATCGATTTTCCATATAAGAATATTAGGAGGCAAGAAAAACTAAATTTGAATGCGCGCGCAAAAGGTGTTCAACAGGGCGCATTTGCTCTCCGAGCTTTCCGGGCGCCACGGTAGCACAGAAACTAGATCTCTCGTAAAATTTGAGAAAGATCTCGCAGGTACGCAGCGAAATGGTCCGCAATGTGTCTCGCGGTGTTTGCGTACTTGCGTACCGTAGTCCGCAAAACATTGCAGCGGCAAATAGATTTTTGAAGCAAATTTTAGCAGAAAAAAGGCAGAATTACTAGTTTAAAGTGAATAAAATTAAAAAAAAAAGA

>chrI:4573000-4573999_*col-52*

CTACGACATGCATGTTTCAAAAATTCACAGACAGTATGAAAACTTTCTGTTACTTTTTGACCAAGACAGTAACCTTACAATACCACTACAGTACCTTGACATTATCCTCCACCGACTCCTAACCCAATATCTCTTCAAAGGACAAAAAGTCAAATTTTCCAAAACTACAGTAACCCTACCGTATACCTACAGTACCCCTATAGTACCACTACAGTACCTTGACTTGATCCCCCATCAACTCCCAAATAACTACCTCTTCTAAAGCTCTAAGCTCAATTTTTCGGAACATTCCAGAATTTTCTCGATTTTTCTAGAAAGTTCTGGAACATTCCAGAATTTTCTCGATTTTTCTAGAAAGTTCTGGAGCATTCCAGAATTTTCTCGATTTTTCTAGAAAGTTCTGGAACATTCCAGAATTTTCCCGATTTTTCTAGAAAGTTCTGGAACATTCCAGAATTTTCTCGATTTTTCTAGAAAGTTCTGGAACATTCCAGAATTTTCCCGATTTTTCTAGAAAGTTCTGGAACATTCCAGAATTTTCTCGATTTTTCTAGAAAGTTCTGGAACATTCCAGAATTTTCCCGATTTTTCTAGAAAGTTCTGGAACATTCCAGAAATTTCCCGATTTTTCTAGAAAGTTCTGGAACATTCCAGAATTTTCCCGATTTTTCTAGAAAGTTCTGGAACATTCCAGAATTTTCTCGATTTTCTAGAAAGTTCTGGAACATTCCAGAAATTTCCCGATTTTTCTAGAAAGTTCTGGAACATTCCAGAATTTTCCCGATTTTTCTAGAAAGTTCTGGAACATTCCAGAATTTTCTCGATTTTTCTAGAAAGTTCTGGAACATTCTTTTTTCCCGCCAAAAAATTTTTCTCAGAAAATTTAAATTTCCCGCCAAAATATTTTTCACAGAAAATTTAAATTTCCCTCCAAAATATTTTTCTCAGAAAATTTAAATTTCCCGCCAAAATATTTTTCACAGAAAATTTAAATTTCCCG

>chrI:4769400-4769799_M04F3.3/*kin-35*

GAAATGCGATAGAATTTGTCTGTGAAAAACCAAATATCGATTTTCCTTGAATATCGTGAAGAACAAATCTTCATACTTACGATTCTTCACAAATTCGATGAAAATCTGATAGTTTTTTCAATTTTAGCTCTATAATTCCGAAAAAAATCTTCTGTTAATTTTCGCGAAATGTCGTCAATTAAAATGCGCGCTGTGCAGTACGCAACGGTGTACGAAAGTTTACACTGAGTATTCAACGTTGAGCACGCAGTCAGCCAAATTGGAATACGGTAGAGAATCCTCGTCCATGTACCCCCCGCCGGGCCGTGGGTCGAGCTGAATACACGTGATTCTCCTACGCAAGGACTATAAAAGTGAGCTGTCACTCTCATTTTTCTTCTTTTTTCATAAAATTTAGTTC

>chrI:5379600-5380199_ *pqn-44*/*tent-5*

GCAACTTTTCAAAACTAAAAAATTTGGTATCCTGCCAAAACTTTAACTGAAAATTCAGAAAAATCTAATGGCGTTTAGGATATTATTTATATGAATGGAAAAAATTGCGTTCAACTTGCAAATTATGACTTACCCTTCTTCTTCTATAAGTTGTCATAGCTAGAGTTGTTGATTGAATTTTTGAAGTAGCTATTTTCGGATTCAAAAAAGAATTTCTCCAGAATTCTAAGCTTCTTTTATACTCTATTCATTCTCAATTCCTACATCATTTCGAGAAACTTCTCGAACCCTAATACACTCGTTGTTTGTTTGTTTGTCTGCGTCTCTTCCTCTTCTCCCACTGAGAGAGTAAACTGCTTCCCCCAGCGGCGCGGAAGATTCCGGAATATTCTGGCAATTCTCCTGATTTCTTGATCTCAAAATTTGGAGGAAGGAAGAAAAAAAACTAAAACAAAAAAACCTTATTTCTAATATATCAAATTGTCTCTTTTCTGTTACCTAATCTGTCAACTGAATCTGTTTCGTCTCCTAATTTTTTAGATTTTTCTTCATCTCGTCGAATGCCATTGTCGTCTTTCAAAGGTCATTCAACAAAAGGCG

>chrI:9322400-9322799_*hsp-70*

TACATACCTTTCCATTTTGATAAATACCGACACATGAGTACGTAGTACCCAAGTCGATTCCAATCGCTTTGCATGTAGACATATTCAGTTAATTTCAGCGTTTGAATAGCAGAAGTTAAAAATTCTTGTTTGGTTAAAATTCTGCATAAGATTTGAGTTTTTATACGTTCCTCTGGCATCTTCTAGAACCTTCTACAATTTACTAGATGCAAGGGGTGCTCCATCCTTTAGTTCTTCCACTGCCTGAGTCTCTGTGGGTCGCCTATGCTATTTTCTCTGTTCTCTTTTGCAAGAACGTTCTTGTGGCTGTTTTTTGGTTAGTGCATCACAGATGTTTACGCCAGGGAAAAGGAAAGAAATATCTAGAATAGGTCTTTGTTGTTGACGTTTCTAGTGGGGA

>chrI:11763200-11763799_*vab-10*

AGTACTGTAGGAGTACTGTAGGATTACTGTATTTTTGAAAAAAATTGGCTTTTCGTCTTTTGAAGTGATATTGGTTTGAGGTTAGTGGTGGGATATGGTTGGGGTACTGTAGTTGTACTGTAGAGGTACTGTAGGAGTACTGTAGGATTACTGTAGTTTGGGAAAAATTGACTTTTCGTCTATTGAACGGATATTGGAAACTTTGAGAAAATTCCGGAATGCTCCAGAACCTTCTGGAAAATTCGAGAAAAGTCTGTAATGTTCCAGAACTTTCTAGAAAAATCGGGAAAATTCTCGAATGTTCCAGAATTTTCTAGAAAATTCGAGAAAAGTCTGGAATGTTCAATTCCCTGAAAAAATTTAAGAAACTTCTGCGAAGTTCTAGAACCTTTCAAGTTTTGATACTCCTAGAATATTCTTAAGTTTTAAGAATTAAAAAAAACCAGAAATTGTTATAGATAATTTCCAAAAACGCTCGCCAAAAACTTTCGGAAAGGACCGGAAAAAGTCTCATAAAAGTTCCAGGAAACTTGAGGTTTTAAAATTGTAATTGCTTAAAATTAAGTATATAATATTTATGTTGCTCGAAAACTTCCGGAA

>chrI:13230800-13231799_ZK1053.2

GGGGTACTGTAGTAGTACTGTAGGAGTACTGTAGGAGTACGGTAGGATTACTGTAGTTAATGAATAATTGTGTTTTTGTCTTTTGAAGAGATATAGGTTTGGGGTTAGTAGTGGGATATGGTCGGGGTACTGTACTGTAGGAGTACTGTAGGAGTACTATAGGATTACTGTAGTTTGGGAAAAATTGACTTTCCGTCTTTGGAAGGGAAATTGGAAACTTCGGGAAAATTCTGAAATGTTCCAGAACTTTCTAGAAAAATCGAGAAAATTCTGAAATGTTCCAGAACTTTCTAGAAAAATCGAGAAAATTCTGAAATGTTCCAGAACTTTCTAGAAAAATCGAGAAAATTCTGAAATGTTCCAGAACTTTCTAGAAAAATTGAGAAAATTCTGAAATGTTCCAGAACTTTCTAGAAAAATCGAGAAAATTCTGGAATGTTCCAGAACTTTCTAGAAAAATAGAGAAAATTCTGGAAGTGTATGTGTGCCAAATTCTGAAATGTTCCAGAACTTTCTAGAAAAATTGAGAAAATTCTGAAATGTTCCAGAACTTTCTAGAAAAATCGAGAAAATTCTGGAATGTTCCAGAACTTTCTAGAAAAATCGAGAAAATTCTGGAAGTGTATGTGTGCCAAATTCTGAAATGTTCCAGAACTTTCTAGAAAAATTGAGAAAATTCTGAAATGTTCCAGAACTTTCTAGAAAAATCGAGAAAATTCTGGAATGTTCCAGAACTTTCTAGAAAAATCGAGAAAATTCTGGAATGTTCCAGAACCTTCTAGAAAAATCGAGAAAATTCTGAAATGTTCCAGAACTTTCTAGAAAAATCGAGAAAATTCTGGAATGTTCCAGAACTTTCTAGAAAAATCGAGAAAATTCTGGAAGTGTATGTGTGCCAAATACTTTTAATGGTGCCAGTCGTTGCCCGCGCCGTAGGAGCGGTCAGCGGCTGGTTTTTAATTAAAAAATTTGAAATAAAAACTTCAAGGAATAATAACAT

>chrI:14033200-14034399_*ins-30*

AGTGAGCAATTGGTGTAAAATTAAGTGATAATTATCGGCAGTGGTCCTTTTCACTTAGTGTTTTTAACTATAATTTTGGCGGGAATTCAAATTTTAATTTTTGAAAACACTCTGAAAAATTCTAGAACTTTTTGGAAAGTTTTAGAAAATTCTGGAATATTCTACAACATTCTGGAAAAGTCGGTAAAATTTTGGAGTGTTCCCGAACTTTCTGGAAAATTTTAGAAAATTCGGGAATGTTCTGGAACCTTCTTGAAAATTCGAGGAAATTCTGGAATGTTCTAGAACCTTCTGGAAAATTCGATAAGAATCTGGAGTGTTCCAGAACTTTCTGGAAAATTCGATAAAATTCTGGAATGCTCTGGAACTTTCTGGAAAATTCCAGAAAATTCCGGAATGCTCTAGAACCTACTGGAAAATTTGATAAAATTCTGGAATATTCTACAACATTCTGCAAAAGTCGGTAAAATTCTGGAATGTTCTAGAACTTTCTGGAAAATTCCAGAAAATTCCGGAATGCTCTAGAACCTTCTGGAAAATTCGCTAAAATTCTGGAATATTCTACAACATTCTGGAAAAGTCGGTAAAATTCTGGAATGCTCTGGAACTTTCAGGAAAATTTTAGAAAATCCTGGAATGTTCTGGAACCTTCTGGAAAATTCGCAAAAATTCTGGAGCGTTCTAGAACCTACTGGAAAATTTGATAAAATTCTGGCATATTCTACAACATTCTGCAAAAGTCGGTAAAATTCTGGAATGTTCTAGAACTTTCTTGAAAATTCCAGAAAATTCCGGAATGCTCTAGAACCTTCTGGAAAATTCGGTAAAATTCTGGAATATTGTACAACATTCTGGAAAAGTCGGAAAAATTTTGGAGTGTTCCCGAACTTTCTGGAAAATTTTAGAAAATTCTGGAATGTTATGGAACCTTCTGGAAAATTCAATAAATTTCTGGAATGTTCCAGAACCTTCTGGAAAATTCGGTGAAATTCTGGAATGTTCTGGAACTTTCTGGAAAATTTGATAAAATTCTGGAATGTTCTGGAACCTTCTGGACAACTCGGTAAACGAAACCATTTTCTTATAACCCCCTCCTTGACTTTTTCAGGGTATTATGCCATCTGACGTTGCTCAGAAACTGGTAATTAAGTGATTATAGGTGATAATTATTCTATGTGAAAATGTACTTTCTGATCAAAT

>chrI:14668400-14668999_*ero-1*

CATCAAAATAAAGTTGGCACTTTATCGATGAAATCGCATTTTCACTTTCCATGGTATACGATTCAGAAAACTCACAGAGACAATTAGAATTTTCTGGTTTTTCTTCCAACACATCGCCAAAATTTTGGGTCTCACCACGACGGGTCTCACCACGATGGGTCTCGCCACGATGGGTCTCTCCACGATGGGTCTTTCCACGATGGGTCTCACCACGATGGGTCTCGCCACGAAGATCTCGCAGCAACATTTTTTTTAAATTTTCCAGAAGGTTCTAGAACAATCCAGAATTTTTTCGAATTTTCCAGAAGGTTCTAGAACAATCCAGAATTTTTTCGAATTTTCCAGAAGGTTTTGGAACATTCCAGAATTTTCTCGATTGTTCCAGAAGGTTCTAAAGCTTTTCAGCATTTTCCAGAAGGTTCTGGAACATTCTAGAATTTTCCAGAAATTCCCAGAAGGTTCTGGAACATTTCAGAATTTTCCCGAAGTTTCCAATTTCCCTTCCAAAGACAAAAACACAATTTTTCCCAAACTACAGTAATCCTACCCTACTCCTACAGTACTCCTACAGTACTACTACAGTACCCCACCCATATCCCC

>chrI:14686200-14687399_Y105E8B.9

ACTACTTTTTCGGAAAAAAACATTTTTTTTGGCAAAATGGCATTTTTTGGCCTTTTGTTTTATCACAACTTTTTGCCTTTTGCACTTATGAACTCAAACTTTCTTTCAAAAAATCCACCTCTCTGAGTAGTATCTTGCACATAAATTTGGAACAAAACCGAGCAAAACCCGAATTTTAATTCAATTAAAACATGCTTTTTTGGGGGTAAAAAGAGCAACAAAAATTTTGGGCAACGACTGGCACCGTTTAAAGTATTTGACACACATACACTTCCAGAATTTTCTCGATTTTTCTAGAAAGTTCTGGAACATTCCAGAACTTTTTCGAAATTTCTAGAAAGTTCTGGAGCATTCCAGAATTTTCTCGATTTTTCTAGAAAGTTCTGGAACGTTCTAGAATTTTCTCGATTTTTCTAGAAAGTTCTGGAACATTCCAGAATTTTCCCGATTTTTCTAGAAAGTTCTGGAACGTTCTAGAATTTTCTCGATTTTTCTAGAAAGTTCTGGAACGTTCTAGAATTTTCTCGATTTTTCTAGAAAGTTCTGGAACATTCCAGAACTTTTTCGAAATTTCTAGAAAGTTCTGGAGCATTCCAGAATTTTCTCGATTTTTCTAGAAAGTTCTGGCACATTCCAGCATTTTTTCGAAATATCTAGAAAGTTCTGGAACATTCCAGTATTTTCCCGATTTTTCTAGAAAGTTCTAGAACGTTCTTGAATTTTCTCGATTTTTCTAGAAAGTTCTGGAACGTTCTCTTAATTTTCTCGATTTTTCTAGAAAGTTCTGGAACATTCCAGAATTTTTTCGAAATTTCTAGAAAGTTCTGGAACATTCCAGAATTTTCCCGATTTTTCTAGAAAGTTCTGGAACGTTCTAGAATTTTCTCGATTTTTCTAGAAAGTTCTGGAACATTCCAGAATTTTCCCGATTTTTCTAGAAAGTTCTGGAACGTTCTAGAATTTTCTCGATTTTTCTAGAAAGTTCTGGAACATTCCAGAATTTTTTCGAAATTTCTAGAAAGTTCTGGAGCATTCCAGAATTTTCTCGATTTTTCTAGAAAGTTCTGGAACATTCCAGAATTTTCTCGATTTTTCTAGAAAGTTCTGGAACATTCCAGAATTTTCCCGATTTTTCTAGAAAGTTAGAGAAAGTTTCTAACCCCAAACTAATATCCCTCCAACAGCCGAAAACGCCTTGCC

>chrI:14756600-14756999_*dnj-12*

CGTTTGAAAAAACATCAAAACTGCAGTTCATTCATGAATGAAAAAAAAACAGATTTTTAAAAAGATTTTAAAATTACAGTATCTCCCCAATTGCTATCCTGTATAATAGTTGTTTTGAATGAAAATCTATGTAAGATTTTTTTTAGTTTTCCACAAATAATCGTGCATTTTTAAAATATTTCTCACGAATCAGTTTCCACGAGACAAAAATGTCCCCGCTTGGTGAGTTGCAAGTGCGCTCCACCGATAAAAGTGTCGAGAATGTTCACGAAAAATCGTTAGAACAGTTTTCGCGTTTTCTCCTCTTTTTCGCCCTTTATCTCTTGACTTTCCCCATTTTTGACTTCAAAATCGATTTTATCGTGTTGGATTTACGCAAAACAATTCGCTTTTTGGTATA
