# Supplementary material for: Whole genome profiling of short-term hypoxia induced genes and identification of HIF-1 binding sites provide insights into HIF-1 function in Caenorhabditis elegans
Source: PLoS One. 2024 May 14;19(5):e0295094. doi: 10.1371/journal.pone.0295094 (PMC11093353; doi:10.1371/journal.pone.0295094)
Supplement: S2 File — (DOCX) [file pone.0295094.s023.docx]

**S2 File. Sequences co-immunoprecipitated with HIF-1** **on chromosome 2.** The HRE similar sites were color coded as red on the reference Watson strand, and as green on the Crick strand.

>chrII:301400-301799_K01A2.5

TGTTTCATAGTTCCTTTTTATTAGAAACTACACATTTATATTACAAATAACTAGATGTTTCGGTGTTTTAGTGGTTATGTTTTTTTTAATAATTTTTATTACCTTTCTAGAATCTTCCAAAATTTTCTCGAATTATTCTGAAGGTTCCAGAACCTTCCAAAATTTTCTCGTATTTTCCAGATAGTTCTAGAACATTCCGGAATTCTATCTAATTTTTCAGAAGGTTTTAGAACATTCCAAAACTTTCTCGTATTTTCCAGATGGTTCTAGAACTTTCCGCAGCTTACAGGCACAAAATTAATCCGGTCTTGTCCAAATTCCTTCTGAAATCTTAATCTGTAGGCTACAAATTTCTCATTCCTATCGGACTTTCAAAATCTTTTTCGTAGAACTATTCGTC

>chrII:451200-451599_W10D9.1

TGGCGAGAGTTTCTGGTAATTATACCAATTTTTGATCTTTTTAAAAAATTCTGAAAACTTCAGAATATTCTAGTAGTATCAAAACTTGAAAAAGTTTTTTTAAACTTTCCGAAAGGTTCTAAAACATTGCAGAAGTTTCCTGAATTTTCAAAACGAAATTTGACGTTTCAGAATTTTCTCCAATTTTTTTAGAAGAATTTCCTAGAAAGTACTGGAACATATCAGAATTTTCTCAAATTTCCTAGAAAGTTCTGGAACATTCCAGACTTTTCCCGATTTTCAAGAAAGTTCTCGAACATTCCTGAGTTTTCTCGAATTTCATAGAAAGTTCTGGAACATTTCAGAACTTTCCAAAATTTTCCAGAGAGTTCTGTTTTCTAGACGTCGCGCCAAAATACTT

>chrII:2328000-2328599_ZK1240.8

ACATTTTCCCACTAGCGCTAACTTTTGCTCGTCTCAGTTTCTTTTGAAAAAATTCGAAATTTTCATATTCTACCATGTTTTCCTTATTAAAAAGAACATTCAACTATGTGTTTTCAGTTGAAAACAGTAGTGTTTTTTTAAAATTTGCTAGAAGATTCTAGAACATTCCAGAATTTTCTATAATTTTTTCAGAAGGTTCGAGAACATTCCAGAATTTTTTTTGAATTTTTTAGAAGGATCTAGAACATTACAGAGATTTTTTTCTAGAAGTTTCCACGTTTTTTTTAAAATTTTCTTAGATGTTCTAGAACATTCGGGAATTTTTTTTTAATTGCTAGAAGTTTCTAAAACATTCCAGAATTTTCCCAAATTTTCAAGAAGGTTCTAGAACATTCCAGAATTTTTTTGAATTTTCTAGAATGTTCTAAAACATTCCAAAATTTTCTCAAACCTTCCAGAAGGTTCTAGATCATTTCAGAATTTTCTTGAATTTTCCAGAATATTCTAAAACATTTCAGAATTTTCGAAATTTTCATATCCTACCATGTTTTCCTTATCAAAAAGAACATTCAACTATGTGTTTTCAGTTGAAAACAGTAG

>chrII:2330000-2330999_ZK1240.1

TTTTAGAAACTTCTAGCAATTAAAAAAAATTCCCGAATGGTCTAGAACTTTCTGGAAAATTCAAGAAAATTCTGGAATGTACTAGAACTGTCTGGATAATTCGGGAAAATTCTGAAATGTTTTAGAATATTCTGGAAAATTCAAAAAAACTCTGAAATGATCTAGAACCTTCTGGAAGGTTTGAAAAAATTCTGGAATGTTTTAGAACATTCTAGAAAATTTGGGAAAATTCTGGAATGTTTTAGAAACTTCTAGCAATTAAAAAAATTCCCGAATGTTTTAGAAACTTCTAGCAATTAAAAAAAAATTCCCGAATGTTCTAGAACATCTAAGAAAATTCAAAAAAAAACCTGGAAACTTCTAGAAAAAAATCTCCGGAATGTTCTAGATCCTTCTTAAAAATTCCAAAAAAATTCTGGAATGTTCTCGAACCTTCTGAAAAAATTATAAAAAATTCTGGAATGTTCTAGAACCTTCTAGCAAATTTCAAAAAATTCTAGAATGGTCTAGAACCTTCTGTAAATTTTGAGAAAATTCTGGAATGTTTTAGAAACTTCTAGCAATTAAAAAAAATTCCCGAATGGTCTAGAACCTTCTGGAAAATACAAGAAAATTCTGGAATATTCTAGAACCTTCTGGAAAATTCGAGAAAATTCTGGAATGTTCTAGAACCTTCTGGAAAATTTTAGAAAATTCTGGAATGTTCTAGAACCTTCTGGAAAATTCGAGAAAATTCTGTAATGCTCTAGATCATTCTGGAAAATTTTAGAAAATTTTGGAATGTTCTAGAACCTTCTAGCAATTTTCAAAAAAACTCTAGAATGATCTAGAACCTTCTGGGAAATTTAAGAAAAATTCTGGAATCTTCGAGAACCTTCCTTAATTCTCCTTCCAGGACCCCAACTGCTCCTCCGACGAAAAGCTAATGTGCCGAACTTGCGAGGAGTTTGGAGTTCACGCCGGACACTCGAAGGGCCTCCTACAGACGGAAGCTTTGAAA

>chrII:2348000-2348399_F43C11.4

TATTCTAGAAATTTCAGAAAATTTTAGAACGCTCCACGAACTTCTTTTCTTTTGACTTTTCCAGAAGATCATTCCAGAGTTTTGTTGAATTTTTTAGAAAGTTCTGGAACATTTCAGACAATTCTTGAATTTTCTATAGCGATTTAGAACATTCCAGAATTTTCCCGATTTTTCTAGAAAGTTCTGGAATATTCCAGAATTTTCTCGATTTTTCTAGAAAGTTCTGGAACGTTCCAGAATTTTTTCGAAAATTCCAGAATTTTAGAATTTTCAGAAAGTTTGAATTTCCCACCAAAACATTTTTTTCAGAAAATTTAAATATCCCACCAAAATATTTTTCACTAAAAATTTGAATTTCCCGCCAATTTTTTTCACTAAAAATTTGAATTTCCCGCCAATT

>chrII:4047800-4048399_K07D4.2

TTTGGAATGAAAATTGTGACATCGATATTCAATTTTCTTTAGTTTTTTTTTTCGTATTATCGATTAATCCATTATTTCCGTAAATTGTCACCAGCACTACAGTAGTCATTTAAAGAATTACTATAGTTTTCGCTAAGAAATATTTTGCGCGTCAAATATGTTGCGCAATATTATCAAAAAATATAAATCGCACTGGCCACCACGGCCCGGCGAACCGTACGTGGCTGTAAAATCTCTACCGTAGTAAAATTTAGCTGACTGAATATTCAACGTTTGATACTCAGCGAAAAGTTTCATACGACATTTTGCGGACGCGGCATTTTAATTGACGACACTTTCTTAGTTATAGAAACGCTGGTTATTTCGGCAATTTTCGGGCAGAAATTGTGAAAAATAAATGAATTTTTATCGAATTTACAAAAAATAATAATTTTAAAGGATTGTATGTTGCTGATATTGAAGAAAATCGATACTTTAAACAGTTTTTCGCAATGTTTCTCGATTTTGGCCAATTTTTTCGAGTTTTAGAGCCCTGAAATTCCCTAAATCGTATAATCTCACATTTGATTATTGAAAAACGCGTGTTATTATTAAAAATTC

>chrII:8459800-8460599_*zyg-9*

AATACCACTACAGTACCTTGACATTATCCTCCACCGACTCCTAACCCAATACCTCTTCAAAGGACGAAATGCCAATTTTTCCAAAACTACAGTAACCCTACCGTATACCTACAGTACCCCTATAGTAACACTACAGTACCTTGACTTGATCCCCCATAAACTCCCGAATAACTACCTCTTCTAAAGCTCTAAGCTCAATTTTTCGGAACATTCCAGAATTTTCTCGATTTTTCTAGAAAGTTCTGGAACATTACAGAAATTTCTCGATTTTTCTAGAAAGTTCTGGAACATTCCAAAAATTTCCCAATTTTTCTAGAAAGTTCTGGAACATTACAGAAATTTCTCAATTTTTCTAGAAAGTTCTGGAACATTCCAGACTTTTCCCAATTTTTCTAGAAAGTTCTGGAACATTCCAGACTTTTCCCAATTTTTCTAGAAAGTTCTGGAACATTACAGAAATTTCTCAATTTTTCTAGAAAGTTCTGGAACATTCCAGACTTTTCCCAATTTTTCTAGAAAGTTCTGGAACATTCCAGACTTTTCCCAATTTTTCTAGAAAGTTCTGGAACATTCCAGAATTTTCTCGATTTTTCTACAAAGTTCTGGAACATTCCAGAATTTTCTCGATTTTTCTAGAAAGTTCTGGAACATTCGAGAATTTTTTCGAAATTTCCAGAAGATTCTAGATTTCCAGAATTTTAGAATTTTCAGAAAATTTAAATTTCCCGCAAAAATATTTTTCACAGAAAACTTAAATTTCTCGCCGAAATATTTTTCTCAGAAAATTTAAATTTCCCGCC

>chrII:9631600-9633199_ZK1307.7

AAAGGAACTTTTTCTCATAAATTAGGCTTTTAGTCTAAATGTTTTTCTTATCTGAAATATTTCAATAACAAGGTCACATTCATTAAAGTTGGTAAACACATGATTACCAACTTGTAAAATTATGCCTGAAATTTGTTTAAAAGTAAATTAATACACTCTTTCTTCCTGATTTCTACCGTTTCTTATTTTAAAAACCAGCCGCTGACCGCGCCTACGGCGCGGGCAACGACTGGCACCATTAAAAGTATTTGACACACATACACTTCCAGAATTTTCTCGATTTTTCTAGAAAGTTCTGGAACATTCCAGAATTTTCTCGATTTTTCTAGAAAGTTCTGGAACATTCCAGAATTTTCCCGATTTATCTAGAAAGTTCTGGAACATTCCAGAATTTTTCCGATTTTTCTAGAAAGTTCTGGAACATTCCAGAATTTTCTCGATTATTCTAGAAATTTCTGGAACATTCCAGAATTTTCCCGATTTATCTAGAAAGTTCTGGAACATTCCAGAATTTCCTCGATTTTTCTAGAAAGTTCTGGAACATTCCAGAATTTTCTCGATTTTTCTAGAAAGTTCTGGAACATTCCAGAATTTTCTCGATTTTTCTAGAAAGTTCTGGAACATTCCAGAATTTTCCCGATTTATCTAGAAAGTTCTGGAACATTCCAGAATTTTCTCGATTTTTCTAGAAAGTTCTGGAACATTCCAGAATTTTCCCGATTTTTCTAGAAAGTTCTGGAACATTCCAGAATTTTCTCGATTATTCTAGAAATTTCTGGAACATTCCAGAATTTTCCCGATTTATCTAGAAAGTTCTGGAACATTCCAGAATTTCCTCGATTTTTCTAGAAAGTTCTGGAACATTCCAGAATTTTCCCGATTTTTCTAGAAAGTTCTGGAACATTCCAGAATTTCCTCGATTTTTCTAGAAAGTTCTGGAACATTCCAGAATTTTCCCGATTTATCTAGAAAGTTCTGGAACATTCCAGATTTTTCTCGATTTTTCTAGAAAGTTCTGGAACATTCCAGAATTTTCTCGATTTTTCTAGAAAGTTCTGGAACATTCCAGAATTTTCTCGATTTTTCTAGAAAGTTCTGGAACATTCCAGAATTTTCCCGATTTTTCTAGAAAGTTCTGGAACATTCCAGAATTTTCTCGATTTTTCTAGAAAATTCTGGAACATTCCAGAATTTTCTCGATTTTTCTAGAAAGTTCTGGAACATTCCAGAATTTTCTCGATTTTTCTAGAAAGTTCTGGAACATTCCAGAATTTTCCCGATTTTTCTAGAAAGTTCTGGAACATTCCAGAATTTTCTCGATTTTTCTAGAAAGTTCTGGAACATTCCAGAATTTTCCCGATTTATCTAGAAAGTTCTGGAACATTCCAGAGTTTTCCCGATTTTTCTAGAAAGTTCTGGAACATTCCAGAATTTTCTCGATTTTTCTAGAAAGTTCTGGAACATTCCAGAATTTTCCCGATTTTTCTAGAAAGTTCTGGAACATTCCAGAATTTTCCCGATTTTTCTAGAAAGTTCTGGAACATTCCAGAATTTTCCCGATTCTAGATTTCCAGAATTTTAGAATTTTCAGAAAATTCAA

>chrII:11545200-11545799_F54D5.12/*dhgd-1*

ATAAGCAAGTGAATTTTAAGCATAACTTTTCTTCCTAGTGTACGTAAATCATTCTTTCCAACAAACATATTTTTTCGTGACGAAACTTCGCCTTCCAGAATATTCTTTTTTCAGAAAATAAATACCAAAAAGCACAATTTCTTATCTCTTGCTCATTCTTTTCTTTGTATCGTGCTCATGCTTTTATTCATTCCTCATTTTTATCTTGCGAAACCAATGTATTTTCAATAAAAAAAACGAGTGATGCATGTGCGCTCCACCGGCCGACGGAAGATCGAACATGCACTGCGCTTCGCGAGTAAATAGAACGCTCTGGAAAGTTCCGCACTCTTCTCTCTCATGATTCGGCGCACTCTCTCTTCCATTTCTCCGTGTTTCCTCTTCTGATGTTGACCCATATTTATTCTGCCGGGTGTATTCTTTTTATCTATCTGTTGCTTCATTTATTCCGTTAACCTGTTACTGGTTAATATTTCAAAAATTCATATGATTTCTTTTCAGATTACTTTCCACAATGGGAAAAGACTACTACAAAGTGCTGGGCATCAGCAAAGGTGCAACAGACGATGAAATCAAAAAGGCTTATCGTAAAATGGCTTT

>chrII:11759000-11759799_F44E5.5_F44E5.4

CAATCTTTCCGTATCTGTGAATGCCACGTATGATGGAGTTGTCTTATTTCCTTCCGAGTTGGCGAGGATCTCAACCTGAAAATTAAACAAATTAGATATTGAAAGTATTATGTTTAAAATATTAACTAAAATCTACAATTTTAACAAAATGTTATTAAAATTGAAAATGCCAAAAAATACTTACCTTTCCATTCTGATAGATTCCAACACACGAGTACGTAGTACCGAGGTCGATACCAATAGCTTTGCATGAAGACATTATTTGTAGTTTGTAGTAGTAGAACAGTAGAACTTTTAGTTTCTTCTGAGGGTGACATACTACCCGAATCCCAGCCCTTTTATACCTTTCCCGTAACTTCTTCTAGAAGCTTCTGGAATATTCCACTGCACCAGCTGCATTGTCTCTCCTCCTTCTTTCTGCTGCCCAGCCATTCGAGAACGTTCTAGAACCTTCCATTATAAGAGAAAGAGACGGCCGACAGAAGAGACAACACAGCTGGCAACAGAGACGCAGATTGTTGGAGGAAATGGAATGTTCGAGAAAAAGAAAGAGGGAGACAGAGTGATGCAGCTGGTGCAGTGGAATATTCCAGAAGCTTCTAGAAGATGTTACGGGAAAGGTATAAAAGGGCTGGGATTCGGGTAGTATGTCACCCTCAGAAGAAACTAAAAGTTCTACTGTTCTACTACTACAAACTACAAATAATGTCTTCATGCAAAGCTATTGGTATCGACCTCGGTACTACGTACTCGTGTGTTGGAATCTATCAGAATGGAAAGGTAAGTATTTTTTGGCATTT

>chrII:12677800-12678199_Y38E10A.22

TTTACTGAAAAAAAACGACATTTTTGGGATTATTTTGAAGAAAAACGTGATAAAAAGCTTTTTAAAAAGCGAGATTTTCGATTTTCCCGCTGAAAAATCGACAGAAAATTACGGTTTTTATGTTAAAATGAACCTAATTTTTCATATTTAACGATTTCAAAAGAATTTTTCAAGAATTCCAGAAAGATTCCGCAAAAACATGCTGAAAACATTCGAGAAAAATCGGGAAATTAATTCAAAAAATGAGTGATGGCCTAGAATTGTTATCAATGGAGCGTGGTTGTTGCAGTTGGGAAGTTAGTCCCTGACAATTTGATTTAATTATGATTGATTTTAAAAGAGAAAAACAGGCCGAAATTCAAGAATTCGAGGTAAACCTGAATGCAGAAAGACAAATTCA

>chrII:14405000-14405599_*nurf-1*

TTACCGACTTTTACATTTTTCGTGGTGATAATATGATTTAAGACAGCTCGATACGTTAAAATTTGAACTTTTTGGGAAGATAAGCTTTAGTAGTGATATATGTCCATCTTCGCCGATCACATCCATTCATCACGTGGACTCCGGGTGTCTGTACCACGAAGCATAATTGAATGAGTCACGCGACCTCAGATTTGAATATAGACTCCGCCCACCTTTTGACGAGAGACTACACTGCTGTGAAGAGGCGAAAAGAATGACATCATAGAACACCCTTTCGATTGTATATGCCGTCTCCCTCTCCTCGGGTCAACACATCTGCGCGTATTCAGCCCGATTGCTCGTACATTCGTCGAAGAGCATGGTAAATCGAGAGACGCAGACGGCGTAGAAACGAGAGAGACGCGGAAAGAAGCGCGCAGAATGTTCGAGAAACGCGGAGAAGAAAACTGGTGGTCGAGGCCCCCCGCGCGGGAGAATATATAAGTGTGCCAGGAAGCTATTCCCAACTAGATTTCTAGATCCTATCCATCTGAAAATTTGAAAAAATGGTTCTCCGAACTGTTATTCTGCCTACTGCAACCTACACTGCACCATCACAAT

>chrII:14561600-14561999_*mog-4*

CTGGAAAGTTGGATAATTACAGGAAAATTGAAATTATTAAGTGATGAATAATGATTTTTCAACAAAATTTTAGTTGAAAACCACGAAAAAATTCGAAACTCCTTGAGAAACTCATAAAAATCTATGAAAAACAAAAATAAATAAATAAAAATTATTTTTCGAAAGCGCCGGCAATTGTGTGCAAACACCGTCACGTCGGATTGCAGACCCGAGGAATTTCGGATACACGGGGAGGCAAAGCTAACGTGGCTGAAGAAATTTCTACAGTAGTCCCATTTGGCTGACTGAATATTCAACGCGAATAAGTTTTGTACACTATTGCGTACTCTGCGTACGCGCATTTTATTTGACGACAATTCGTTAATATCAGCTCTGGCTAAAAGCGCTTTTCTCATTATTT

>chrII:15000800-15001199_Y53F4B.10

GTGAGACCTATCGTGGTGAGACCCGTCGTGGTGAGACCCACCGTGGTAAGACCCAAAATTTTGGCGGGTAATTTAAATATTCGGAGAAAAATATTTTGGCGGGAAATTTAAATTTTCTGTGAAAAATATTTTGGCGGGTAATTTAAATTTTCGGAGAAAAATATTTTGGCGGGAAATTTAAATTTTCTGTGAAAAATATTTTGGCGGGTAATTTAAATTTTCTGAGAAAAATATGTTGGCGGGAAATTTAAATTTTCTGAAAATTCTAAAATTCTGGAAATCTAGAATCTTCTGGAAATTTCGAAAAAATTCTCGAATGTTCCAGAACTTTCTAGAAAAATCGGGAAAATTCTGGAATGTTCCAGAACTTTCTAGAAAAATCGGGAAAAGTCTGGAATGT
