# Supplementary material for: Whole genome profiling of short-term hypoxia induced genes and identification of HIF-1 binding sites provide insights into HIF-1 function in Caenorhabditis elegans
Source: PLoS One. 2024 May 14;19(5):e0295094. doi: 10.1371/journal.pone.0295094 (PMC11093353; doi:10.1371/journal.pone.0295094)
Supplement: S3 File — (DOCX) [file pone.0295094.s024.docx]

**S3 File. Sequences co-immunoprecipitated with HIF-1** **on chromosome 3.** The HRE similar sites were color coded as red on the reference Watson strand, and as green on the Crick strand.

>chrIII:3456200-3456599_C34C12.2

TAGATCTACAAAAAATGCGGTAGTTTAGACTTTCTCTGATTTTTTTTAAAATGAAAACTGACAATTTTCACAATTTCTGTTCTACAATTTTCAAAAAACCCAGTTTGAAAACATTTTTTCAATTTTTACGAATTGTCGTCAATTAAAATGTGTTGTTTTTTTTTTCACTGACTACAGTACCCAGTATGTGAATTACCGTCACGGTGTCTGCAGACTTTTTGCGCGATGCAAGCGCGCTCCACCGCACGTGTGTAAAACAAAAACAATCGGCTGCTGAAAACGGTTTTGAGTCGTTTTTTCGCTAAATTTCCATTCGATTTTCTATTTTTCTGCTGAGATCTGCATTTTCCCAAGTAAAAACAATTTATTTTAGTTGAGAAATCACCAAAAACGTGCAAAG

>chrIII:3897000-3897999_*tir-1*

TGGGAAATTTAAATTTTCTGAGAAAAATATTTTGGCGGGAAATTTAAATTTTCTGAAAATTCTAAAATTCTGGAAATCTAGAATCTTCTGGAAATTTCGAAAAAATTCTCGAATGTTCCAGAACTTTCTAGAAAAATCGGGAAAATTCTGGAATGTTCCAGAACTTTCTAGAAAAATCGAGAAAATTCTGGAATGTTCCAGAACTTTCTAGAAAAATCGAGAAAATTCTGGAATGTTCCAGAACTTTCTAGAAAAATCGAGAAAATTCTGGAATGTTCCAGAACTTTCTAGAAAAATCGAGAAAATTCTGGAACGTTCCAGAACTTTCTAGAAAAATCGGGAAAATTCTGGAATGTTCCAGAACTTTCTAGAAAAATCGAGAAAATTCTGGAACGTTCCAGAACTTTCTAAAAAAATCGAGAAAATTCTGGAATGTTCCAGAACTTTCTAGAAAAATCGAGAAAATTCTGGAATGTTCCAGAACTTTCTAGAAAAATCGAGAAAATTCTGGAACGTTCCAGAACTTTCTTGAAAAATCGAAAAAATTCTCGAATGTTCCAGAACTTTCTAGAAAAATTGGGAAATTTCTGGAATGTTCCAGAACTTTCTAGAAAAATCGAGAAAATTCTGGAACGTTCCAGAACTTTCTAGAAAAATCGAGAAAATTCTGGAACGTTCCAGAACTTTCTAGAAAAATCGGGAAAATTCTGGAAGTGTATGTGTGTCAAATACTTTTAATGGTGCCAGTCGTTGCCCGCGCCGTAGGCGCGGTCAGCGGCTGGTTACGATATAATATGTTTATTAAATTCAAAAAATAGGTTCCACTTCCTCTGAATCTTTTTTTCCACCTGATAAATGTTTAATAAAGTTCATAACTAGAGAAGTTCGACTACTCAAAGCCAAGAGAGACGCAGACATATCGAGAGAAAAGTGGATAAGGAGAGCGAGGCTCAAGTAGAAGAAAAAACGTGAAAACGAATAGGGAGCCTGGCCTGGAAAT

>chrIII:6170000-6170399_*efk-1*

CATCTGGTTGAGTAGATTCTTGGAATATAAAAATGAAATATTCTGATTTCCTTGAAACTTCAATCTGACCGAGCCGAATGTAATAGGTCAAATGTGACCAATATCTCTCAGACATTTATCACTAGCACTTTAAAATAGAAACAGGAATCAGTAGACTCTCTGAAGTTGAAAAAAGACCGACGGAAAAACTGGGAGAAAACTACGTGAAGAAATGGAAAATCGAGAAAGGCCTAGAGGCACCCAGAAGAATGGAGAAAGGAGAAGAAGGAGAGACAATGATGAAGAATGAGAGATTTAGAGAAACAGACAGAGTGTAAACCGAAAAGAGATGGGTGTGCACATTTGACTTGGATGGATGAATGGTTGGGACCACTTGGTGTTTTTTTTGGCTGAAAAATAT

>chrIII:7854400-7854799_*rpl-35*

AAGCGTATGAGTAACTTTTCACAGTTTAAATTCAATTTTACTGAAAACTTTATTTACAGAAACTTGCAAAATTAGCGTTTTTGTGCAGATGTTTTATCGATGGCAATTTAAGGCATTTTGGAACACTTTTGAACTGTTCCAAACAAAAAAGCAGGTTGTAAACCTGCAAAAAAATTATGATTCCGTCAAAGCGATAACATTTTTCGGAAATTCCGAAAAAGTTTGATGTGAAAAACTTCCTTTCAAACCGCCGCGCTCATCATACACCTAGGAGCGTGTTTTAGTGAAGTGTATCAGAAGTGTGCGAGACTTGCGAAAGTGGGCCTCGACCACGCCCCCTTTTTCGTGCGCTATGTGTTTCACTGGGAAAATTCCGATTTTTTTCGTTTTTTGCCTCTTT

>chrIII:8344200-8344799_ZC262.7

ACCTGCTTGTGAACCTTCGTCAACTTGTCCAAATTGTTCTCCAAGAACTGAATTCGTTGCTTTTGAGCTGGTCCACTGAGAGAATCTTCTCCCTCGGTGTCTTGATTGGCTTGGCATCGAGCAACAAGTACTCTCATGAACTCCTTTTTCAAATTTAGGGAAAATTCTGGAATGTTCCAGAACTTTCTAGAAAAATTGGGAAAAGTCTGGAATGTTCCAGAACTTTCTAGAAAAATTGGGAAAAGTCTGGAATGTTCCAGAACTTTCTAGAAAAATTGAGAAAAGTCTGGAATGTTCCAGAACTTTCTAGAAAAATTGGGAAAATTCTGGAATGTTCCAAAACTTTCTAGAAAAATTGGGAAAAGTCTGGAATGTTCCAGAACTTTCTAGAAAAATTGGGAAAAGTCTGGAATGTTCCAGAACTTTCTAGAAAAATTGGGAAAAGTCTGGAATGTTCCAGAACTTTCTAGAAAAATTGGGAAAAGTCTGGAAAAAAAGAGGTATTGGGTTAGGAGTCGGTGGAGGATAATGTCAAGGTACTGTAGTGGTATTGTAAGGTTACTGTCTTGGTCAAAAAGTAACAGAAAGTTTTCATACTGT

>chrIII:8446800-8447399_*hsp-110*

ATAGTCATTGGTGATAACTTCAATGCCTCCTTGACGTGCAACGCCAATGTAGCAGTTGAGGTTACCGATGTCGAATCCAAGAACCGACATTATTCTTTATAGCAGGAGCGCTGAAATTAAAGATAAAAGTTTAGACAGTTTAGACCAGCTAAAAACGGACAAAATGGGTTGAAGAAGCTTTGAAAGTATATTTATACAAAAACCAAATATTTTTCTAGAAATTTCGAGCAAATAAAAAAAAAGTGAGCAAGAAAACAAAGGTGAAACAGCGAAAAACGACCGAAAAGGGCGTGTCTCAGGCTGACTCCCTTATAATGGTGTTTAAAGGCACACCGAATATTCGAGAAACGTCTAGTACATCGTTGCGAGACCCATGTAAAATTCTCTACAGAGCAGACTTGCATTATTTTTTATTTTTCAAAATCATCATTTTTCAAACATCGTTTTTCATTTTATTAACGTTCTGTTGTGTTTGTTTTTATTTATTTATTTTATTTTACGTGAAAAAAAGCTCAAATTCTAGTGACACTCGTTTTATTTTCAAATTTTAAAGGGAAATGGCTCCGAAAACTGGAGAAACCGTCGTTGAAAAAATGGAGG

>chrIII:11425800-11426799_*twk-31*

TCGTTCCGCGCGGAGCACGCGGGAATGTGTGATAAGGGAGCAAAGATTGAGAGATTGAGAGGTCTATTTTTGAATTGAAACAAGAGAAAAAACTGGTACTGTCAAGATAATCGGAGAAGTGTCAAGTTTTCTAGTTTGCGCGCGCGCGCGAGTTGAAGAACAGCTGTGTGAGGAGAATCAAATGGTCAAGAGAAACTACAGTAGTATTTTGAAGAGGGTACATAGTTGTTACGGCAGAGTTTTAACTTTACTATAATTGCAAATTTTCTGAGAACAAATTACCATATGTCATGTGCTTTCTCGAATTTCCCAGAAAGTTCTAGAGCATTCTAAATTTTTTTTTGAATTTTTCAGAAGGTTCCTCAACATTCCAAAATGTTCTCGAAATTTCCAGAAGGTTCTAGAACATTTCAAATTTTTTTTTTGAATTTTCAGAAGGTTCTAGAACATTCCAAACATTTTTCGAATTTTCCAGATTTTTCCAGAAGGCTCTAGAACATTTCAGAAGTTTCTCGAATTTTTCAAAAGGTTCTAGAACATTTCAGAATATTCTCGAATTTTTCAGAAGGTTCTAGAACATTCCTGAATTTTCTCGAATTTACCAGAAAGCTCTAGAACATTCCAAAATTTTCTCGAATTTTCCAGAAGATTCTAGAACATTTCAGAATTTTCCCGAATTTTCCAATATCCGTTCAATAGACGAAAAGTCAATTTTTCCCAAACTACAGTAATCCTACAGTACTCCTACAGTACCTCTACAGAACAACTACAGTACCCCAACCATATCCCGACACTAACCCCAAAGCAATATCACTTCGGTTCTAGAACATTCCAAAATTTTTTTGAGTTTTCTAGAAGGTTCCTAAACATCCCAAATTTTTTTTGAATTTTCCAAAAGGTTCTAGAACATTTCAGAATTATCTCGAATGTTCCAGAAGGTTTTAAAACATTCCAAATTTTTCTCGAATTTTTCAGAATTTTCCAAAAAGTTCTAGAACAT

>chrIII:12001200-12001999_Y56A3A.33

GAGCATCTCGAGCATTTGCGGTAGAAATCTGGAAATTTTTTTTTTAATTCTTTTGGTCAGTTTCTAGTGAAATTGAGCTTTATTACGGGAACACCAAATTCTGAAAATGCGTACTGCGCAACATATTTGACGCGCAAAATATCTCGTAGCGAGAACTACTCAATAAATGACTATTGTAGTGTCGATTCACGGGCTCGAGAAGCTTTATTTGATTTCTCGTATTTTTCCAGAACATTTCAGGTTTTTCTCGAATTTTCCAGAAGGTTCTAGAACATTCCAGAATTTTTTCGAATTTTCCAGAAAGTTCTAGAACATTCCAGAATTTCTTGGAATTTTCCAAAAGGTTCTAGAACATTCCAGAATTTCTTGGAATTTTCCAAAAGGTTCTAGAACATTCCAGAATTTTTTTTAAATTTTCCAGAAGGTTCTAGAACATTCCAGAATTTTTTAAAATTTTCCAGAAGGTCCTAGAATATTCCAGAATTTTTTCAAATTTTCCAGAAGGTTCTAGAACATTCCAGAATTTTTTAAAATTTTCGAGAAGGTCCTAGAATATTCCAGAATTTTTTTCAAATTTTCCAGAAGGTTCTAGAACATTCCAGAATTTTTAAAAATTTTCCAGAAGCTTCTAGAACATTCCAGAATTTTTTAAAATTTTCCAGAAGGTCCTAGAATATTCCAGAATTTTTTTCAAATTTTCCAGAAGGTTCCAGAACAAATATGTTGCACGATACGCATTGTGTTCCCATAATAAGAATAGTTTTCAAAATTTCAAATTCCGGAAAATCAAGCAAGTTCGC

>chrIII:12123200-12123799_Y75B8A.6

AATGTTGAAATGAGAGAAAGAAAAAAAGAGAAGAGGAGAAGAAAAGGACTCAGTTTTCAATACTTCCTAACTAGATTCCTCCTCCTCTTCTTCTTTTTTTTTCTTTTTAAATTGCAACTTTTTCAATGGGGTGAAGAACAATAAATAAGTAAAAAAATAGGAAAATTAAAAGGTTTTGTAAGGCACTGTGTTGTACTGGAAGTTTTTAGAAAACTGGTTGCGGAAATGCTGGGAAATTTTTGGGGTTTTGGGAATTTTTTGTGAGCTGAAATTGCTACTCCAACGTTTATTTTTTGCAGAGTTTCAATAATAGAATATCCGACGACGTTCCAAAATTTTCTAAAAATTTTCCAGAAGGCTCTAGAACATTCCAGAATTTTTTCGAATTTTCCAGAAGGTTCTAGAACATTCCAGAATTTTTTCGAATTTTCCAGATAGTCCTAGAACATTCCAGAATTTTCTAAAATTTTCCAGAAGGCTCTAGAACATTCCAGAATTTTCCAAAAAATTTTTTTAGCTTTTTGAAGCCATTAAAATGATCAAAAAAATTGAGGAAGCTTTTAAAAAAAAAAGTATTTTTTTCTGAATTTTTTGAAAATT

>chrIII:12996400-12998199_ZK1010.8

ATCGTCTTGTACTTCCACGGAAAACCGATCTGGTCTGGTTCGGGCGCGTAGAAGTCGAGTCTGAAGAAAGTGTGAAAATTTTTGCGTGTGAGAGAGGGGTCTAGAGGGCTATTCACTAGAACATTTAAAAAATCCAGACGCTTCTAAAATGTTCTAGAACCTTCAGAAAAATTCCAAAAAAAATCCTGGAATTTTCGTGAACCTTCTGAAAAATTTGGCAAAATTTTGGAATGTTCTAGAACCTTCGGAAAAGTTTTAAAAAAATCTGGAATTTTCTAGTACCTTCTGAAAAATTCGGCAAAATTTTGGAATGCTTTAGTACCTTCTGAAAATTCCAAAAAAATCTGGAATGTTTTAGAACCTCCTGAAAAATTTGGCGAAATTTTGGAATGTTCTAGAACCTTCTGAAAAATTCTAGACACCTCTGAAATGCTCTAGCACCTTCTGAAAAATTTAAAAAAATATATTTTGGAATGTTCAAGAACTTTTTGAAAAATTACAAAAACATCTGGAATGTTCTAGAACCTTGTGAAAATGTTTTTTTTTTAATTCTGGAATGCTCTGGAACCTTCTGAATAATTCAAAAAAATTCTGGGATGTTCTAGAACTTTCTGAAAAATTTAACAACATTTTGGAATGTCCTAGAACCTTCTGAACATTTTTTTGGAAATTTTTGGAATGCTCTAAAACCTTCTGAAAAATTTTTAAAAATTCTGGAATGTTGTAGAACCTTCCCAAAATTCAAAAAATATTCTGGAATTTTCTAGAACCTCCTGAAATTTTTTTTAAATTTTGGAATGTTCTAGAACTTTCCGGGAAATTCGGAAAAATTGTGAAATGTTCTAGAATCTTTTGAAACATTCATAAAAATTCTGGAATGGCCTAGAACCTTCTGAAAAACTTGGCAAAATTTTGGAATGTTCTAGAGCCTTTTGAAAAATTCAAAAAAAATTTTTGGAATTTTCTAGAACCTTCTGAATCTCTCTAGACACCTCTTAAATGTTTTAGAACTTTCTGAAAAATTCACAAAAACGCTGAAATTTTTTAGAACCTTCTGAAAAATTTTATACACCTCTGAAATGTTCTAGAACGTTCTGAAAAATTCACAAAAACGCTGAAATTTTTTTAAACAAATAATAAAAATTTTCAGAAAATTCTGGAATGTTCTAGAACATTCCGAAAAATTTTAAATCGTTCTGGAATGTTCTAGAACTTTTTGGAAAATTTGGAAAAATTCTGGAATGTTCTAGAGCCTTCTGGAAATTTTAAGAAAATTCTGGAACTTTCTAGAACCTTCTGAAAAATTTTAGACACCTCTGAAATGTTCTAGAACCTTCTGAAACATTCCAAAAAAAATCTGGAATGTTCAAGAACCTTCAGAATAATTTGGCAAAAATTTTGGAATTTTCTATAACCTTCTGAAAAATTCACATAATTCTAGAATTTTTTTAGAACCTTCTGAACATTTTTTTAAAAAATTATAGAATTTTCTAGGACCTTTTGAAATTATTTCAAAAAACTTCTGGAATTTTCTAGAACCTTCTGAAAACTTCGGGAAAATTTTGGAATGTTCTAGAACCTTTTGGAAAATTTTAACAAATTCTGGAATGTTCTAGAACCGTCTGAAAAATATGGCAAAATTCTGGAATCATCTAGGGCCTTTTGAAAAATTCACAAAAATGCTGGAATGATCTAGAACCTTCTGAAAAATCGTAGACACCTCTGAAATGTTCTAGAACCTTCTAAAAAATTTTTTAAAAATTTCTGGAATTTTCTAGAACCTTCTGAAATTTTGGCAAA

>chrIII:13040400-13041399_F54F12.2

GCTCTATCCTCTGCGGCTAAAATGATGGTTCCAGGTATGTGAAATTTGAGAGTTTACTGAACAGGGTTTTAGGTTTTTTTCCAAATTTGGCGGAAATTAAATTTTTTTTTGATAAAAGTTTATATAACTTCGAAATATTTTTTATGTCTGAGAACGTTCCTGGATTCTTTGTGGGGATTCCAGAATTTTTTAAAATTTTTCAGAAGATTCTAGGGGATTCAGGATTTTTTCGAAATTTTCCAAAATGATCTATAACATTCCAGAATTTTTTTTTTAATTTTTCAGAATGTTCTAGAACATTCCAGAATTTTTTAAAATTTTCCAAAAGGTTTTAGAACATTCCAGAATTTTCTCGAATTTTCCAAAAAGGTTCTAGAACATTCCAGAATTTTCTCGAATTTTCCAAAAAGGTTCTAGAGCATTCTAGAACTTTCTGAAGTTTTCCAGAAGGTCCTAGAACAATTCAGAATTTTTTGAAATATTTCGAGAAGGTTCTAGAACATTTCAGAATTTTCTGAAAAATTCCAGTAGGTCCTAGAACAATTCAGAATTTTTGAAATATTTCCAGAAGGTTCTAGAACATTCCAAAATTTTCTGAAAATTTCCAGAAGGTTCTAGAGCATTTCAGAATTTTTTGAAAAATTTCAGAAGGTTCTAGAACATTCCAGATTTTTTTTAAAAATTTCCAAAAGGTTTTAGAACATTCCAGAATTTTCTCGAATTTTCCAAAAAGGTTCTAGAACATTCCAGAATTTTCTGAAATTTTCCAGAAGGTCCTAGAACAATTCAGAATTTTTTGAAATATTTCCAAAAGGTTCTAGAACATTTCAGAATTTTTTGAAAAATTTCAGAAGGTTCTAGAACATTCCAGATTTTTTTGAAAACTTTGAAAAGGTTCTAGAACATTCCAGAATTTTCTGGAAATTTTCAGAACATTCTAGAACATTTCAGAATTGTCTTAAAATTTTTGTAAGGTTCTAGAACATTCCAGAGTTTTCTG

>chrIII:13446600-13447999_*cua-1*

CTGACCGCGCCTACGGCGCGGGCAACGACTGGCACCATTAAAAGTATTTGACACACATACCTTCAATCACTCAAAAAATTTCTCATCATGAAAAAAACTTGACACACTTAAACGCGGTTTACTGGCACTGTTACACGTATTTTTCTACGACATGCATGTTTCGAAAATTCACAGACAGTATGAAAACGTTCTGTTACTTTCTGACCAAGACAGTAACCTTACAATACCACTACAGTACCTTGACATTATCCTCCACCGACTCCTAACTCAATACCTCTTCAAAGGACGAAATGCCAATTTTTCCAAAACTACAGTAACCCTACCGTATACCTACAGTACCCCTATAGTACCACTACAGTACCTTGACTTAATCCCCCATCAACTCCCAAATAACTACCTCTTCTAAAGCTCTAAGCTCAATTTTTCGGAACATTCCAGAATTTTCTCGATTTTTCTAGAAAGTTCTAGAACATTCCAGAATTTTCCCGATTTTTCTAGAAAGTTCTGGAACATTCCAGAATTTTCCCAATTTTTCTAGAAAGTTGTGAAACATTCCAGAATTTTCTCGATTTTTCTAGAAAGTTCTGGAACATTCCAGAATTTTCCCAATTTTTCTAGAAAGTTGTGAAACATTCCAGAATTTTCTCGATTTTTCTAGAAAGTTCTGGAACATTCCAGAAATTTCCCGATTTTTCTAGAAAGTTCTGGAACATTCCAGAATTTTCCCAATTTTTCTAGAAAGTTGTGAAACATTCCAGAATTTTCTCGATTTTTCTAGAAAGTTCTGGAACATTCCAGACTTTTTCCGATTTTTCTAGAAAGTTCTGGAACATTCCAGACTTTTCCCAATTTTTCTAGAAAGTTCTGGAACATTCCAGAAATTTCCCAATTTTTCTAGAAAGTTCTGGAACATTCCAGACTTTTTCCGATTTTTCTAGAAAGTTCTGGAACATTCCAGAAATTTCGCGATTTTTCTAGAAAGTTCAGGAACATTCCAGAATTTTCTCGATTTTTCTAGAAAGTTCTGGAACATTCCAGACATTTCCCAATTTTTCTAGAAAGTTCTGGAACATTCCAGAAATTTCCCAATTTTTCTAGAAAGTTCTGGAACATTCCAGACTTTTCCCAATTTTTCTAGAAAGTTCTGGAACATTCCAGACTTTTCCCAATTTTTCTAGAAAGTTCTGGAACATTCTAGAAATTTCCCAATTTTTCTAGAAAGTTCTGGAACATTCCAGACTTTTCCCAATTTTTCTAGAAAGTTCTAGAACATTCCAGACTTTTCCCAATTTTTCTAGAAAGTTCTGGAACATTCGAGAATTTTTTCGAAATTTCCAGAAGATTCTAGATTTCCAGAATTTTAGAATTTTCAGAAAATTTAAATTTCCCGCAAAAATATT
