# Supplementary material for: Whole genome profiling of short-term hypoxia induced genes and identification of HIF-1 binding sites provide insights into HIF-1 function in Caenorhabditis elegans
Source: PLoS One. 2024 May 14;19(5):e0295094. doi: 10.1371/journal.pone.0295094 (PMC11093353; doi:10.1371/journal.pone.0295094)
Supplement: S4 File — (DOCX) [file pone.0295094.s025.docx]

**S4 File. Sequences co-immunoprecipitated with HIF-1** **on chromosome 4.** The HRE similar sites were color coded as red on the reference Watson strand, and as green on the Crick strand.

>chrIV:1153600-1154199_C50A2.3

TTTGGGGGTTCTTAATAGAAAAAACGGATAAAGGAATATTCAAACTACTAAAAAACTTAAAAAAAAATATTTTTGAACCAGCCGCTGACCGCGCCTACGGCGCGGGCAACGACTGGCACCATTAAAAGTATTTGACACACATACACTTTCAGAATTTTCTCGATTTTTCTAGAAAGTTCTGGAACATTCCAGACTTTTCCCAATTTTTCTAGAAAGTTCTGGAACATTTCAGACTTTTCCCAATTTTTCTAGAAAGTTCTGGAACAATCCAGACTTTTCCCAATTTCTCTAGAAAGTTCTGGAACATTCCAGAATTTTCTCGATTTTTCTAGAAAGTTCTGAACATTCCAGACTTTTCCCAATTTTTCTAGAGAGTTCTGGAACATTCCAGACTTTTCCCAATTTTTCTAGAAAGTTCTGGAACATCCCAGACTTTTCCCAATTTTTCTAGAAAGTTCTGGAACATTCCAGACTTTTTTCCAGATTTTCCAAAATATTTTTCACAGAAAATTTAAATTTCCCTCCAAAATATTTTTTTCGGAAAATTTAAATTTCCCTCCAAAATATTTTTTTCAGAAAATTTAAATTTCCCGCCAAAAT

>chrIV:2096400-2096999_C23H5.11

TCATGGCATTTATTTTTGATCTATTAAAGAGATTCTAGAAATTTCAGAAAATTTTAGAACGCTCCACGAACTTCTAGACTTTTCCAGAAGATCATTCCAGAGTTTTGTCGAATTTTCTAGAAGGTTCTGGAACATTTCAGACAATTCTTGAATTTTCTAGAACGATTTAGAACATTCCAGAATTTTCCGATTTTTCTAGAAAGTTCTGGAACATTCCAGAATTTTCCGATTTTTCTAGAAAGTTCTGGAACACTCCAGAATTTTCCCGATTTTTCTAGAACGTTCTGGAGCATTCCAGAATGTTTTCGAAATTTCCAGAAGATTCTAGATTTCCAGAATTTTAGAACTTTCAGAAAGCTTAAATTTCCCGCCAAAATATTTTTTTCAGAAAATTTATATTTCCCGCCAAAATGTTTTCACTAAAAATTTGAATTTCCCGCCAAAATATTTTTCACTAAAAATTTGAATTTCCCGCCAAAATTTTGGGTCTTACCACGGTGGGTCTCACCACGATGGGTCTCGCCACGATGGGTCTCACCACGACGGGTCTCACCACTGTGGGTCTCGCCACGATGGGTCTCACCACGAAGGATGACAGAC

>chrIV:2113600-2113999_C23H5.8

GAAAATTTTATTTTTCGGAAACATTTTTCAATTTAGCTCTTTTTTTCAAAAAATAAAAAATGTCAATTTTTTATTTAGGATTTATTTATTTTTTCAGTTTTTGAAATATTTTTCTATGAGATACCAGCCGATGACCGCGCCTCCGGCACCACAACAATTCCAGAATTTTCACGAATTTTCTCGAATTTTCCAGAAGTTTCTAGAACATTCCAGAACTTTCTCGAATTTTTCAGAAGCTTCTAGAAAATTCCAGAATTTTTTTTCGATTTTTCCAGAAGGTTCTAGAACATTCCAGAATTTTCTCGAATTTTTCAGAAGGTTCTAGAGCTTTCCAGAATTTTCCAGAAGGTTCTGGAACATTCCAAAAGTTTCCCGAAGTTTCCAATTTCCCTTCCAAAGA

>chrIV:2683600-2683799_*egrh-3*/Y94H6A.11

TTGGAGGGAAATTTAAACTTTCTGAAAATTCTAAAATTCTGGAATGTTCCAGAACTTTCTAGAAAAATCGGGAAAAGGCTGCAATGTTCTAAATCGTTCTAGAAAATTCAAGAATTGTCTGAAATGTTCCAGAACCTTCTAGAAAATTCGACAAAACTCTGGAATGATCTTCTGGAAAAGTCTAGAAGTTCGTGGAGCGT

>chrIV:2694800-2695199_Y94H6A.5

AATTTCCCGGTAAATGTTTTTCAGAAAATTTGAACTTCCCGTCAAAATGTTTTTTTTTCGGAAAATTTAAATTTCCCGCCGAAACTTATGGGTCTCACCACGATTGGTCTCACCACGAACATTCCAGATTTCTTATCGAATTTTCCAGAAAGTTCTAGAAAATTCCAGAATTTTTTCGAATTTTTCAGAAAGTTCTAGAACATTCCAGAATTTTTTCGAATTTTCCAGAAATTTCTAGAAAATTCCAGAACGTTTTCGAATTTTCCAGAAGGTTCTGGAATATGCCAGATTTTTCTCGAATTTTCCAGAACGTTCTAGAACATTCCAGAATTTTCCAGAAAATTTCAGAAGGTTCTAGAACATTTCAGAATTTTCCCGAAGTTTCCAATATTCCTACCAA

>chrIV:4725400-4725799_K08F11.1

TTTTCCCATTTTTTAAATTTATATTTGACGCCACTGCAGGAATGAATGAGATTTCCTTGTCCCACATACCACAATACATTATCTTTTAAATTCTGAACTGATTGTGCATGCAAAAGTGCATCGATGTGTTTCACGGTAGCACAGAAACTATCACGGCCCGGCGGGGGGTACATGGACGAGAATTCTCTACCGTATTCCAATTTGGCTGACTGCGTGCTCAACGTTGAATACTCAGTGTAAACTTTCGTACACCGTTGCGTACTGCACAGCGCGCATTTTAATTGACGACATTTAGCAAAAATTGAACAGAAGATTTTTCGGAATTATGAAGCTCAATTTTCACAAAAATAATGAGTTTTTTGTAGAATTTATGAAAAAACGTGAATATATAGATTTTTTG

>chrIV:4727800-4728199_K08F11.1

GGCTCACCCGGTCGATTTTTGCGGCGATTTGTGTTCTTTCGCTGAAAATATTATTTTTATTTCAATTATTAACGAAGAAAACAAGAAAAAACGACGAGAAAACATCAAAAAAACGCGAAAAAACATCGAAAAACCACCGAAACCTCATGAAAAAAATAAAGCATTGCAGCCGCGGGATTAGTTTTCGCAACTTTCTAGGCCATGTCCCGTTCGCCGTGCCGTGAACTAGATCTCTCGTAAAATTTGAGAAAGATCTCGCAGGTACGCAGCGAAATGGTCCGCAATGTGTCATCGCGGTGTTTGCGTACTTGCGTACCGTAGTCCGCAAAACATTGCAGCGGCAAATAGATTTTTGAAGCAAATTTTAGCAGAAAAAAGGCAGAATTAATAGTTTCAAGGT

>chrIV:4749800-4750199_*rpl-20*

CAAGGAATGGGGAATGAACAATCTGATATAATAGCGAAAAACAATTTTAGGATAACGTTACAATTTTCTTTTAAAATGGAAAGGTAGATGGGAATTACCGAAAAAATATGAGTGCATATTGCAATATTTAAAAACTATGAAAAAACTGTTAAATTTTTGTAAAAATCAGAAAAAGAAAAGCGGAAAAAAGCTAAAAAATAGGATCAGGAAAACCCAAAATGAATTTCAACGCACAAAAGTGTATGCGGCAGTGCTAGTTTTGTTGCGTGTTCTATTACCGTATCCTTCATACACTCGCCGTCGATTTACGGGGAGCGGTGGAGCGCACTTACTCCTTAGATTTTTAAGCAATTTTTTTGTTAAAAAGATATAGAAATGTGATTTTAAATGTTTTATTTTA

>chrIV:4897800-4898199_T08B6.9

CTGTAGTTTAGGAAAAATTGAGTTTTTGTCTTTTGAAGAGGAAAATTCTTTTTTTGTAAAATTCTAGAATGTTCCAGAACCTACTGGAAAATTCTGGAATGTTCCAGAACTTTCTAGAAAAATCGAGAAAATTCTGGAATGTTCCAGAACCTTCTGGAAAATTCGAAAAAATTCTGGATTGTTCTAGAACCTGCTGGAAAATTTTAAAAAATGTTGTTCCGAGATCTTCGTGGAGAAACCCATCGTGGCGAGACCCATCGTGGTGAGACCCGTCGTGGTGAGATCCATCGTGGCGAGACCCACCGTGGTGTGACACATCGTGGTGAGACCCATCGAGGTGAGACCCATCGTGGTGAGACCCATCGTGGTGAGACCCATCGTGGTGAGACCCATCGTGGTA

>chrIV:8468600-8469399_*oat-1*

CACACATACACTTCCAGAATTTTCCCGATTTTTCTAGAAAGTTCTGGAACGTTCCAGAATTTTCTCGATTTTTCTAGAAAGTTCTGGAACGTTCCAGGGAACATTCCAGAATTTTCCCAATTTTTCTAGAAAGTTCTGGAACATTCCAGAATTTTCCCAATTTTTCTAGAAAGTTCTGGAACATTCCAGAATTTTCCCAATTTTTCTAGAAAGTTCTGGAACATTCCAGAATTTTCCCAATTTTTCTAGAAAGTTCTGGAACATTCGAGAATTTTTTCGATTTTTCTAGAAAGTTCCGGAACACTCCAGAACTTTCTCGATTTTTCTAGAAAGTTCTGGAACATTCCAGAATTTTCCCGATTTTTCTAGAAAGTTCTGGAACGTTCCAGAATTTTCTCGATTTTTTTAGAAAGTTCTGGAACACTCCAGAATTTTCCCGATTTTTTTAGAAAGTTCTGGAACACTCCAGAATTTTCCCGATTTTTCTAGAAAGTCTGGAACGTTCCAGAATTTTCTCGATTTTTTTAGAAAGTTCTGGAACACTCCAGAATTTTCTCGATTTTCTAGAAAGTTCTGGAACATTCCAGAATTTTCTCGATTTTTCTAGAAAGTTCTGGAACATTCCAGAATTTTCCCAATTTTTCTAGAAAGTTCTGGAACATTCCAGAATTTTCCCGATTTTTCTAGAAAGTTCTGGAACATTCGAGAATTTTTTCGAAATTTCCAGAAGATTCTAGATTTCCAGAATTTTAGAATTTTCAGAAAATTTAAATTTCCCGCCAAAATATTTTTCTCAGAAA

>chrIV:9196200-9196599_C46C2.5

TATCAGCAACATACAATCCTTTAAAATGATTATTTTTTGTAAATTCGATAAAAATTAATTTATTTTTCACAATTTCTGCCCGAAAATTGCCGAAATAACCAGCGTTTCTATAACTAAAACAAGTGTCGTCAATTAAAATGCCGCATCCGCAAAATGTCGTACGAAACTTTTCGCTGAGTATCAAACGTTGAATATTCAGTCAGCCAAATTTTACTACGGTAGAGATTTTACAGCCACGTACGGTTCGCCGGGCCGTGGCACGTGGCAGAAATGTCTGCAATCTGACGTGACAGTGTTTGCACGCATTGCATTATACCCGACGACACTTTTGTGTTCCATGTTTTCATTCAAATGCCAAAATTTTCATTTTTAGCTTTTCAAGTTTTATTTAACTATTTTC

>chrIV:11480400-11480799_*mbf-1*

ACATTTAAAACTTGACAGACGGAAGGAAGAACTCTTTTCAATCGACGATTGAACACGTAATTGCTCTCTGTATGAACAATTGTCATAAAAATTATTCAAATTTCAAGAAAAAAGAAAACACTCAGAAAATACGTAAAATTTCAGTCCAAAAACAAAAAGAGAATCGTTTATTTTTCGAGAAAGTTCCACGGAGCCATGAGGTTTGTGTGCAAACACTGTGACGCGCAATTGCGGAGCGAATTTCAAAATCATGTTTTTTTGTTTGCAATTTGTTTGTATTTTGCAACATTTTCACTCTCTTGAACCCTTCTCCTTGATATTTTTCTATTCAGAACGTTTTTAAAGCTTTATCAAATTACATTCTCAGGAGTAATCGTAAATAATCTACTCATGAAAACCA

>chrIV:12155800-12156199_*eri-12*

TATTTTTAAGGGTCCCACCACGATGGGTCTCGCCAGTTCGAGGTGGTACTTAAGCTAACAAAAAGTTTCTCAGAAAATTTGAATTTCCCGCCAAAAATTTTTTCTCAGAAATTTTGAGTTTTCGGTCAAAATTTATGGGTCTCACCACGAATGTTCCAGAATTTTTTTCGAATTTTCCAGAAGGTTCTGGAACTTTCCAGAAGGTTCTGGAACTTTCCAGAAAGTTCTGGAACATTCCAGAATTTTCTCGAATTTTCCAGAAGTTCTAGAACATTATAGAATTTTCTGGAATTTTCCAGATGATTCCAAAACCAAAATTGAAATTCCCGCCAAAATCTTTTTCTCAGAAAATTTGAATTTCCCTCCAAAAATTTTTTTACTGAAAATTTGAATTTCCTGC

>chrIV:13847200-13847799_*rga-5*

AAATATTTTGGAGGGAAATTTAAATTTTCTGAAAAAAAACATTTTGGAGGGAAATTTAAATTTTCTGAAAAAAATATTTTGGAGGGAAATTTTTAATTTTCTGAGAAAAATATTTTGGAGGGAAATTTGAATTTTCTGAAAATTCTAAAATTCTGGAAATCTAGAATCGGGAAAATTCTAGAATGTTCCAGAACTTTCTAGAAAAATCGAGAAAATTCTGGAATGTTCCAGAACTTTCTAGAAAAATCGAGAAAATTCTGGAATGTTCCAGAACTTTCTAGAAAAATCGAGAAAATTCTGGAATGTTCCAGAACTTTCTAGAAAAATCGAGAAAATTCTGAAATGTTCCAGAACTTTCTAGAAAAATCGAGAAAATTCTTGAATGTTCCAGAACTTTCTAGAAAAATTGAGAAAATTCTGGAAGTGTATGTGTGTCAAATACTTTTAATGGTGCCAGTTGTTGCCCGCGCCGTAGGCGCGGTCAGCGGCTGGTATATATATATTAAAAAACCTAAAACAAACATGTGAGATACACCGGCTTAAATTGAAAATTGCAGAGCAGAATTAAAAAAATATATATTTAGAAGAACCACCACGATT

>chrIV:14218000-14218599_*sqrd-1*

TGTTTCACTGCGATGGGTCTCACCACGATGGGTCTCACCACGAAGGGTCTCACCACGATGGGTCTCACCACGAAGGGTATCACCACGAAGGGTCTCACCACGATGGGTCTCACCACGAAGGGTCTCACCACGATGGGTCTCACCACGAAGGGTCTCACCACGAAGGGTCTCGCAGCGGCTTTTTTTTTAATTTTCCAGAAGGTTCTAGAACATTCTACAATTTTCTCGAATTTTCTAAAAGGTTCTAGAACATTCCAGAATTTTCTCAAATTTTCCAGAAGGTTCTGGAACAGTCCAGAATTTTCTCGAATTTTCCAGAAGGTTCTGGAACAGTCCAGAATTTTCTCGTGTTTTCCAAAAGGTTCTGGAACATTCCAGAATTTTCTCGAATTTTCCAGAAGGTTCTGGTACATTTCAGAATAATGTTTTCAAAAAATTAAAATTTGAATTCCCGCCAAAATGTTTTAAAAAAATTCCAATTTGAAGTCCCGCCAAAATGTTTTCAAATATTAAAATTTGAATTCCCGCCAAAATGTTTTCAAAATTTAAAATTTGAATTCCCGCCAAAATGTTTTCAAAATTTAAAATTTGAATTCCCAC

>chrIV:14480200-14480999_Y57G11A.4

AAGACCCATCGTGGTGAGACCCATCGTGGTGAGACCCGTCGTGGTGAGACCCACCGTGGTAAGACCCAAAATTTTGGCGGGAAATTTAAATTTTCCGAGAAAAATATTTTGGCGGGAAATTTAAATTTTCTGTGAAAAATATTTTGGCGGGAAATTTAAATTTTCTGAGAAAAATATTTTGGAGGGAAATTTAAATTTTCTGTGAAAAATATTTTGGCGGGAAATTTAAATTTTCTGTGAAAAATATTTTGGCGGGAAATTTAAATTTTCTGAAATTCTAAAATTCTGGAAATCTAGAATCTTCTGGAAATTTCGAAAAAATTCTCGAATGTTCCAGAACTTTCTAGAAAAATCGAGAAAATTTTGGAATGTTCCAGAACTTTCTAGAAAAATTGTGAAAAGGGTGGAATGTTCCAGAACTTTCTAGAAAAATCGAGAAAATTCTGGAATGTTCCAGAACTTTCTAGAAAAATTGGGAAAAGTCTGGAATGTTCAAGAACTTTCTAGAAAAATCGAGAAAATTCTGGAATGTTCCAGAACTTTCTAAAAAAAATCGAGAAAATTCTGGAATGTTCCAGAACTTTCTAGAAAAATCGGGAAATTTCTGGAATGTTCAAGAACTTTCTAGAAAAATCGAGAAAATTCTGGAATGTTCCAGAACTTTCTAGAAAAATTGGGAAAATTCTGAAATGTTCCAGAACTTTCTAGAAAAATCGAGAAAATTCTGGAATGTTCCAGAACTTTCTAGAAAAATCGAGAAAATTCTGGAATGTTCCGAAAAATTGAGCTTAGAGCTTTAG

>chrIV:15058800-15059999_Y41E3.13

AAAACGCGGTTTACTGGCACTGTTACATGTATGTATTTTTCTACGACATGCATGTTTCAAAAATTCACAGACAGTATGAAAACTTTCTGTTACTTTTTGACCAAGACAGTAACCTTACAATACCACTACAGTACCTTGACATTATCCTCCACCGACTCCTAACCCAATACCTCTTCAAAGGACGAAAAGTCAAATTTTCCAAAACTACAGTAACCCTACCGTATACCTACAGTACCCCTATAGTACCACTACAGTACCTTGACTTGATCCCCCATCAACTCCCAAATAACTACCTCTTCTAAAGCTCTAAGCTCAATTTTTCTGAACATTCCAGAATTTTCTCGATTTTTCTAGAAAGTTCTGGAACATTCCAGAAATTTCCCGACTTTTCTAGAAAGTTCTGGAACATTCCAGAATTTTCTCGATTTTTCTAGAAAGTTCTGGAACATTCCAGAATTTTCTCGATTTTTCTAGAAAGTTCTGGAACATTCCAGAAATTTCCCGATTTTTCTAGAAAGTTCTGGAACATTACAGAAATTTCCAGATTTTTCTAGAAAGTTTTGGAACATTCCAGAATTTTCTCGATTTTTCTAGAAAGTTCTGGAACATTCCAAAATTTTCCCGATTTTTCAAGAAAGTTCTGGAACATTCCAGAAATTTCCCGATTTTTCTAGAAAGTTCTGGAACATTACAGAAATTTCCCGATTTTTCTAGAAAGTTCTGGAACATTCCAGAATTTTCCCAATTTTTCTAGAAAGTTCTGGACCATTCCAGACTTTTCCCAATTTTTCTAGAAAGTTCTGGAACATTCCAGAAATTTCCCAATTTTTCTAGAAAGTTCTGGAACATTCTAGACTTTTCCCAATTTTTCTAGAAAGTTCCGGAACATTACAGAAATTTCCCGATTTTTCTAGAAAGTTCTGGAACATTCCAGAAATGTCCCGATTTTTCTAGAAAGTTCTGGAACATTCCAGAATTTTCCCAATTTTTCTAGAAAGTTCTGGAACATTCCAGAATTTTAGAATTTTCAGAAAATTTCAATTTCCCGCAAAAATATTTTTCACAGAAAATTTAAATTTCCCTCCAAAATATTTTTCACAGAAAATTTAAATTTCCCTCCAAAATATTTTTCACAGAAAATTTAAATTTCCCGCCAAAATATTATTCACAGAAAATTTAAATTTCCCGCCAAAATATTTT

>chrIV:15166600-15167199_Y40H7A.4

CTATTTTGGAGGGAAATTTAAATTTTCTGTGAAAAATATTTTGGCGGGAAATTTAAATTTTCTGTGAAAAATATTTTTGCGGGAAATTGAAATTTTCTGAAAATTCTAAAATTCTGGAAATCTAGAATCTTCTGGAAATTTCGAAAAAATTCTCGAATGTTCCAGAACTTTCTAGAACAATCGAGAAAATTCTGGAATGTTCCAGAACTTTCTAGAAAAATCGAGAAAATTCTGGAATGTTCCAGAATTTTCTAGAAAAATCGAGAAAATTCTGGAATGTTCCAGAACTTTCTAGAAAATTCTGGAAAATTCTGGAATGTTCCAGAACTTTCTAAAAAAATCGAGAAAATTCTGGAATGTTCCAGAACTTTCTAGAAAAATTGGGAAATTTCTGGAATGTTCCAGAACTTTCTAAAAAAATCGAGAAAATTCTGGAATGTTCCAGCACTTTCTAGAAAAATTGGGAAATTTCTGGAATGTTCCAGAACTTTCTAGAAAAATCGAGAAAATTCTGGAATGTTCCGAAAAATTGAGCTTAGAGCTTTAGAAGAGGTAGTTATTTGGGAGTTGATGGGGGATCAAGTCAAAGTACTGTAGTGG

>chrIV:15373800-15374599_Y73F8A.20

ACAGTAACCTTACAATACCACTACAGTACCTTGACATTACCCTCCACCGACTCCTAACCCAATACCTCTTCAAAGGACAAAATGCCAATTTTTCCAAAACTACAGTAACCCTACCGTATACCTACAGTACCCCAATAGTACCACTACAGTACCTTGACTTGATCCCCCATCAACTCCCAAATAACCTCTTCTAAAGCTCTAAGCTCAATTTTTCGGAACATTCCAGAATTTTCTCGATTTTTCTAGAAAGTTCTGGAACATTCCAGAATTTTCTCAATTTTTCTAGAAAGTTCTTGAACATTCCAGACTTTTCCCAATTTTTCTAGAAAGTTCTGGAACATTCCAGAATTTTCTCAATTTTTCTAGAAAGTTCTTGAACATTCCAGACTTTTCCCAATTTTTCTAGAAAGTTCTGGAACATTCCAGAAATTTCCCGATTTTTCTAGAAAGTTCTGGAACATTCCAGAATTTTCTCAATTTTTCTAGAAAGTTCTTGAACATTCCAGACTTTTCCCAATTTTTCTAGAAAGTTCTGGAACATTCCAGAATTTTTTCGATTTTTCTAGAAAGTTCTGGAACATTCCAGAATTTTCTCGATTTTTCTAGAAAGTTCTTGAACATTCCAGAAATTTCCCGATTTTTCTAGAAAGTTCTGGAACATTCCAGACTTTTCCCAATTTTTCTAGAAAGTTCTGGAACATTCCAGACTTTTCCCAATTTTTCTAGAAAGTTCTGGAACATTCCAGACTTTTCCCAATTTTTCTAGAAAGTTCTGGAACATTCCAGAATTTTCTCGATTT

>chrIV:15530600-15530999_*ari-1.4*/*tag-349*

AATCCATTTATTTTTCACAATTTCTGCCCGAAAATTGCCGAAATAACCAGCGTTTCTATAACTAAGAAAGCGTCGTCAATTAAAATGCCGCGTCCGCAAAATGTCGTACGAAACTTTTCGCTGAGTATCAAACGTTGAATATTCAGTCAGCCAAATTTTACTACGGTAGAGAATTTACAGCCACGTACGGTTCGCCGGGCCGTGCGCCGGAATTTTCAAATCAATTTTTTTGTTGATTTTCTGGCGGAAAAAGAGGATTTTGAATTGATTTATGTAAAAGGAAAAGGATCGAAACGCGTTAAATAATTCAAAATATTAAAAAGTATAGAAACCGAAAATGAAATTTTAATTTTTAGCTGAACATTTGTTTGTACAGTTTCACCAATTTGATAAATTTGTC

>chrIV:15895600-15896399_Y105C5B.5

TCAAAAATTCACAGACAGTATGAAAACTTTCTGTTACTTTTTGACCAAGACAGTAACCTTACAATACCACTTCAGTACCTTGACATTATCCTCCACCGACTCCTAACCCAATACCTCTTCAAAGGACCAAAAGTCAAATTTTCCAAAACTACAGTAACCCTACCGTATACCTACAGTACCCCTATAGTACCACTACAGTACCTTGACTTGATCACCCATCAACTCCCAAATAACTACCTCTTCTATGGCTCTAAGCTCAATTTTTCGGAACATTCCAGAATTTTCCCGATTTTTCTAGAAAGTTCTGAACATTCCAGACTTTTCCCAATTTTTCTAGAAAGTTCTGGAACATTCCAGAATTTTCCCGATTTTTCTAGAAAGTTCTGAACATTCCAGACTTTTCCCAATTTTTCTAGAAAGTTCTGGAACATTCCAGAATTTTCTCGATTTTTCTAGAAAGTTCTGGAACATTCCAGAATTTTCTCGATTTTTCTAAAAAGTTCTGGAACATTCCAGAATTTTCTCGATTTTACTAGAAAGTTCTGGAACATTCCAGAATTTTCTCAATTTTTCTAGAAAGTTCTGGAACATTCCAGAATTTTCTCGATTTTTCTAGAAAGTTCTAAAGCTTTTCAGAATTTTCCAGAAGGTTCTGGAACATTTCAGAATTTTCCCGAAGTTTCCAATTTCTCTTCAAAAGACAAAAACTCAATTTTTCCTGAACTACAGTAATCCTACAGTACTCCTTCAGTACCTCTACAGTACTACTACGGTACCCCGACCATATCCCACTACTAACC

>chrIV:16615000-16615599_21ur-2039

AGTCTGTGACGTCATAGCTTACAAAGGCAAGGCGTTTTCGGCTGTTGGAGGGATATTAGTTTGGGGTTAGTAGTGGGATATGGTCGGGGTACTGTAGTAGTACTGTAGAGGTACTGTAGGAGTACTATAGGATTACTGTAGTTTGGAAAAAATTGACTTTCCGTCTTTGGAAGGGAAATTGGAAACTTCGGGAAAATTTTGGAATGTTCCAGAACCTTCTGGAAAATTCTGGAATGTTCTAGAACCTTCTGGAAAATTCGAAAAAAAATTCTGGAATTTTCTAGAACCTTCTGGGAAATTAGAAAAAAAATTCTGGAATGTTCTAGAACCTTCTGGAATATTCGAGAAAATTCTGGAATTTTCTAGAACCTTCTGGAATATTCGAGAAAATTCTGGAATGTTCTAGAACCTTCTGGAAAATTCGAGAAAATTCGTGAAAATTCTGGAATTGTTGTGGTGAGACCTATCGTGCTGAGACCCATTGTGGTGAGACCCTTAAAAGTTTGGGCGGGAAATTCAAAATTTATGAGAACATTTTTTTGAGGGAAATTCAAGTTTTCTGAGAAACTATTTTTGTTTCAGTACCTTCTGGAAGTTTCA

>chrIV:16675400-16675999_*sta-1*

ATTTGATTTCTGCAGAAAATTAGTCTGTTTTTTCCAAAACTTTAAACCGCCATAACTTTTTTTTGAGAAATTTTCAAAACGTCTCAATATGAAATTTGGCAGTTTTCGGGTAATTTTGAGTCTGAAGTTAATTTTCTTTTGATCTCTTTTTTAAAGAGATTCTAGAAATTTCAGAAAATTTTAGAACGCTCTAAAAGTTTCTAAGACATTCCAGAATTTTCCCGATTTTTCTAGAAAGTTCTGGAACGTTCCAGAATTTTCCCGATTTTTCTAGAAAGTTCTGGAACGTTCCAGAATTTTCCCGACTTTTCTAGAAAGTTCTGGAACGTTCCAGAATTTTCCCGATTTTTCTAGAAAGTTCTGGAGCATTCCAGAATTTTTTCGAAATTTCCAGAAGATTCTAGATTTCCAAAATTTTAGAATTTTCAGAAAGTTTGAATTTCCCGCCAAAATATTTTTCTCAGAAAATTTGAATTTCCCGCCTAAATGTTTTCACTAAAAATTTGAATTTCCCGCCAAAATATTTTTCACTGAAAATTTGAATTTCCCGCCAAAATATTTCTCACAAAAAATTTGAATTTCCTGCCACGATTTTTTCAC

>chrIV:16845200-16845799_Y116A8A.150

TACTATACTTAAAAAATCGATTAAAAAAAGGCGAAATTTTGAGCATTTGTCAACGTGCAATTCAAAAAGCAGAAAAAAATCAAAAAAAATCAGAAAATGCTGTAAAAAAAGAAACAGCCGCACTCCAAGTCACCCATTAAAAAAAATGACTAAAAATGGGAAACAATTCCTCACTTTGCGCAAAAAAATGCCGCGTTGAATGAGAGAAAGAGGGACGAAAAAAACTGAGTAGATCAAAAATAAATTTTTTGTGGCAGGTCTGGAAAAAAAGTTTTGCGCGTCAATTAAAATGCCTTGTACGCAAATGCGCGTCGCCTCGACGCGCCTCATCTGCAAACATGCTCGTGCTCCCGGGATCCCACGGCCCGGCGAACCGTACGTGGCTGTAAAATCTCTACCGTAGTAAAATTTGGCTGACTGAATATTCAACGTTTGATACTCAGCGAAAAGTTTCGTACGACATTTTGCGGACGCGGCATTTTAATTGACGACACTTGTTTTAGTTATAGAAACGCTGGTTATTTCGGCAATTTTCGGGCAGAAATTGTGACAAATAAATGAATTTTTATCGAATTTACAAAAAATAATCATTTTAAAGGA

>chrIV:17076000-17076199_Y116A8C.25

AACAAAAATAGTTTCTCAGAAACTTGAATTTCTCTCAAAAAAAATTTTCTCATAAATTTTGAACTTCCCGCCCAAATTTTTAAGGGTCTCACCAAGATGGGTCTCACCACGATAGGTCTCACCACGACCATTCCAGAATTTTCTCGAATTTTCCAGAAGGTTCTAGAAAATTCCAGAACTTTCTCGAATTTTCCAGAAGG
