# Supplementary material for: Whole genome profiling of short-term hypoxia induced genes and identification of HIF-1 binding sites provide insights into HIF-1 function in Caenorhabditis elegans
Source: PLoS One. 2024 May 14;19(5):e0295094. doi: 10.1371/journal.pone.0295094 (PMC11093353; doi:10.1371/journal.pone.0295094)
Supplement: S5 File — (DOCX) [file pone.0295094.s026.docx]

**S5 File. Sequences co-immunoprecipitated with HIF-1** **on chromosome 5.** The HRE similar sites were color coded as red on the reference Watson strand, and as green on the Crick strand.

>chrV:126600-126999_W03F9.1

GTGCCGTAAATTCTAGCAAAAACACTTACATCTTGAGAATCCGCAGCCATTTTACTTTAAAAAATGAATTTGTTGATTAAAATCGAAAATTATCTGCTTTAAAGTATTAATTTTAGAGAGAAATCTGGAAAAAACTGGAAAAATCTGGAAAAGAAAAACAAAAAAAATTCAAGTCTGCAAAGTTGCGTGACGGTGTTTGCGGACAAACGCCTGCGCCTCTTCATTTCGCGTTGTGCCTCGCGGTTTTTAATTATAAAATTTTTTAATAATTAGAATTCAGTACACATTCTTGTAAAGCCTTCCTATCTTGTGTCACTGAAGCTTTTTCGCAAAAAAAGCGCTTCCAAAGTAGCAGAAGAAACTGAGAAAAATGAATAAAACATTAAATGTCTGCGCTAAA

>chrV:1226200-1226599_*madd-2*

TGTTTTACTGTTGGCTCGAAAAATATTGTTATGTGGGCCTTTTTTTTACCAAATCATATTGTTGAAAATTTTCAGGATTTTACAAAATTGAGAATTTTAATTTTTTTTTTCTTACTATTAATACTTGATCAGACGCTTGCCGCGCTTCCGGCGCGGCCGACGACGTTCTAAAATTTTCCCAAATTTTCCAGAAGGTTCTAGAACATTCCAAAATTTGCTCGAATTTTCTAGAAGGTTCTAGAACATTCCAGAATTTTCTCAAACCTTCCAGAAGGTTCTAGAACATTCCAGAATGTTAAAAAAAAAGAAAATTTTGAATTCCCGCTAAACAACCTTATCAGAAATTTTGAATTTCTCGCGAATTTGGGATGAAGTCACAAAATGGGTTCATTTTTTGGCA

>chrV:1278400-1278999_*tank-1*

AATTTTTCTGAAACTACCGTAATCCTACAGTACTCCTACCGTACCACTATTGTACCACTACAGTACCCCGACTATATCCCTACACTAACCCAACTCACTATCCCTCCAGAAGCTAAAACTTTGCAGACTACAAAGACTACAAAGACTACAAACTATGGACAAACGGAATAAACGCTTTATATATAGTAAATGATATATAAAGCGCAGTTCCATCTCTATGATGTTTTAGAACCTTCTGGAAAATTCGAGAAAACTCTATAATGTTCTAGAACTTTTTGGAACATTCGAGAAAAATTCTGAAATGTTCTAGAACACTCTGGAAAATTAGAGAAAAATCTGGAATGTTCTAGAACCTTCTGGAAAATTCTGGTTGAAATCAAATTGATAGGATATGTTACTCACTCCTTAATGGGTCGTGGGAATGCAGCATTCTTTACAATTTTGAGCATTTCGCTTTTCATTCTTCGATTCATTTTCATCGCGGCCAACTCAATCGGATTCGGACTTCTCTTATCAATCAGACATTGTACAATAGCGGTCTTGTTGGCTGCAGCCAAATCTCCAAGAAGTTCCACATTCTCCCAGGCGAGCGGCTCAATG

>chrV:1355200-1355799_*glb-33*

AAAATTTGTTGCTCTTTGCCTAAAAAATGATTGGTGCTCTTTATGCCAAATCGATCAAATATCGCATTTGCTTCGTATAGATTTGGAAATTTTCGATTAATGTACAGTGTGTGATAGGGGTACTGAAGTTTTACTTGGATGTTTGTCAGAAACTTCTTTCTTTTATATATATACTTGAAACCAGCCAGAAGGTTCTAGAACATTCTAGAATTTTCTCGAGCATTCCAGAATTTTCTCAAACTTTCTAGAAGGTTCTAGAACTTTCCAGAATTTTCTCAAACTTTCTAGAAGGTTCTAGAACATTCCAGAATTTTCTCGAATTTTCCAGAAGGTCCTAGAACATTCCAGAATTTTTTCGAATTTTCGAAACCGTTCTTGAACATTCCAGAACTTTCCTAGAATTTTCCAAAATTTCTAGAACATTTCAGAAATTTCTTGAGTTTTCCAGAAAGTTCTAGAACATTCCAGAATGTAAAAATTTTTAGGGAAAACTTTTGATAAACGCTCAGCGGACATGTATCCGGGTCTGCGGTGACCTACTCAAACCCCTATAGTAGGTCATTTGTCCCAACTTTCAACACTTCTATAAGAATGAGTGAG

>chrV:1530800-1531199_K02H11.4

AGTTTATAAACAAATTGTAAATTATTTATGTAAGTGTAGAAAAACATTGACTTGAATTGTTTAGTTACACACTTATTGATAAACTACCAGCCGCTCATTTTATCGAATTTTTCAGAAAGTTCTAGAACATTCCAGAATTTTTTCGAATTTTCCAGAAGGTTCTAGAACGTTCCAGAATTTTCTCGAATTTACCAGGAAGTTCTAGAACATTCCAGAATTTTTTCGAATTTTCCAGAAGGTTCTAGAACGTTCCAGAATTTTCTCGAATTTACCAGGAAGTTCTAGAACATTCCAGAATTTTTTCGAATTTTCCAGAAGGGACTATAACATTCTAGAATTTTCTCAAAATTTACAGAAGGTTTCCAGGAGGAATTAGAACATTGACCATTAAAATAAAACA

>chrV:1804800-1805399_*hsp-16.41*_*hsp-16.2*

TCAGAAGACTCAGATGGAGAGATTCTGCAGACTGGAGCAAATTGGCGTTCCATCAGAGCCATATCTCTCATAAGATCACCAAAAACGGAACGTTGAGCTGGACGGAAATAGTGGTAAAGTGACATGATTATAGTTTGAAGATTTCTAATTTCACAATTAGAGCAAATGTTGTTCGGTATTTATTTTCAACGGTATTTATACTATTTTCCACCTTTTTCTAGAACATTCGAGCTGCTTGTTGCAAAAGGAGGGCGACTCACATTCGGTACATGGAAAAGTAGTGTACACAATAAAGAGACCCAGATACATTTTCCGTCTGCGTCTCTTTGCACCCACCGGGAGTATTTTCAAACGAATGCATCTAGGACCTTCTAGAACATTCTGTAAGGCTGCAGAATGCGGGTATATAAGGAAAGCGGGCTCAGAGGAAGCCAACACGCTTTGTTCTAGTGCATCTAAAAAACTTCGAAAATGCTCATGCTCCGTTCTCCATATTCTGATTCAAATGCTCTTGATCATTTCTTGGACGAACTCACTGGATCTGTTCAATTTCCGTATTGGAGAAATGCTGATCACAACTCATTCAATTTTTCCGATAAT

>chrV:2630800-2631199_W02H5.11

AAAATTTCCTCAAATGTTTTTTTCTGTGACTTTACTTGAAGAAACTTTAAAAAAACCCCTATTTTTCAAAAGCTATTTGAGAACTACCGAAACAATCAGGGGTACGCCAGTCGGAGAAAAAAATTTCTGGAATGTTCTAGAACTTTCTGAAAACATCGAGAAATTTTTACAATGTTCTAGAACCTGTTGGAAAATTTGAGAAAGTTCTGAAATGTTCTAGAACATTTTTAAAAGAATTTCAACAAATTCCGATACAAAAAATTAGCTCAAAAACTATTAAATATTTTTCGAGACTCTTACTAATATTTCAAAATTTCTAGAATTTCCTTTAAAATTCAAAAACAGTTGAGATTTTCTACATACTTAAAAATGTAATAATCGTTCCGTAAAACTATCGAAT

>chrV:3520400-3520799_*irg-1*

TGTAGAGGTACTGTAGGAATACTGTAGGATTACTGTAGTTTGGGAAAAATTGACTTTTCGTCTATTGAACGGATATTGGAAACTTTGAGAAAATTCCGGAATGCTCCAGAACCTTCTGGAAAATTCGAGAAAATTCTGGAATGTTCCAGAACCTTCTGGAAAATCCGAGAAAATTCTGGAATGTTCCAGAACCTTCTGGAAAATCCGAGAAAATTCCGGAATGCTCCAGAACCTTCTGGAAAATTCGAGAAAATTCTGGAATGTTCCAGAACCTTCTGGAAAATTTGAGAAAATTCTGGAATGTTCCAGAACCTTCTGGAAAATTTGAGAAAATGCTGGAATGTTCCAGAACCTTCTGGAAAATTCGAGAAAATTCGTGGTGAGACCCTTCGTGGTGAGA

>chrV:4050600-4051399_*srh-169*

TGAAGGGATATTGCTTTGGGGATAGTGGGGGGATATGGCCGGGGTACTGTAGTAATACTGTAGGGGTACTGTAGGAATGTTTTTGAACCTTCTGAAAAATTCGAGAAAATTTTGGAATGTTTTTGAACCTTCTGGAAAATTCGAAAAAATTCTGAAATGTTCTAGAACCTTCTGGAAAATTCGAGAAAACTTTGGAATGTTCTAGAACTTTCTGGAAAATTCGAGAAAGTTCTGGAATATTCTAGAACTTTCTGGAAAATTCGAAAAAATTCTTGAATGTTTTTGAACCTTCTGGAAAATTCGAGGAGATTCTTGAATGTTTTTGAACCTTCTGAAAAATTCGAGAAAATTCTGGAATGTTTTTGGACCTTCTGGAAAATTCGAGAAAATTCTAGAATATTCTAGAACCTTCTGGAAAATTCGAGAAAATTCTTGAATGTTTTTGAACCTTCTGGAAAATTCGAGAAAATTCTAGAATATTCTAGAACCTTCTGGAAAATTCGAAAAAATTCTTGAATGTTTTTGAACCTTCTGAAAAATTCGAGAAAATTCTGGAATGTTTTTGAACCTTCTGGAAAATTCGAGAAAATTCTAGAATATTCTAGAACCTTCTGGAAAATTCGAGAAAATTCTGGAATGTTTTTGAACCTTCTGGAACAATATTGATATTGCCTTTGTCACACGTTTTTTTGGGTAAAATAACAAAACAGGTTTTTTTTTTCGTTTTTGTCGAAAGTTTTAGATACAAAGATACAGTGCCTAATTTTAATTCTATTATTTTTAGAAATCATTGGGAAGAA

>chrV:5641400-5641799_*srx-117*

GGAAATTCAAATTTTCAGTAAAAAAATTTTGGCGGGAAATTCAAATTTTCAGTGAAAAAAAATTTAGCGGGAAATTCAAATTTTCAGTGAAAAAAATTTGGCGGGAAATTCAAATTTTCAGTGACAAACATTTTGGCGGGAAATTCAAATTTTCAGTGAAAAAAATTTGGCGGAAAATTCCAATTTCTGAGAAAAATCGAGAAATGTCTGCAATGTTCCAGAAGTTTTTAGAAAATTCGAGAAAATTCCGGAATGGTCCAGAATTTTCTAGAAAATTCGGGAAAATTCAGGAATATTCCAGAACTTTCTAGAAAAATCGGGAAAATTCTGGAATGTTCCAGAACTCTCTAAAAAATTCGATAAAATTCTGAAATGTGCAATTTTGTGGAAAAATTCAAGA

>chrV:8936000-8936599_*unc-23*

TAAAAAAATCGGAAAAATATTACGAAAAAATGATCGATATCAAAAAAAAATTTTCACTTTGAAAAGTAGACACAGAAATAAAAGGTGCAGCACAGCTTCGCAGTGAAAAATATATTATAGTTGCCAAAATTTATGTTTTTGGAAAAGCTGAAAAACAACAAAACACCTCGTAAAACAGTGCCTATCATTTTCATCTCTGAATCTTCGCGTTTCACTGATTGTCTAGATTCTCAAACCAATCTCTGCGTCTCGTTTCCATATACACTGTCTCCCATCATATTTCTTCTCGAAGACTCCCGAACTGCGGAGAACTGAATGGGCGTGGCCGGGCGCCCGCCATCAATTGTTTCGTAACTCTCTTTTCTTTGCCTCTTTTTTCCGAGAGAATAGGAGACTCAGACCGTTTGTGTCAAACCGCCACCAGTGACTGACTGTCAAAACTTTTTGTTTCGGTGTCTGTGTCGCTGCTGCTTTTCCCCTGCATTGCTTTCCCACCTCTTTCTCCTCCAGAAAAGGCGAACCGGTCCGAGTGTTCCGCGCATATTGAAAGCAAACGAGACATCGACCTTCTCACATCCCATATTGCCCCTATTTTACCCT

>chrV:13440000-13440799_*col-162*

ATTTTCTGAGAAAAATATTTTGGCGGGAAATTCAAATTTTTTGTGAAAAATATTTTGGCGAGAAATTTAAATTTTCTGAGAAAAATCTTTCGGCGGAAAATTCAAATTTTCTGAGAAAAATATTTTGGCGGGAAATTTAAATTTTCTGAAAAAAAAATTTCGGCGGGAAATAGTTTTCTGAAAATTCGAAAATTCTGGAATGTTCCAGAACTTTCTAAAAAAATCGGGAAAATTCTGGAATGTTCCAGAACTGGGAAAATTCTGGAATGTTCCAGAACTTTCTAGAAAAATCGAGAAAATTCTGGAATGTTCCAGAACTTTCTAAAAAAATCGGGAAATTTCTGGAATGTTCCAGAACTTTCTAGAAAAATCGGAAAATTTCTGGAATGTTCCAGAACTTTCTAGAAAAATTGGGAAATTTCTGGAATGTTCCAGAACTTTCTAGAAAAATTGGGAAAAGTCTGGAATGTTCCAGAACTTTCTAGAAAAATCGGGAAAATTCTGGAATGTTCCAGAACTTTCTAGAAAAATCGAGAAAATTCTGGAATGTTCCAGAACTTTCTAAAAAAATCGAGAAAATTCTGGAATGTTCCAGAACTTTCTAGAAAAATCGGGAAATTTCTGGAATGTTCCAGAACTTTCTAGAAAAATCTAGAAAATTCTGGAATGTTCCGAAAAATTGAGCTTAGAGCTTTAGAAGAGGTAGTTATTTGGGAGTTGATGGGGGAGCAAGTCAAGGTACTGTAGTGGTACTATAGGGGTACTGTAGGTATACGGTAGGGTTACTGTAGTTTTGGAAA

>chrV:14685600-14685999_*hsp-90*/*daf-21*

TTTTTTTTGGTGCAAAAATACCAAAAAAACGTCTTTTCGACGAAGTAATCTGAAAAAAATGAAGAAAAAACAATTCAAACTAAAAACAAGAAAATGTTTGTGGCCTAGTTACCTATTCTGCAAAACTTCGGCCAGGTGCTGAAAACGCGCTCTATTGACAATTTTCGTCGAGAATTTTCCGCCGAAAAGGGCAATTTGAAAACCAACCAATCAAATTGAAAGGCCACGCCCTCCTTCGAGAACATTTCGCATCTTGTTCAAAATTTAGACCTTCAATTACGCCTCAGAGCTCTGATATTCCCAGTTCTTTCGGTTTTCGGGTGAAGATTTTTGGTTTTCCTCTCACTAATATGGCTTTTATGTTAATCACGGATCATTGTTTTTGATTTTTAAAGGTTTT

>chrV:15295400-15295799_*cpr-3*

TTCCCGCCCATATTTTTAAGGGTCCCACCACGATGGGTCTCGCCAGTTCCAGGTGGTACTTAAGCTAACAAAAAGTTTCTCAGAAAATTTGAATTTCCCGCCAAAAATGTTTTCTCAGAAATTTTGAGTTTTCGGCCAAAATTTATGGGTCTCACCACGAATATTCCAGAATTTTTTTCGAATTTTTCAGAAGGTTCTGGAACTTTCCAGAAGGTTCTGGAACTTTCCAGAAGGTTCTGGAACTTTCCAGAAAGTTCTGGAACATTCCAGAATTTTCTCGAATTTTCCAGAAAGTTCTAGAACATTATAGAATTTTCTGGAATTTTCCAGATGTTTCCAAAACCAATATTGAAATTCCCGCCAAAATCTTTTTCTCAGAAAATTTGAATTTCCCTCCAAA

>chrV:15320600-15321599_*nhr-291*/ZK1037.13

TTTTTATTTCGCTCTCTGACATTGATGAAGACGTTCAAATGCCCGAAATGTTTCGAAAAAGTTTTCAAGAAGAAAAGCAGTGATTATTAAAAACCAGCCGCTGACCGCGCCTACGGCGCGGGCAACGACTGGCACCATTAAAAGTATTTGACACACATACATTTCCAGAATTTTCTTTTTCGATTTTTCTAGAAAGTTCTGGAACATTCCAGAATTTTCTCGATTTTTCTAGAAAATTCTGGAACACTCCAGAATTTTCCCGATTTTTCTAGAAAATTCTGGACCACTCCAGAATTTTCTCGATTTTTCTAGAAAGTTCTGGAACATTCCAGAATTTTCCCGATTTTTCTAGAAAGTTCTGGAACATTCCAGAATTTTCCCGATTTTTCTAGAAAGTTCTGGAACATTCCAGAATTTTCTCGATTTTTCTAGAAAGTTCTGGAACACTCCAGAATTTTCCCGATTTTTCTAGAAAGTTCTGGAACATTCCAGAATTTTCTCGATTTTTCTAGAAAGTTCTGGAACATTCCAGAATTTTCTCGATTTTTCTAGAAAGTTCTGGAACATTCTAGAATTTTCTCGATTTTTCTAGAAAGTTCTGGAACATTCCAGAATTTTCTCGATTTTTCTAGAAAGTTCTGGAACATTCCAGAATTTTCCCGATTTTTCTAGAAAGTTCTGGAACATTCCAGAATTTTCCCGATTTTTCTAGAAAGTTCTGGAACATTCTAGAATTTTCTCGATTTTTCTAGAAAGTTCTGAAACATTCCAGAAAATCATTTTCAGAAAATTTAAATTTCCCGCCAAAATATTTTTCACAGAAAATTCAAAGTTCCCGCCAAAATTTTGGGTCTTACCACGGTGGGTCTCACCACGACGGGTCTCACCACGATGGGTCTCACCACGATGGGTCTCACCACGATGGGTCTCACCACGATGGGTCTCACCACGATGGGTCTCACCACGATGGGTCTCACCACGATGGGTCTCACCACGATGG

>chrV:15942000-15942399_*srw-53*

CCCTATCCACTGAACTACACGTTTTTAAAAAGAGAGAAAACTGTAGTTTTAGAAAAATAAACAATTTTTCGAAAACATTTTAGCGGGAATTCAAATTTAAATTTTCCGAAACCATTTTGACGGGAAATTCGAGAAAATTCTGGAATGTTCCAGAACCTTCTTGAAAATTTGGGAAAGTTCTGGAATCTTCTTGAATCATCAGAAAAATTGGGGAAAATTCTGGATTGTTCTAGAACCTTCTCGAAAATTCGAGAAAATTTTGGATTGTTCCAAAATCTTTTGAAAAATTCGAGAAAATTCTGAATGTTCGTGGCGAGACCTATCGTGGTTAGACCCATCATGGTGAGACCCTTAAAACTTTCGGTGGGAAATTGCTGGAAATAAATATTTTGCCGCGAAG

>chrV:19282000-19282399_*fbxb-117*

GTGTGAGTGGGAAATCAGCGGATGATTATCGCTGGATTGTGGGCGATTCTTGCCGATAATTATAATCCGCAAAGTTGGGGCGGAGGACCTCTACGGAGGCGAAGTCACAACAGTTTCTAGAAAAGTCTAGAAACTTCTCGAACTTTTTAAAATCGTGGCGAGGCCCATCCATTTTTAGCGGGAAATTCAAATTTTCTAAGAGAAATGTACAAATCTATTGTGTCCATAACTTTCTGGAAAATTCGAGAATGTTCTTGAACCATCTGGAAATATCGAAAAAACTCTGGAATGTTCTAGAATCATCTAGAACTTTCTAGAAAACTTGAGAAAATACGGAAAGGTTCTAGAACTTTCTGGAAAATTCGAGAAAATTTTGGAATCGTTTTGAACTTTCTATAAA

>chrV:19296000-19296599_F55C9.6

TGTCCGAAAAAGTTTTGTGGATAATTCGAAAAAAAGTACAAAAACTTATGGGACGACCTGATAGAAATAACTGAAAACTTCTAGAATTTTCTGAAAAAATCTGGAAACTTTTAAGAAAATCTGGGAACGTCTAGAAAATTTAAGACACTTCTGGAACTGTTTAGAAATAGCTGGAAACTTCCGGAATTTTCTAGAAAAATCTGGAAACTTCTAGAAAAATTCAAGACTGGTAGAACTGTCTAAAAAAACCTGCAAACTTCTTAACTTTCCTAGGAACATTTGGAAACTTCTAGAAAAACCTGGAAACGTTTAAAGTTTTCTGAGAAAATCTGGAAACTTCTAGAAAATTTAAGACATTTCTAGAACTTTGTAGAAATAACTGAAAACTTCTGGAATTTTATGAAAAATCTGGAAACTTTTAAGAAAATCTGGAAACTTCTAGAAAATTTAAGACACTTCTAGAACTGTTCAGAAATAGCTGAAAACTTCTAGAATCTTCTGAAAAAAATCTGGAAACTTTTAAGAAAATCTGGGAACGTCTAGAAAGTTTAAGACACTTCTAGAACTTTGTAGAAAAAACTGAAAACTTCTAGAATTTTC

>chrV:20164400-20164999_F19B2.5

CAATAATGAAATTTTCGAAAACATTTTGGGGGGGCAGAAATCGGAAAAGTACCGAAATTTGTCAACTTTTGAAATAATAAGGCTTGAAGAGAAAGGGTACACGACAGAATGATAAATTGGGGCAATGGGAAAAGCTGGTGAAATATTAAGGCACAAAAAGATATATGTGACTCCTATTGTCTAATTTTATAGTACCAGGGAGGGGCGGAGCAAGATGCCAGGCAGCTTCGGGAAGGTTCGAGAAGATAGGCGGTGCCAACTGTTGTGAATTTTGCAAACTGATATTTATGATTGCATCACTGCTTTTCTTATCAAGACAACACGGACGGGTATACTGTTTTAGGGAGAGGAAAATTCGGGAAGAGTCGAGAATTTGGCGGCAGTTTGTGAGTCATTTGACTGTGCATTTACGTTTAAGTAGATAACAGTTGCCACGTTTCGTTTTTTCATGACATAAATTATTTTTCCCATGCAGAAATGTTATCGAGAAAGATAAAGTTCAGCGAAATAAACATAAATAATTTAACATATCAAACTAGTAAACCAAGAATCATATCAATCTCACAATCAACAAAGTTTCATACAGAAACAAAACAACCC
