# Supplementary material for: Whole genome profiling of short-term hypoxia induced genes and identification of HIF-1 binding sites provide insights into HIF-1 function in Caenorhabditis elegans
Source: PLoS One. 2024 May 14;19(5):e0295094. doi: 10.1371/journal.pone.0295094 (PMC11093353; doi:10.1371/journal.pone.0295094)
Supplement: S6 File — (DOCX) [file pone.0295094.s027.docx]

**S6 File. Sequences co-immunoprecipitated with HIF-1** **on chromosome X.** The HRE similar sites were color coded as red on the reference Watson strand, and as green on the Crick strand.

>chrX:330600-331199_M02E1.1

GGTACTGTAGTAGTACTGTAGAGGTACTGTAGGAGTACTGTAGGATTACTGTAGTTTAGGAAAAAATGAGTTTTTGTCTTTTGAAGAGATATAGGTTTGGGGTTAGTGGTGGGATATGGTCGGGGTACTGTAGTAGTACTGTAGAGGTACTGTAGGAGTACTGTAGGATTACTGTAGTTTAGGAAAAAATGAGTTTTTGTCTTTTGAAGGGAAATTGGAAACTTCGGGAAAATTTTGGAATGTTCCAGAACCTGGAAAATTCTGGAAAGCTCTAGAACCTTCTGGAAAGTTCGAGAAAATTCTGGAATGTTCTAGAACCTTCTGGAAAATTCGAAAAAAAAATTCTGGGATTTTCTAGAACCTTCTGGAAAATTCTGAAAAGCTTTAGAACCTTCTGGAAAGGTCTAGAAAATTCTGGAATGTTCTAGAACCTTCTGGAATATTCGAGAAAATTCTGGAATGTTCTAGAACCTTCTGGAAAATTCGAAAAAAAATTCTGGAATTTTCTAGAACCTTCTGGAAAATTCGAGAAAATTCTGGAATGTTCTAGAAGCTTCTGAAAAATTCGAGAAAATTCTGGAATGTTCTAGAACCTTCTGG

>chrX:443200-443999_F52D1.2

TAGAACCTTCTGAAAAATTCGAGAAAATTCTGGAATGTTCTAGAACCTTCTGGAAAGTTCGAGAAAATTCTGGAATGTTCTAGAACCTTCTGGAAAATTCGAGAAAATTCTGGAATGTTCTAGAACCTTCTGGAAAGTTCGAGAAAATTCTGGAATGTTCTAGAACCTTCTGGAAAATTCGAGAAAATTCTGGAATGTTCTACAACCTTCTGGAAAGTTCGAGAAAAAACTGGAATGTTCTAGAACCTTCTGGAAAATTCGAGAAAATTCTGGAATGTTCTAGAACCTTCTGGAAAATTCGAGAAAATTCTGGAATGTTCTAGAACCTTCTGGAAAGTTCGAGAAAATTCTGGAATGTTCTAGAACCTTCTGGAAAATTCGAAAAAAAAATTCTGGAATTTTCTAGAACCTTCTGGAAAATTCGAGAAAATTCTGGAATGTTCTAGAAGCTTCTGAAAAATTCGAGAAAATTCTGGAATTTTCTAGAACCTTCTGGAAAATTCGAGAAAATTCTGGAATGTTCTAGAACCTTCTGGAAAGTTCGAGAAAATTCTGGAATGTTCTAGAACCTTTTGGAAAATTCGAAAAAAAAATTCTGGAATTTTCTAGAACCTTCTGGAAAATTCGAGAAAATTCTGGAATGTTCTAGAACCTTCTGGAAAATTCGAGAAAATTCGTGAAAATTCTGGAATTGTTGTGGTGAGACCTATCGTGCTGAGACCCATTGTGGTGAGACCCTTAAAAATTTTGGCGGGAAATTCAAAATTTATGAGAACATTTTTTTGAGGGAAATTCAAGTT

>chrX:516800-517199_B0310.3

GTCTCGCCACGATGGGTCTCGCCACGATGGGTCTCGCCACGATGGCTCTCGCCACGATAATATCGCAGCAACATTTTTTTAATTTTCCAGAAGGTTCTAGAACAATCCAGAATTTTTCGAATTTTCCAGAAGGTTCTGGAACATTCCAGAATTTTCTAGAATTTTCCAGAAGGTTCTAAAGCTTTTCAGAATTTTCCAGAAGGTTCTGGAACGTTCTAGAATTTTCCAGAAATTCCCAGATGGTTCTGGGACATTTCAGAATTTTCCCGAAGTTTCCAATACCCCTTCCCAAGACGGAAAGTCAATTTTTCATAAACTACCGTAATCCTACCGTACTCCTATAGTACTCCTACAGTACTATTACTGTACCCCGACCATATCCCACCACTAACCCCAAACC

>chrX:1809800-1810399_C14E2.3

CTCAGTTTCTTTCTAATTCCTTTTTGAATTTTTAAAATTTTCCACTAGGGTCTAGAACATTCCAGAATTCTTTTGAATTTTCCAGAACCTTTTAAAACTGTCCAGAATTTTTTAAAATTTTCCAGAACCTTCTAAAACTTTCCAGAATTTTTTTTCAATTGTCCAGAAGGTTCTTGAACATTCCAGATTTTTTTTGAATTTTCCAGTAGGTTCTAGAACATTCCAAAATTTTTTAAATTTTCCAGAAGGTTCTAGAACATTCCAGAATTATTTCAAAATTTCCAGAACCTTCTAGAGCTTTACAGAATTGTTTCGAATTTTCTAGAACCTTTTAGAACATTTCAGAATTATTTCAAAATTTTACAGAACCTTCTAGAACAATCCAGAATTTTCTTACATTTTCCAGAACCTTTTAGAACATTCCAGATTTTTTTTAAAATTTTATAGAAACTTCTAGAATTTTTACAATTTCCCCACTGGCTTTAATATTTAAATAAAAATGTAAAAATGAAAAATATATCAAAATAGCTTTCAGACCATTGAATTAGATACAAATTTAAAATTAAAAAAAAATTTTGAAAATTTACATTAAATTATTAA

>chrX:1862200-1862399_T26C11.8

AAAAAGCTTTAGAACATTACAGATTTTTTTCGAACTTTCCAGAAGGTTCTAGAAGATTTCAGAAATTTTACGAATTTTCTAAAAGGTTCTAGAACATATCCGATTTTTTTTGAATTTTCCAAAAGGTTCTAGAACATTTCCGAAATTTTTCAAACTTTCCAGAAAGTTCTAGAGCACCCCAGAATTTTTCGTGAATTTTC

>chrX:2002600-2002999_Y40A1A.3

CTCAGTTTCTTTCTAATTCCTTTTTGAATTTTTAAAATTTTCCACTAGGGTCTAGAACATTCCAGAATTCTTTTGAATTTTCCAGAACCTTTTAAAACTGTCCAGAATTTTTTAAAATTTTCCAGAACCTTCTAAAACTTTCCAGAATTTTTTTTCAATTGTCCAGAAGGTTCTTGAACATTCCAGATTTTTTTTGAATTTTCCAGTAGGTTCTAGAACATTCCAAAATTTTTTAAATTTTCCAGAAGGTTCTAGAACATTCCAGAATTATTTCAAAATTTCCAGAACCTTCTAGAGCTTTACAGAATTGTTTCGAATTTTCTAGAACCTTTTAGAACATTTCAGAATTATTTCAAAATTTTACAGAACCTTCTAGAACAATCCAGAATTTTCTTACATTTTCCAGAACCTTTTAGAACATTCCAGATTTTTTTTAAAATTTTATAGAAACTTCTAGAATTTTTACAATTTCCCCACTGGCTTTAATATTTAAATAAAAATGTAAAAATGAAAAATATATCAAAATAGCTTTCAGACCATTGAATTAGATACAAATTTAAAATTAAAAAAAAATTTTGAAAATTTACATTAAATTATTAA

>chrX:16100400-16100999_ZK1073.2

TTTGTCTTTTGAAGTGATATTGGTTTGAGGTTAGTGGTGGGATATGGTCGGGGTACTGTAGTAGTACTGTAGGAGTACTGTAGGATTACTGTATTTTTGAAAAAAATTGGCTTTTCGTCTTTTGAAGTGATATTGCTTTGGGGTTAGTGTCGGGATATGGTTGGGGTACTGTAGTTGTACTGTAGAGGTACTGTAGGAGTACTGTAGGATTACTGTAGTTTGGGAAAAATTGACTTTTCGTCTATTGAACGGATATTGGAAACTTTGAGAAAATTCCGGAAGGCTCCAGAACCTTCTGGAAAATTCGAGAAAATTCTGGAATGTTCCAGAACCTTCTGGAAAATCCGAGAAAATTCTGGAATGTTCCAGAACCTTCTGGAAAATTTGAGAAAATGCTGGAATGTTCCAGAACCTTCTGGAAAATTTGATAAAATTCTGGAATGTTCCAGAACCTTCTGGAAAATTTGAGAAAATTCTGGAATGTTCCAGAACCTTCTGGAAAATTCGAGAAAATTCTGGAATGTTCCAGAACCTTCTGGAAAATCCGAGAAAATTCTGGAATGTTCCAGAACCTTCTGGAAAATTTGAGAAAATGCTGGA

>chrX:17287800-17288799_C33E10.8

TATAAAGGAGAAAATTGGATTTTCCAGCCAAAAAGTTTCTATAAGAAAATTTGAATTTCCAGCCAAAATTTTTTATCACAAAATTTGAATTTCCCGCCAAAAATTATTCTCAGAAAATTTGAAATTCTCGCCAAAAATTGTTTTGATAAAATTTCCCGCCAAAAATTTTTTATCAGAAAATTTTAATTTCTCTCCAAAACTTTTTCTCATTAATTTTGAATTTCCCGGTCAACGTTTTACGATGGCTCTCGCCACGAACATTTCAGAAATTTGAATTTTCTCGAATTTTCCAGAAGGTTCTGGAACAGTCTAGAATTTTCCAGAAATTTCATGAAACGTCTGGAACATTCAAGAACTTTCCTGATTTTTCCAGAAGGTTCTTTCCGGCGTTTTCCCGAACTTTTCAAATGTTTCTAGAACATTCCAGAGTTTTCCCAATTTTTCCAGTTGGTTTTCGATTATTCCAGAATTTTCTCGAATTTTCCGGAAGGTTCTAGAATATTCTAGAATTTTCTTGAAACTTCTGAAAGGTACTGAAACAAATAAAGTTTCTCTAAAAATTTGAAGTTCCCGTCAAAAGTCTTTTTTAGAAAAGTTGAAAATCCCGCTAAAATGCCTTTTCTCAGAAAATTTAAACTTCCCGCCAATTTTTTTAAACGACGTGTCTCATCACGATGGGTCTCACCACGATGGGTCTCGACACGAACATTTCAAAATTTTCTCGAATTTTCCAGAAGGTTCTAGAACATTCCAGAAATTTCTCGAATCTTCCAGAAGGTTCTGGAACATTCCAGAATTTTCTCGAATTTTCCAGAAGGTTCTAGAACATTCCAGAATTTTCTCGAGTCTTTCAAAAGATTCTGGAACATTCCAGAAATTTCTCGAATTTTCCAGAAGGTTCTAGAACATTTCTCGACAAAAGACAAGTTTCCCTTCAAAAGACTACAGTAATCCTACAGTACTACAGTACTGTAGTAACCCTACAGTACCTCTACAGTAC
