# Supplementary material for: Whole genome profiling of short-term hypoxia induced genes and identification of HIF-1 binding sites provide insights into HIF-1 function in Caenorhabditis elegans
Source: PLoS One. 2024 May 14;19(5):e0295094. doi: 10.1371/journal.pone.0295094 (PMC11093353; doi:10.1371/journal.pone.0295094)
Supplement: S7 File — (DOCX) [file pone.0295094.s028.docx]

**S7 File. Detailed protocol for HIF-1 chromatin immunoprecipitation (ChIP).**

This ChIP protocol was modified from Zhong et al. [1]. The strain used for the ChIP experiments was ZG434 (*egl-9(sa307);iaIS28[Phif-1::hif-1a::Myc::HA];hif-1(ia04)*). Because HIF-1 was degraded within minutes upon reoxygenation [2, 3], the ChIP experiments were performed in the *egl-9(sa307)* loss-of- function mutant background to stabilize HIF-1 and maintain its activity. To take advantage of the commercially available ChIP grade anti-HA antibody, an HA-tagged *hif*-1 transgene *iaIS28[Phif-1::hif-1a::Myc::HA]*) was used [4], and the endogenous *hif-1* gene was knocked out. Harvesting enough synchronized worms for HIF-1 ChIP experiment was laborious due to the egg-laying defect inherent to the *egl-9(sa307)* loss-of- function mutation. For one harvest, 650-780 adult worms were manually picked onto NGM plates seeded with OP50 to lay eggs for 5-6 hours, about 10,000 synchronized L4-stage worms could then be harvested 58-60 hours after the eggs had been laid. These 10,000 L4-stage worms were washed off plates using M9 and transferred to a 15 ml conical tube to centrifuge for 30 seconds at 4,000 rpm at 21°C. The supernatant was removed and the worms were washed once more with M9 and resuspended in a solution of 1.7 ml M9 with 97 ul 37% formaldehyde solution (the final is 2% formaldehyde solution) to cross-link protein and DNA at 21°C for 30 minutes with rotation. The worms were washed twice at 21°C with 1.7 ml 0.1 M Tris pH 7.5 to quench formaldehyde, followed by two washes in M9, and one wash in RIPA lysis buffer (50 mM Tris-HCl pH 8.0, 150 mM NaCl, 2 mM EDTA pH8.0, 1% Nonidet P-40, 0.1% sodium deoxycholate, 0.1% SDS) supplemented with protease inhibitors (Roche). The supernatant was removed and the worm pellet was stored at ‑80°C. The frozen worms were used for the ChIP experiments within two weeks. The ChIP-sq experiment were performed with two biological replicates. For each biological replicate, 5 tubes of worms, about 10,000 worms per tube, were thawed in an ice-salt bath. After thawing, 210 ul RIPA lysis buffer with protease inhibitors was added to each tube of worms. The samples were sonicated with a Branson sonifer microtip for 5-6 times in the ice-salt bath, each time for 2 seconds at 30% amplitude, cooling the samples for 2 minutes between each sonication. The lysates from these 5 tubes were combined into one 1.5 ml microcentrifuge tube and centrifuged at 14,000 rpm for 20 minutes at 4°C. Six hundred microliter supernatant was taken as the ChIP sample, 60 ul supernatant was taken as the input sample, and 3 x 60 ul supernatant was taken to 3 tubes as the sonication test samples. In the ChIP sample, 6 ul ChIP grade anti-HA tag antibody (Abcam, cat. no. ab9110) was added, rocking gently at 4°C for 1 hour. In each 60 ul input or sonication test sample, 2 ul 10% acrylamide carrier (Sigma, cat. no. A9099) and 150 ul -20°C ethanol were added, the samples were then kept at -80°C for 30 minutes to precipitate DNA. Twenty microliter protein A-Sepharose beads (Sigma) were taken into 1 ml ice-cold PBS supplemented with 0.5% BSA and centrifuged at 6,400 rpm for 3 minutes at 4°C. The washing was repeated twice. Washed protein A-Sepharose beads were added to the ChIP sample, shaking gently at 4°C for another 1 hour. One milliliter NET buffer (50 mM Tris-HCl pH 8.0, 150 mM NaCl, 2 mM EDTA pH 8.0, 0.1% Nonidet P-40, 0.02% NaN3) was added to the beads, shaking for 10 minutes at 4°C. The beads were centrifuged at 6,400 rpm for 3 minutes at 4°C. The NET washing was repeated twice. The beads were then washed with 1 ml TE (10 mM Tris-HCl pH 8.0, 2 mM EDTA pH 8.0) at 6,400 rpm for 10 minutes at 4°C. Newly prepared 210 ul elution buffer (1% SDS and 100 mM NaHCO3) was added to the beads to elute DNA. To elute DNA, The beads were incubated at 65°C for 30 minutes, vortexing the beads every 5 minutes during this interval. The beads were then centrifuged at 6,400 rpm for 2 minutes at 4°C and the supernatant was taken. Two microliter 40 ug/ml RNase was added to the supernatant to digest RNA at 37°C for 30 minutes. Two microliter 10 mg/ml proteinase K was added to the solution to reverse the cross-linking at 65°C for 4 hours. To purify DNA with Qiagen MinElute Kit, 5 volumes of Qiagen Buffer PB was added to the solution to bind to the spin column, and the DNA was eluted with 24 ul water. The purified ChIP DNA was stored at -20°C for sequencing. The input and sonication test samples were removed from -80°C and centrifuged at 6,400 rpm for 15 minutes at 4°C to pellet DNA. The DNA pellet was washed with 70% ethanol, air-dried and dissolved in 310 ul elution buffer (1% SDS and 100 mM NaHCO3). Three microliter 4 mg/ml RNase was added to the DNA solution to digest RNA at 37°C for 30 minutes. Three microliter 10 mg/ml proteinase K was added to the solutions to reverse the cross-linking at 65°C for 4 hours and 30 minutes. The input and sonication test DNA were then purified with the Qiagen MinElute Kit. The sonication test DNA was run in the 1.5% agarose gel to check the extent of sonication, the distribution should be 200-800 bp. The purified input DNA was stored at -20°C for sequencing in parallel with the ChIP DNA.

1. Zhong M, Niu W, Lu ZJ, Sarov M, Murray JI, Janette J, et al. Genome-wide identification of binding sites defines distinct functions for Caenorhabditis elegans PHA-4/FOXA in development and environmental response. PLoS Genet. 2010;6(2):e1000848. Epub 2010/02/23. doi: 10.1371/journal.pgen.1000848. PubMed PMID: 20174564; PubMed Central PMCID: PMC2824807.

2. Jiang H, Guo R, Powell-Coffman JA. The Caenorhabditis elegans hif-1 gene encodes a bHLH-PAS protein that is required for adaptation to hypoxia. Proc Natl Acad Sci U S A. 2001;98(14):7916-21. Epub 2001/06/28. doi: 10.1073/pnas.141234698

141234698 [pii]. PubMed PMID: 11427734; PubMed Central PMCID: PMC35443.

3. Epstein AC, Gleadle JM, McNeill LA, Hewitson KS, O'Rourke J, Mole DR, et al. C. elegans EGL-9 and mammalian homologs define a family of dioxygenases that regulate HIF by prolyl hydroxylation. Cell. 2001;107(1):43-54. Epub 2001/10/12. doi: S0092-8674(01)00507-4 [pii]. PubMed PMID: 11595184.

4. Zhang Y, Shao Z, Zhai Z, Shen C, Powell-Coffman JA. The HIF-1 hypoxia-inducible factor modulates lifespan in C. elegans. PLoS One. 2009;4(7):e6348. Epub 2009/07/28. doi: 10.1371/journal.pone.0006348. PubMed PMID: 19633713.
